# Efficient Multicomponent Synthesis of Diverse Antibacterial Embelin-Privileged Structure Conjugates

**DOI:** 10.3390/molecules25143290

**Published:** 2020-07-20

**Authors:** Pedro Martín-Acosta, Rosalyn Peña, Gabriela Feresin, Alejandro Tapia, Isabel Lorenzo-Castrillejo, Félix Machín, Ángel Amesty, Ana Estévez-Braun

**Affiliations:** 1 Instituto Universitario de Bio-Orgánica Antonio González. Departamento de Química Orgánica. Universidad de La Laguna Avda. Astrofísico Fco. Sánchez N° 2, 38206 La Laguna, Spain; pmartina@ull.edu.es (P.M.-A.); rosalyn_pf@hotmail.com (R.P.); 2 Instituto de Biotecnología-Instituto de Ciencias Básicas, Universidad Nacional de San Juan, Av. Libertador General San Martín 1109 (O), CP 5400 San Juan, Argentina; gferesin@unsj.edu.ar (G.F.); atapia@unsj.edu.ar (A.T.); 3 Unidad de Investigación Hospital Universitario Nuestra Señora de La Candelaria, 38010 Santa Cruz de Tenerife, Spain; belilorenzo2000@yahoo.es; 4 Instituto de Tecnologías Biomédicas, Universidad de la Laguna, 38200 Tenerife, Spain; 5 Facultad de Ciencias de la Salud, Universidad Fernando Pessoa Canarias, 35450 Las Palmas de Gran Canaria, Spain

**Keywords:** embelin, multicomponent reactions, privileged structure, antimicrobial activity

## Abstract

A library of embelin derivatives has been synthesized through a multicomponent reaction from embelin (**1**), aldehydes and privileged structures such as 4-hydroxycoumarin, 4-hydroxy-2*H*-pyran-2-one and 2-naphthol, in the presence of InCl_3_ as catalyst. This multicomponent reaction implies Knoevenagel condensation, Michael addition, intramolecular cyclization and dehydration. Many of the synthesized compounds were active and selective against Gram-positive bacteria, including one important multiresistant *Staphylococcus aureus* clinical isolate. It was found how the conjugation of diverse privileged substructure with embelin led to adducts having enhanced antibacterial activities.

## 1. Introduction

Natural products continue to play a pivotal role in the search for new therapeutic drug leads. They have inherent bioactivities and high bioavailability, probably because of their specific interactions with target macromolecules in living organisms. Thus, the chemical space defined by natural products may nicely overlap with biological space [1]. Therefore, structural motifs and core skeletons from bioactive natural products can serve for the synthesis of novel core skeletons with high biological relevancy. In this context, the natural benzoquinone embelin (1) is an attractive molecule since displays a good number of biological activities such as antimicrobial [2], inhibition of X-chromosome-linked inhibitor of apoptosis protein (XIAPS) [3], inhibition of mortalin-p53 interactions, and activation of p53 protein in tumor cells [4], inhibition of 5-lipoxygenase [5], antitumoral activity via activation of p38/JNK pathway [6] and antidiabetic activity [7].

**Scheme 1:**
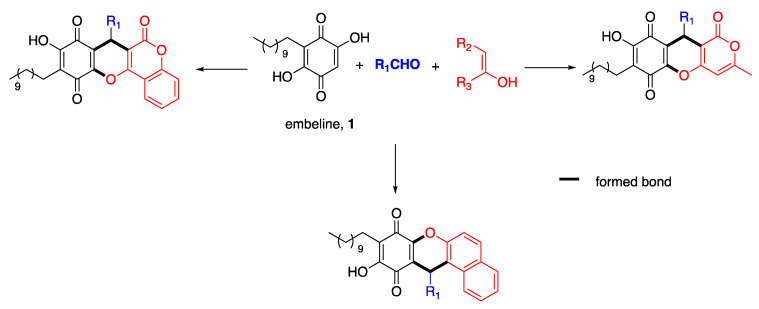
Structure of embelin-privileged structure conjugates.

Thus, this benzoquinone represents a good starting scaffold for the preparation of a structurally diverse collection of embelin derivatives. To assure the biological relevancy of this library we combine the use of privileged structures and complexity-generating reactions such as multicomponent reactions [8,9,10,11,12]. Privileged structures are defined as a single molecular framework able to provide a series of ligands for diverse receptors and have been extensively utilized in rational drug design owing to their potent biological activities [13]. Thus, from a domino Knoevenagel–Michael addition–cyclization–dehydration reaction using embelin (**1**), aldehydes and antibacterial privileged structural motifs as source of nucleophilic carbons, we can access to a library of dihydropyranbenzoquinones embedded with privileged substructures (Scheme 1). Moreover, this library may provide new compounds with great potency against both drug sensitive and drug-resistant Gram-positive and Gram-negative organisms.

## 2. Results and Discussion

We selected the following molecular frameworks frequently observed in natural products and synthetic drugs with antibacterial activity [14,15]: 4-hydroxy-2*H*-pyran-2-one (**2**), 4-hydroxycoumarin (**3**), and 2-naphthol (**4**).

**Figure 1:**
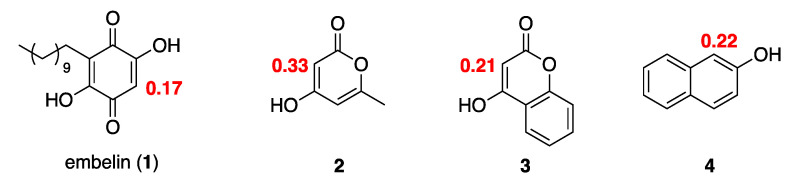
Location and highest value of the Fukui function (*f*_k_^−^) for compounds (**1–4**).

Since in the presence of an aldehyde both the embelin and the mentioned compounds (**2**–**4**) having nucleophilic carbons can react to afford the corresponding quinone methide intermediate via Knoevenagel condensation, we calculated the Fukui function in order to explore, which one shows the highest nucleophilicity. Fukui function is one of the widely used local density functional descriptor to model chemical reactivity and site-selectivity [16]. The local (condensed) Fukui functions (*f_k_*^+^, *f_k_*^−^, *f_k_*^0^) are calculated using the procedure proposed by Yang and Mortier [17], employing equations such as *f_k_*^+^ = [q(N + 1) − q(N)] for nucleophilic attack; *f_k_*^−^ = [q(N) − q(N − 1)] for electrophilic attack and *f_k_*^0^ = ½ [q(N + 1) − q(N − 1)] for radical attack, where N is the total numbers of electrons. When a molecule accepts electrons, the electrons tend to go to places were *f_k_*^+^ is large because it is at these locations that the molecule is most able to stabilize additional electrons. Therefore a molecule is susceptible of an electrophilic attack at sites where *f_k_*^−^ is large. The calculated values of the Fukui function (*f*_k_^−^) are shown in Figure 1. 

**Scheme 2:**
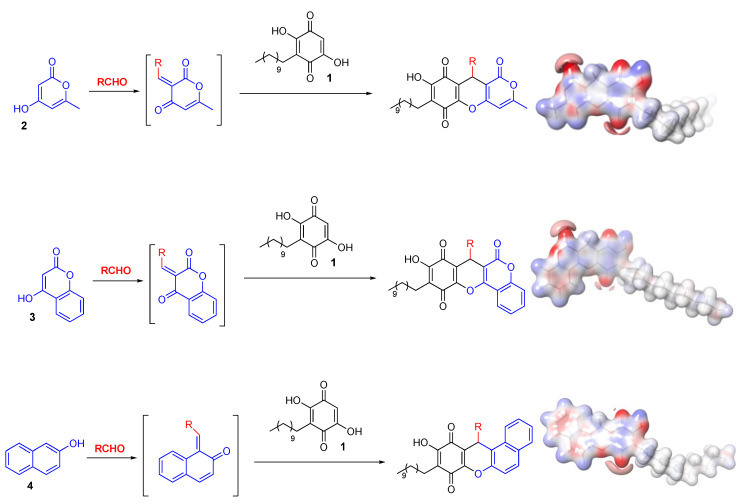
Formation of embelin-conjugates.

As we can see, compounds **2**–**4** show higher values of (f^−^) than embelin (**1**), which implies that the quinone methide intermediate is presumably formed from these compounds, and next the nucleophilic attack of embelin will take place on the more electrophilic α, β-unsaturated carbonyl, followed of intramolecular cyclization with loss of H_2_O to yield the corresponding conjugates (Scheme 2).

Furthermore, for assessing the molecular diversity of the devised molecular framework, electrostatic polar surface area of energy-minimized conformers as well as the isosurface diagram of each adduct (R=H) were obtained by the calculation of electrostatic polar potentials and electron density [18]. As shown in Scheme 2, these three conjugates have a distinguishable display of electrostatic polar surface area because of the differentiation in electronic properties of each privileged substructure. The further expansion of molecular diversity can be achieved via the introduction of various moieties at the dihydropyran such as aliphatic and aromatic groups with electron donating and electron withdrawing substituents.

First, we decided to study the multicomponent reaction of embelin, 4-hydroxy-6-methyl-2*H*-pyran-2-one (**2**) and 4-bromobenzaldehyde. We used different reaction conditions and several catalysts employed in multicomponent reactions of 1,3-dicarbonyl compounds such as EDDA [19], PTSA [20], Sc(OTf)_3_ [21], Yb(OTf)_3_ [22], and InCl_3_ [23]. Some results are shown in Table 1.

The use of ethylendiamine diacetate (EDDA) as an effective organocatalyst for the initial Knoevenagel condensation did not produce the desired adduct **3a** (entries 1–3). When InCl_3_ (10 mol%) was used in EtOH under reflux compound **3a** was obtained in low yield (32%, entry 4). The yield was improved when the reaction was carried out without solvent (52%, entry 5) at 120 °C. The use of other Lewis acids (entries 6 and 7) and *p*-toluenesulfonic acid (PTSA) (entry 8) under neat conditions at 120 °C, did not improved the yields. Increasing the load of InCl_3_ (20 mol%) gave higher yield (58%). We also carried out the multicomponent reaction without catalyst (entry 9) and adduct **3a** was achieved in low yield (11%). Thus, we selected the reaction conditions of entry 10 and the scope of this multicomponent process was then assessed through the variation of diverse aromatic and aliphatic aldehydes (Table 2). Diversely substituted tricyclic embelin adducts (**3a**–**3l**) could be prepared in moderated yields, demonstrating the versatility of this domino process. As a general trend, the multicomponent reaction is tolerant to a large variety of aryl-substituted aldehydes with electron-donating and electron-withdrawing groups, and also the reaction proceeds with aliphatic aldehydes.

**Scheme 3:**
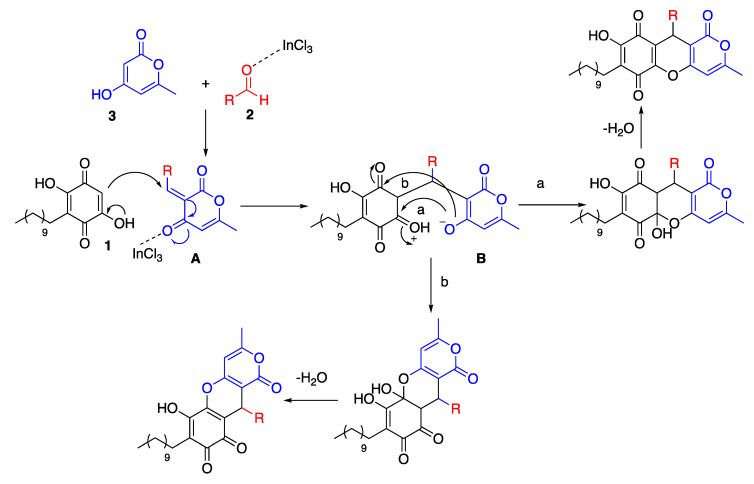
Plausible formation of adducts **3a–3l.**

The reaction can be rationalized via the formation of a conjugated electron-deficient enone (**A**) through a Knoevenagel condensation of **2** and an aldehyde. The next step of this mechanism could involve a Michael addition of embelin (**1**) to the reactive quinone methide intermediate to yield the intermediate (**B**), which can undergo an intramolecular cyclization through carbonyl **a** to give the *para*-quinone adduct or through carbonyl **b** to yield the *ortho*-quinone adduct (Scheme 3). 

The process is regioselective since only the 1,4-benzoquinone adduct is obtained. A plausible explanation for this regioselectivity is that the reaction takes place through a more electron deficient carbonyl moiety next to another carbonyl group. Two new fused rings next to the benzoquinone core and three σ bonds (two C-C σ bonds and one C-O σ bond) were formed in this multicomponent reaction. The regiosubstitution of the corresponding adducts was confirmed by the three-bond correlations detected in the HMBC spectrum and also by the ^13^C NMR values of the quinone carbonyls [10,11,12] (Supplementary Materials). The InCl_3_ would promote the generation of the key quinone methide through dehydration of the alcohol formed in the Knoevenagel condensation and furthermore it could activate the quinone methide intermediates A.

Next, we decided to synthesize more complex embelin adducts by reacting embelin (**1**), aldehydes and the privileged structure 4-hydroxycoumarin (**4**). These tetracyclic adducts (**4a**–**4l**) compared to adducts **3a**–**3l** present an extension of the structure by the introduction of an aromatic ring fused to the 2*H*-pyran-2-one nucleus, which results very attractive for the establishment of structure–activity relationships after biological evaluation. The same reaction conditions for the synthesis of adducts **3a**–**3l** were used. Table 3 shows the structures and the yields of the obtained conjugates (**4a**–**4l**). As we can see improved yields were achieved by using 4-hydroxycoumarin as nucleophile component in the initial Knoevenagel condensation.

The last series was synthesized using 2-naphthol as nucleophilic component, in this case the tetracyclic adducts do not present the lactone ring of the previous series and they were obtained with higher yields than those from 4-hydroxy-2*H*-pyran-2-one and 4-hydroxycoumarin (Table 4). The conjugates synthesized from the aliphatic aldehydes (**5j**–**5l**) were achieved with the lowest yields.

Since, the conjugation of embelin with other anti-bacterial moieties could provide new candidates with great potency against both drug sensitive and drug-resistant Gram-positive and Gram-negative organisms, all synthesized conjugates were tested for antimicrobial activity. The compounds had no effect on the growth of the assayed Gram-negative bacteria *Escherichia coli* and on the growth of the yeast *Saccharomyces cerevisiae* (MIC > 128 μM). By contrast, many compounds were selectively active against the three Gram-positive bacteria tested: the methicillin-sensitive *Staphylococcus aureus* (MSSA) ATCC25923 strain, the methicillin-resistant *S. aureus* NRS402 strain, which is also intermediate resistant to vancomycin (VISA), and the *Enterococcus faecalis* ATCC29212 strain (Table 5), and they were more active than embelin (**1**). This fact is interesting since bacterial infections, caused by Gram-positive pathogens such as *Staphylococcus* are account for the majority of opportunistic community-acquired and hospital-acquired infections.

As we can see, the less active compounds turned out to be the conjugates with the naphthalene scaffold since most of them have MIC > 128 M (entries 26–37). Thus, the presence of a 2*H*-pyran-2-one moiety seems to be important for the antibacterial activity. In the series from 4-hydroxy-2*H*-pyran-2-one good values were achieved with both aromatic and aliphatic substituents at the dihydropyran ring (entries 2–13). Embelin–coumarin conjugates with aliphatic substituents at the dihydropyran ring were inactive while those with aromatic substituents showed high activity. Regarding the influence of the nature of the substituents on the aromatic ring in the activity, in the series from 4-hydroxy-6-methyl-2*H*-pyran-2-one, halogen substituents in *para* position afforded the lowest MIC values (entries 2–4). In the embelin–coumarin series the best results were obtained with 4-fluorphenyl and 3,4-dimetoxyphenyl groups (entries 4 and 8).

## 3. Materials and Methods 

### 3.1. General Methods

Commercial reagents were purchased from Sigma-Aldrich (Darmstadt, Germany) and Alfa Aesar (Lancashire, UK) and were used without further purification. Analytical thin-layer chromatography was performed on Polygram SIL G/UV254 silica gel plates and chromatograms were visualized under UV light (254 and 360 nm). Pre-coated TLC plates SIL G-100 UV254 (Macherey–Nagel) and SILICA GEL GF plates (1000 μm, Analtech) were used for preparative TLC purification. ^1^H and ^13^C NMR spectra were acquired in CDCl_3_ (0.03% *v*/*v* TMS) DMSO-*d*_6_ or C_6_D_6_ at room temperature using Bruker Avance instruments (Bruker, Billarica, MA, USA) (400 or 500 MHz for ^1^H NMR and 100 or 125 MHz for ^13^C NMR). Chemical shifts are reported in parts per million (ppm). For ^1^H NMR data are reported in the following manner: Chemical shift (integration, multiplicity, coupling constant where applicable). The following abbreviations are used: s (singlet), br (broad), d (doublet), t (triplet), dd (double doublet), td (triplet of doublets), and m (multiplet). Coupling constants (*J*) are given in Hertz (Hz). ^13^C NMR were obtained with complete proton decoupling. MS and HRMS data were recorded in a VG Micromass ZAB-2F spectrometer and an ESI instrument LCT Premier XE Micromass (ESI-TOF). IR spectra were recorded on a Bruker IFS 28/55 spectrophotometer. All compounds were named using the ACD40 Name-Pro program, which is based on IUPAC rules. The embelin (**1**) used in the reactions was obtained from *Oxalis erythrorhiza* Gillies ex Hook. & Arn. following the procedure described in reference [24].

### 3.2. General Procedures for the Multicomponent Reaction between Embelin *(**1**)*, Aldehyde *(**2**)*, and 4-Hydroxy-6-Methyl-2-Pyrone *(**3**)*, with Indium Trichloride as Catalyst

Embelin (1) (20.0 mg, 0.068 mmol), the corresponding aldehyde (**2**) (0.068 mmol), and 4-hydroxy- 6-methyl-2-pyrone (3) (1.0 mmol) were grinded in a mortar for 5 min. Then, 3.1 mg of InCl_3_ (20 mol %) was added and the reaction mixture was grinded again for 15 min, placed in a sealed tube and kept in an oven at 120 °C for 1.5 h. The resulting crude was purified by preparative-TLC chromatography using hexanes: EtOAc (3:2) as eluant.

### 3.3. 10-4(4-bromophenyl)-8-hydroxy-3-methyl-7undecylpyrano[4,3-b]chromene-1,6,9(10H)-trione (3a)

Following the general procedure described above, 22.4 mg (58%) of **3a** were obtained as an amorphous violet solid. ^1^H NMR (400 MHz, C_6_D_6_) δ 0.91 (3H, t, *J* = 6.2 Hz), 1.28 (16H, bs), 1.38 (3H, s), 1.60 (2H, m), 2.55 (2H, t, *J* = 8.9 Hz), 4.78 (1H, s), 5.27 (1H, s), 7.09 (2H, d, *J* = 8.2 Hz), 7.18 (2H, d, *J* = 8.1 Hz); ^13^C NMR (100 MHz, CDCl_3_) δ 14.1 (CH_3_), 20.0 (CH_3_), 22.7 (CH_2_), 28.1 (CH_2_), 29.3 (CH_2_), 29.6 (CH_2_x3), 29.7 (CH_2_ x2), 31.9 (CH_2_), 32.9 (CH), 98.6 (CH), 101.9 (C), 117.5 (C), 120.0 (C), 121.9 (C), 130.3 (CHx2), 131.7 (CHx2), 140.0 (C), 148.1 (C), 158.8 (C), 158.9 (C), 162.2 (C), 162.9 (C), 179.4 (C), 182.2 (C). EIMS *m/z* (%): 568 (M^+^, 0.93), 510 (8), 509 (16), 508 (34), 429 (23), 428 (17), 427 (24), 415 (17), 413 (96), 412 (M^+^-C_6_H_4_Br, 38), 368 (8), 367 (15); HREIMS: 568.1483 (calcd for C_30_H_33_O_6_^79^Br (M^+^) 568.1461); 570.1445 (calcd for C_30_H_33_O_6_^81^Br (M^+^) 568.1441); IR (CHCl_3_) ν_max_ 2923, 1698, 1651, 1626, 1594, 1487, 1383, 1318, 1211, 1173, 1125, 1074, 1037, 993 cm^−1^.

### 3.4. 10-(4-chlorophenyl)-8-hydroxy-3-methyl-7undecylpyrano[4,3-b]chromene-1,6,9(10H)-trione *(**3b**)*

Following the general procedure described above, 17.1 mg (48%) of **3b** were obtained as an amorphous violet solid. ^1^H NMR (400 MHz, C_6_D_6_) δ 0.91 (3H, t, *J* = 5.9 Hz), 1.28 (16H, bs), 1.38 (3H, s), 1.60 (2H, m), 2.55 (2H, t, *J* = 8.0 Hz), 4.80 (1H, s), 5.27 (1H, s), 6.65 (1H, bs), 7.02 (2H, d, *J* = 8.3 Hz), 7.16 (2H, d, *J* = 8.6 Hz); ^13^C NMR (100 MHz, CDCl_3_) δ 14.1 (CH_3_), 20.1 (CH_3_), 22.6 (CH_2_), 22.7 (CH_2_), 28.0 (CH_2_), 29.3 (CH_2_x2), 29.5 (CH_2_), 29.6 (CH_2_x3), 31.9 (CH_2_), 32.8 (CH), 98.4 (CH), 102.0 (C), 117.6 (C), 119.9 (C), 128.8 (CHx2), 130.1 (CHx2), 133.7 (C), 139.5 (C), 147.8 (C), 151.1 (C), 158.6 (C), 161.9 (C), 162.9 (C), 179.4 (C), 181.5 (C); EIMS *m/z* (%): 524 (M^+^, 0.96), 415 (22), 414 (42), 413 (M^+^-C_6_H_4_Cl, 100), 412 (42), 384 (30), 383 (44), 299 (11), 287 (16), 285 (36), 275 (22), 274 (79), 273 (45); HREIMS: 524.1931 (calcd for C_30_H_33_O_6_^35^Cl (M^+^) 524.1966), 526.1940 (calcd for C_30_H_33_O_6_^37^Cl (M^+^) 526.1936); IR (CHCl_3_) ν_max_ 2923, 1698, 1626, 1596, 1318, 1209, 1125, 1092, 1038, 992, 973, 807, 653 cm^−1^.

### 3.5. 10-(4-fluorophenyl)-8-hydroxy-3-methyl-7undecylpyrano[4,3-b]chromene-1,6,9(10H)-trione *(**3c**)*

Following the general procedure described above, 14.5 mg (42%) of **3c** were obtained as an amorphous violet solid. ^1^H NMR (400 MHz, CDCl_3_) δ 0.91 (3H, t, *J* = 5.7 Hz), 1.28 (16H, bs), 1.37 (3H, s), 1.58 (2H, m), 2.54 (2H, t, *J* = 8.4 Hz), 4.84 (1H, s), 5.28 (1H, s), 6.71 (2H, t, *J* = 8.0 Hz), 7.22 (2H, m); ^13^C NMR (100 MHz, CDCl_3_) δ 14.1 (CH_3_), 20.1 (CH_3_), 22.6 (CH_2_), 22.7 (CH_2_), 28.0 (CH_2_), 29.3 (CH_2_x2), 29.5 (CH_2_), 29.6 (CH_2_ x3), 31.9 (CH_2_), 32.6 (CH), 98.4 (CH), 102.2 (C), 115.6 (CHx2, *J*_C-F_ = 21.4 Hz), 117.8 (C), 119.9 (C), 130.3 (CHx2, *J*_C-F_ = 8.13 Hz), 136.8 (C, *J*_C-F_ = 2.3 Hz), 147.7 (C), 151.0 (C), 158.5 (C), 161.9 (C), 162.2 (C, *J*_C-F_ = 245.8 Hz), 162.9 (C), 179.5 (C), 181.5 (C); EIMS *m/z* (%): 508 (M^+^, 100), 415 (5), 414 (M^+^-C_6_H_4_F, 26), 413 (64), 412 (34), 368 15), 367 (35), 314 (27), 313 (12), 285 (26), 275 (12), 274 (53), 272 (26), 271 (25), 257 (15); HREIMS: 508.2251 (calcd. for C_30_H_33_O_6_F (M^+^) 508.2261); IR (CHCl_3_) ν_max_ 2929, 2857, 1701, 1655, 1629, 1598, 1511, 1387, 1322, 1214, 1175, 1128, 1042, 998, 837, 817, 747 cm^−1^.

### 3.6. 10-(3-fluorophenyl)-8-Hydroxy-3-methyl-7undecylpyrano[4,3-b]chromene-1,6,9(10H)-trione *(**3d**)*

Following the general procedure described above, 16.6 mg (48%) of **3d** were obtained as an amorphous violet solid. ^1^H NMR (400 MHz, CDCl_3_) δ: 0.87 (3H, t, *J* = 6.4 Hz), 1.24 (16H, bs), 1.45 (2H, m), 2.26 (3H), 2.44 (2H, m), 4.93 (1H, s), 6.19 (1H, s), 6.92 (1H, m), 7.03 (1H, m), 7.14 (1H, m), 7.25 (1H, m); ^13^C NMR (100 MHz, CDCl_3_) δ 14.1 (CH_3_), 20.1 (CH_3_), 22.6 (CH_2_), 22.7 (CH_2_), 28.0 (CH_2_), 29.3 (CH_2_x2), 29.5 (CH_2_), 29.6 (CH_2_x3), 31.9 (CH_2_), 33.0 (CH), 98.4 (CH), 101.9 (C), 114.8 (CH, *J*_C-F_ = 21.1 Hz), 115.8 (CH, *J*_C-F_ = 21.9 Hz), 124.4 (CH, *J*_C-F_ = 2.3 Hz), 130.1 (CH, *J*_C-F_ = 8.2 Hz), 117.5 (C), 119.9 (C), 143.3 (C, *J*_C-F_ = 5.8 Hz), 147.9 (C), 151.1 (C), 158.7 (C), 161.9 (C), 162.9 (C, *J*_C-F_ = 246.0 Hz), 163.0 (C), 179.4 (C), 181.4 (C); EIMS *m/z* (%): 508 (M^+^, 100), 415 (24), 414 (M^+^-C_6_H_4_F, 33), 413 (82), 369 (12), 368 (31), 367 (39), 285 (14), 274 (26); HREIMS: 508.2236 (calcd for C_30_H_33_O_6_F(M^+^) 508.2261); IR (CHCl_3_) ν_max_ 2926, 2854, 1699, 1625, 1623, 1596, 1446, 1382, 1318, 1206, 1122, 1039, 994, 973, 823 cm^−1^.

### 3.7. 10-(4-nitrophenyl)-8-Hydroxy-3-methyl-7undecylpyrano[4,3-b]chromene-1,6,9(10H)-trione *(**3e**)*

Following the general procedure described above, 14.9 mg (41%) of **3e** were obtained as an amorphous violet solid. ^1^H NMR (400 MHz, C_6_D_6_) δ: 0.91 (3H, t, *J* = 5.6 Hz), 1.27 (16H, bs), 1.40 (3H, s), 1.62 (2H, m), 2.57 (2H, bt, *J* = 9.2 Hz), 4.77 (1H, s), 5.27 (1H, s), 6.69 (1H, s), 7.11 (2H, d, *J* = 7.8 Hz), 7.72 (2H, d, *J* = 7.7 Hz); ^13^C NMR (100 MHz, CDCl_3_) δ 14.1 (CH_3_), 20.2 (CH_3_), 22.7 (CH_2_ x2), 28.0 (CH_2_), 29.3 (CH_2_x2), 29.5 (CH_2_), 29.6 (CH_2_x3), 31.9 (CH_2_), 33.5 (CH), 98.4 (CH), 101.2 (C), 116.8 (C), 120.4 (C), 123.9 (CHx2), 129.9 (CHx2), 147.1 (C), 147.8 (C), 148.2 (C), 151.2 (C), 158.9 (C), 161.7 (C), 163.6 (C), 179.1 (C), 181.4 (C); EIMS *m/z* (%) 535 (M^+^, 100), 415 (15), 414 (29), 413 (M^+^-C_6_H_4_O_2_N, 78), 397 (12), 396 (33), 395 (38), 382 (11), 324 (13), 285 (12), 274 (23), 273 (16), 271 (12); HREIMS: 535.2215 (calcd for C_30_H_33_O_8_N(M^+^) 535.2206); IR (CHCl_3_) ν_max_: 2926, 2855, 2287, 2166, 1719, 1626, 1591, 1518, 1443, 1382, 1343, 1326, 1216, 1171, 1125, 993, 780 cm^−1^.

### 3.8. 10-(3-fluoro-4-methoxyphenyl)-8-Hydroxy-3-methyl-7undecylpyrano[4,3-b]chromene-1,6,9(10H)-trione *(**3f**)*

Following the general procedure described above, 20.5 mg (56 %) of **3f** were obtained as an amorphous violet solid. ^1^H NMR (400 MHz, C_6_D_6_) δ: 0.91 (3H, t, *J*= 5.4 Hz), 1.28 (16H, bs), 1.38 (3H, s), 1.58 (2H, m), 2.54 (2H, t, *J* = 7.8 Hz), 3.17 (3H, s), 4.87 (1H, s), 5.27 (1H, s), 6.41 (1H, t, *J* = 9.5 Hz), 7.16 (1H, s), 7.24 (1H, d, *J* = 10.4 Hz); ^13^C NMR (100 MHz, CDCl_3_) δ 14.1 (CH_3_), 20.1 (CH_3_), 22.6 (CH_2_x2), 28.1 (CH_2_), 29.3 (CH_2_), 29.4 (CH_2_), 29.5 (CH_2_), 29.6 (CH_2_ x3), 31.9 (CH_2_), 32.3 (CH), 56.2 (CH_3_), 98.5 (CH), 102.1 (C), 113.3 (CH, *J*_C-F_ = 0.8 Hz), 116.3 (CH, *J*_C-F_ = 18.7 Hz), 117.5 (C), 119.9 (C), 124.6 (CH, *J*_C-F_ = 8.6 Hz), 133.9 (C), 147.2 (C, *J*_C-F_ = 9.7 Hz), 147.8 (C), 151.2 (C), 153.6 (C), 158.6 (C), 163.2 (C, *J*_C-F_ = 247.6 Hz), 162.8 (C), 179.4 (C), 181.6 (C); EIMS *m/z* (%) 538 (M^+^, 100), 415 (4), 414 (18), 413 (M^+^-C_7_H_6_OF, 21), 412 (14), 397 (33), 287 (17), 285 (18), 274 (30), 271 (13); HREIMS 538.2360 (calcd for C_31_H_35_O_7_F(M^+^) 538.2367); IR (CHCl_3_) ν_max_ 2927, 2856, 1705, 1628, 1600, 1519, 1446, 1386, 1323, 1277, 1216, 1123, 1034, 998, 817 cm^−1^.

### 3.9. 10-(3,4-dimethoxyphenyl)-8-hydroxy-3-methyl-7undecylpyrano[4,3-b]chromene-1,6,9(10H)-trione *(**3g**)*

Following the general procedure described above, 14.2 mg (38 %) of **3g** were obtained as an amorphous violet solid. ^1^H NMR (400 MHz, CDCl_3_) δ 0.91 (3H, t, *J* = 6.3 Hz), 1.28 (16H, bs), 1.39 (3H, s), 1.60 (2H, m), 2.56 (2H, t, *J* = 2.8 Hz), 3.30 (3H, s), 3.47 (3H, s), 4.96 (1H, s), 5.34 (1H, s), 6.48 (1H, d, *J* = 8.2 Hz), 6.80 (1H, dd, *J* = 8.1, 1.3 Hz), 7.27 (1H, s); ^13^C NMR (100 MHz, CDCl_3_) δ 14.1 (CH_3_), 20.1 (CH_3_), 22.6 (CH_2_), 22.7 (CH_2_), 28.0 (CH_2_), 29.3 (CH_2_), 29.4 (CH_2_), 29.5 (CH_2_), 29.6 (CH_2_x3), 31.9 (CH_2_), 32.7 (CH), 55.8 (CH_3_), 56.1 (CH_3_), 98.4 (CH), 102.5 (C), 111.2 (CH), 112.5 (CH), 118.1 (C), 119.7 (C), 120.5 (CH), 133.7 (C), 147.6 (C), 148.7 (C), 148.9 (C), 151.0 (C), 158.3 (C), 162.1 (C), 162.5 (C), 179.7 (C), 181.6 (C); EIMS *m/z* (%): 550 (M^+^, 100), 415 (10), 414 (27), 413 (M^+^-C_8_H_9_O_2_, 14), 410 (21), 299 (11), 284 (12), 274 (36), 270 (10); HREIMS 550.2582 (calcd for C_32_H_38_O_8_ (M^+^) 550.2567); IR (CHCl_3_) ν_max_ 2925, 2854, 1726, 1654, 1625, 15933, 1514, 1447, 1325, 1267, 1217, 1124, 1027, 993, 823 cm^−1^.

### 3.10. 10-(benzo[d][1,3]dioxo-5-yl)-8-hydroxy-3-methyl-7undecylpyrano[4,3-b]chromene-1,6,9(10H)-trione *(**3h**)*

Following the general procedure described above, 12.3 mg (34 %) of **3h** were obtained as an amorphous violet solid. ^1^H NMR (400 MHz, C_6_D_6_) δ 1.02 (3H, t, *J* = 5.5 Hz), 1.39 (16H, bs), 1.47 (3H, s), 1.67 (2H, m), 2.63 (2H, bt, *J* = 7.4 Hz), 4.96 (1H, s), 5.29 (2H, d, *J* = 11.6 Hz), 5.4 (1H, s), 6.70 (1H, d, *J* = 7.6 Hz), 6.91 (1H, d, *J* = 7.6 Hz); ^13^C NMR (100 MHz, CDCl_3_) δ 14.1 (CH_3_), 20.0 (CH_3_), 22.6 (CH_2_), 22.7 (CH_2_), 28.1 (CH_2_), 29.3 (CH_2_x2), 29.4 (CH_2_), 29.5 (CH_2_), 29.6 (CH_2_x2), 31.9 (CH_2_), 32.8 (CH), 98.5 (CH), 101.2 (CH_2_), 102.4 (C), 108.3 (CH), 109.2 (CH), 122.2 (CH), 117.9 (C), 119.8 (C), 134.9 (C), 147.2 (C), 147.6 (C), 147.9 (C), 151.2 (C), 158.4 (C), 162.0 (C), 162.6 (C), 179.6 (C), 181.8 (C); EIMS *m/z* (%): 534 (M^+^, 100), 415 (18), 414 (41), 413 (M^+^-C_7_H_5_O_2_, 20), 412 (24), 397 (4), 393 (36), 380 (4), 287 (12), 284 (19), 283 (15), 274 (60), 273 (16); HREIMS 534.2295 (calcd for C_31_H_34_O_8_ (M^+^) 534.2254); IR (CHCl_3_) ν_max_ 2924, 2853, 1726, 1624, 1591, 1486, 1442, 1325, 1217, 1125, 1036, 994, 806, 643 cm^−1^.

### 3.11. 8-Hydroxy-3-methyl-10-phenyl-7undecylpyrano[4,3-b]chromene-1,6,9(10H)-trione *(**3i**)*

Following the general procedure described above, 17.6 mg (53 %) of **3i** were obtained as an amorphous violet solid. ^1^H NMR (400 MHz, CDCl_3_) δ: 0.91 (3H, t, *J* = 6.9 Hz), 1.28 (16H, bs), 1.35 (3H, s), 1.56 (2H, m), 2.52 (2H, m), 4.95 (1H, s), 5.28 (1H, s), 6.95 (1H, m), 7.07 (2H, t, *J* = 7.0 Hz), 7.44 (2H, d, *J* = 7.2 Hz); ^13^C NMR (100 MHz, CDCl_3_) δ 14.1 (CH_3_), 20.1 (CH_3_), 22.6 (CH_2_), 22.7 (CH_2_), 28.1 (CH_2_), 29.3 (CH_2_ x2), 29.4 (CH_2_), 29.5 (CH_2_), 29.6 (CH_2_x2), 31.9 (CH_2_), 33.2 (CH), 98.4 (CH), 102.4 (C), 118.0 (C), 119.7 (C), 127.8 (CH), 128.7 (CH), 140.9 (C), 147.8 (C), 151.1 (C), 158.5 (C), 161.9 (C), 162.6 (C), 179.6 (C), 181.6 (C); EIMS *m/z* (%) 490 (M^+^, 100), 415 (13), 414 (24), 413 (M^+^-C_6_H_5_, 74), 412 (13), 351 (17), 350 (27), 349 (10), 285 (17), 274 (27), 273 (20), 271 (13), 270 (14); HREIMS 490.2346 (calcd para C_30_H_34_O_6_ (M^+^) 490.2355); IR (CHCl_3_) ν_max_ 2927, 2856, 1701, 1655, 1628, 1599, 1451, 1386, 1322, 1211, 1175, 1127, 1040, 998, 813, 703 cm^−1^.

### 3.12. 10-Hexyl-8-hydroxy-3-methyl-7undecylpyrano[4,3-b]chromene-1,6,9(10H)-trione *(**3j**)*

Following the general procedure described above, 8.6 mg (20%) of **3j** were obtained as an amorphous violet solid. ^1^H NMR (400 MHz, C_6_D_6_) δ 0.85 (6H, m), 1.01 (2H, m), 1.25 (20H, bs), 1.47 (2H, t, *J* = 7.0 Hz), 1.67 (2H, m), 1.85 (2H, t, *J* = 8.8 Hz), 2.27 (3H, s), 2.46 (2H, t, *J* = 6.9 Hz), 4.02 (1H, t, *J* = 6.1 Hz), 6.09 (1H, s); ^13^C NMR (100 MHz, CDCl_3_) δ 13.9 (CH_3_), 14.1 (CH_3_), 19.9 (CH_3_), 22.6 (CH_2_), 22.7 (CH_2_), 25.1 (CH_2_), 27.4 (CH), 28.0 (CH_2_), 29.2 (CH_2_), 29.3 (CH_2_ x2), 29.4 (CH_2_x2), 29.5 (CH_2_), 29.6 (CH_2_x2), 31.7 (CH_2_), 31.9 (CH_2_), 32.4 (CH_2_), 98.5 (CH), 101.6 (C), 118.2 (C), 119.6 (C), 149.5 (C), 151.5 (C), 160.0 (C), 162.2 (C), 162.5 (C), 179.2 (C), 182.0 (C); EIMS *m/z* (%) 498 (M^+^, 0.10), 415 (15), 414 (31), 413 (M^+^-C_6_H_13_, 100), 385 (6), 287 (4), 274 (11); HREIMS: 498.2990 (calcd. for C_30_H_42_O_6_ (M^+^) 498.2981); IR (CHCl_3_) ν_max_ 2923, 2854, 1720, 1623, 1588, 1443, 1399, 1330, 1221, 1116, 1028, 964, 825, 651 cm^−1^.

### 3.13. 8-Hydroxy-3-methyl-10-propyl-7undecylpyrano[4,3-b]chromene-1,6,9(10H)-trione *(**3k**)*

Following the general procedure described above, 6.2 mg (20%) of **3k** were obtained as an amorphous violet solid. ^1^H NMR (400 MHz, CDCl_3_) δ: 0.87 (6H, t, *J* = 6.0 Hz), 1.25 (16H), 1.47 (2H, m), 1.68 (2H, m), 1.82 (2H, m), 2.27 (3H, s), 2.45 (2H, t, *J* = 6.7 Hz), 4.02 (1H, bs), 6.09 (1H, s); ^13^C NMR (100 MHz, CDCl_3_) δ 13.9 (CH_3_), 14.1 (CH_3_), 18.4 (CH_2_), 20.0 (CH_3_), 22.6 (CH_2_), 22.7 (CH_2_), 27.4 (CH), 28.1 (CH_2_), 29.3 (CH_2_x2), 29.6 (CH_2_x4), 31.9 (CH_2_), 34.7 (CH_2_), 98.5 (CH), 101.7 (C), 118.2 (C), 128.8 (C), 130.9 (C), 149.5 (C), 160.1 (C), 162.2 (C), 162.5 (C), 179.4 (C), 182.4 (C). EIMS *m/z* (%): 456 (M^+^, 0.03), 416 (3), 415 (15), 414 (21), 413 (M^+^-C_3_H_7_, 100), 273 (5). HREIMS 456.2517 (calcd. for C_27_H_36_O_6_ (M^+^) 456.2512); IR (CHCl_3_) ν_max_ 2924, 2854, 1712, 1624, 1592, 1445, 1398, 1330, 1214, 1168, 1114, 1036, 969, 812 cm^−1^.

### 3.14. 10-ethyl-8-Hydroxy-3-methyl-7undecylpyrano[4,3-b]chromene-1,6,9(10H)-trione *(**3l**)*

Following the general procedure described above, 7.5 mg (25%) of **3l** were obtained as an amorphous violet solid. ^1^H NMR (400 MHz, CDCl_3_) δ 0.74 (3H, t, *J* = 7.5 Hz), 0.87 (3H, t, *J* = 6.3 Hz), 1.25 (16H, bs), 1.47 (2H, t, *J* = 8.6 Hz), 1.75 (1H, m), 1.95 (1H, m), 2.27 (3H, s, Me-3), 2.46 (2H, t, *J* = 7.4 Hz), 4.04 (1H, t, *J* = 4.3 Hz), 6.09 (1H, s), 7.13 (1H, s); ^13^C NMR (100 MHz, CDCl_3_) δ 9.0 (CH_3_), 14.1 (CH_3_), 20.0 (CH_3_), 22.6 (CH_2_), 22.7 (CH_2_), 24.8 (CH_2_), 28.1 (CH_2_), 28.2 (CH), 29.3 (CH_2_), 29.4 (CH_2_), 29.5 (CH_2_x2), 29.6 (CH_2_x2), 31.9 (CH_2_), 98.5 (CH), 100.9 (C), 117.6 (C), 119.6 (C), 149.7 (C), 151.0 (C), 160.2 (C), 162.3 (C), 162.5 (C), 179.4 (C), 182.4 (C); EIMS *m/z* (%) 442 (M^+^, 1), 416 (5), 415 (14), 414 (33), 413 (M^+^-C_2_H_5_, 100), 385 (4), 287 (4), 275 (5), 274 (12); HREIMS: 442.2349 (calcd for C_26_H_34_O_6_ (M^+^) 442.2355); IR (CHCl_3_) ν_max_ 2924, 2854, 1721, 1623, 1587, 1446, 1399, 1324, 1260, 1219, 1160, 1111, 1018, 984, 824 cm^−1^.

### 3.15. General Procedures for the Multicomponent Reaction between Embelin *(**1**)*, Aldehyde *(**2**)*, And 4-Hydroxycoumarin *(**4**)*, With Indium Trichloride as Catalyst

Embelin (**1**) (20.0 mg, 0.068 mmol), the corresponding aldehyde (**2**) (0.068 mmol), and 4-hydroxycoumarin (**4**) (1.0 mmol) were grinded in a mortar for 5 min. Then, 3.1 mg of InCl_3_ (20 mol %) was added and the reaction mixture was grinded again for 15 min, placed in a sealed tube and kept in an oven at 120 °C for 1.5 h. The resulting crude was purified by preparative-TLC chromatography using toluene: EtOAc (9:1) as eluant.

### 3.16. 7-(4-bromophenyl)-9-hydroxy-10-undecylchromeno[4,3-b]chromene-6,8,11(7H)-trione *(**4a**)*

Following the general procedure described above, 28.7 mg (70 %) of **4a** were obtained as an amorphous violet solid. ^1^H NMR (400 MHz, C_6_D_6_) δ 1.11 (3H, t, *J* = 4.7 Hz), 1.44 (16H, bs), 1.83 (2H, t, *J* = 7.4 Hz), 2.77 (2H, d, *J* = 4.6 Hz), 5.1 (1H, s), 6.83 (1H, s), 6.99 (1H, t, *J* = 7.5 Hz), 7.04 (2H, d, *J* = 8.2 Hz), 7.10 (1H, t, *J* = 6.9 Hz), 7.25 (2H, d, *J* = 7.8 Hz), 8.17 (2H, d, *J* = 7.5 Hz); ^13^C NMR (100 MHz, CDCl_3_) δ 14.1 (CH_3_), 22.7 (CH_3_x2), 28.1 (CH_2_, C-15), 29.3 (CH_2_x2), 29.4 (CH_2_), 29.5 (CH_2_), 29.7 (CH_2_x2), 31.9 (CH_2_), 33.5 (CH), 104.7 (C), 113.2 (C), 116.9 (CH), 117.5 (C), 120.2 (C), 122.1 (C), 123.4 (CH), 124.9 (CH), 130.7 (CH x2), 131.9 (CHx2), 133.2 (CH), 139.5 (C), 147.6 (C), 151.2 (C), 152.7 (C), 154.4 (C), 160.3 (C), 179.4 (C), 181.7 (C); EIMS *m/z* (%) 606 (M^+^, 100), 604 (M^+^, 90), 466 (21), 464 (28), 451 (11), 448 (35), 321 (35); HREIMS: 606.1441 (calcd for C_33_H_33_O_6_^81^Br (M^+^) 606.1440), 604.1458 (calcd for C_33_H_33_O_6_^79^Br (M^+^) 606.1461); IR (CHCl_3_) ν_max_ 3343, 2923, 2852, 1719, 1639, 1609, 1527, 1490, 1457, 1378, 1332, 1289, 1182 cm^−1^.

### 3.17. 7-(4-chlorophenyl)-9-hydroxy-10-undecylchromeno[4,3-b]chromene-6,8,11(7H)-trione *(**4b**)*

Following the general procedure described above, 19.0 mg (50%) of **4b** were obtained as an amorphous violet solid. ^1^H NMR (400 MHz, C_6_D_6_) δ 1.11 (3H, t, *J* = 5.5 Hz), 1.49 (16H, bs), 1.83 (2H, t, *J* = 8.1 Hz), 2.77 (2H, d, *J* = 4.4 Hz), 5.1 (1H, s), 6.81 (1H, s), 6.99 (1H, t, *J* = 7.1 Hz), 7.04 (1H, d, *J* = 8.0 Hz), 7.10 (1H, t, *J* = 7.6 Hz), 7.19 (2H, d, *J* = 7.8 Hz), 7.32 (2H, d, *J* = 7.9 Hz), 8.17 (1H, d, *J* = 7.8 Hz); ^13^C NMR (150 MHz, (CD_3_)_2_SO) δ 14.4 (CH_3_), 22.5 (CH_2_), 22.8 (CH_2_), 28.5 (CH_2_), 29.2 (CH_2_), 29.5 (CH_2_x2), 29.5 (CH_2_x2), 29.7 (CH_2_), 31.7 (CH_2_), 33.6 (CH), 104.9 (C), 113.7 (C), 115.9 (C), 116.9 (CH), 117.1 (C), 123.1 (CH), 125.4 (CH), 128.6 (CHx2), 131.1 (CHx2), 132.2 (CH), 133.5 (C), 141.4 (C), 148.6 (C), 148.7 (C), 152.4 (C), 154.4 (C), 160.3 (C), 176.4 (C), 184.1 (C); EIMS *m/z* (%) 560 (M^+^, 100), 562 (M^+^, 41), 450 (38), 449 (75), 448 (48), 419 (51), 320 (35), 310 (87); HREIMS 560.1990 (calcd for C_33_H_33_O_6_^35^Cl (M^+^) 560.1966), 562.1977 (calcd for C_33_H_33_O_6_^37^Cl (M^+^) 562.1936); IR (CHCl_3_) ν_max_ 3338, 2925, 2854, 1719, 1640, 1611, 1492, 1458, 1395, 1335, 1291, 1231, 1182, 1046 cm^−1^.

### 3.18. 7-(4-fluorophenyl)-9-hydroxy-10-undecylchromeno[4,3-b]chromene-6,8,11(7H)-trione *(**4c**)*

Following the general procedure described above, 20.3 mg (55 %) of **4c** were obtained as an amorphous violet solid ^1^H NMR (400 MHz, C_6_D_6_) δ 1.11 (3H, t, *J* = 5.4 Hz), 1.48 (16H, bs), 1.82 (2H, t, *J* = 7.1 Hz), 2.77 (2H, d, *J* = 6.6 Hz), 5.13 (1H, s), 6.89 (2H, t, *J* = 8.0 Hz), 7.01 (4H, m, *J* = 8.4, 5.5 Hz), 7.09 (1H, d, *J* = 7.5 Hz), 8.18 (1H, d, *J* = 7.5 Hz);^13^C NMR (100 MHz, CDCl_3_) δ: 14.1 (CH_3_), 22.7 (CH_2_x2), 28.1 (CH_2_), 29.3 (CH_2_), 29.4 (CH_2_), 29.5 (CH_2_), 29.6 (CH_2_), 29.7 (CH_2_x2), 31.9 (CH_2_), 33.2 (CH), 104.9 (C), 113.2 (C), 115.7 (CH, *J*_C-F_ = 21.7 Hz), 116.9 (CH), 117.7 (C), 120.0 (C), 123.2 (CH), 124.8 (CH), 128.9 (C), 130.5 (CH, *J*_C-F_ = 8.3 Hz), 133.0 (CH), 136.6 (C), 147.6 (C), 152.6 (C), 154.3 (C), 160.2 (C), 162.2 (C, *J*_C-F_ = 246.1 Hz), 179.4 (C), 181.7 (C); EIMS *m/z* (%) 544 (M^+^, 68), 449 (81), 404 (30), 307 (39), 309 (100), 337 (39), 295 (11); HREIMS 544.2274 (calcd for C_33_H_33_O_6_F (M^+^) 544.2261); IR (CHCl_3_) ν_max_ 2924, 2853, 1712, 1636, 1609, 1561, 1508, 1458, 1329, 1294, 1230, 1186, 1050 cm^−1^.

### 3.19. 7-(3-fluorophenyl)-9-hydroxy-10-undecylchromeno[4,3-b]chromene-6,8,11(7H)-trione *(**4d**)*

Following the general procedure described above, 18.9 mg (51 %) of **4d** were obtained as an amorphous violet solid. ^1^H NMR (500 MHz, C_6_D_6_) δ: 1.11 (3H, t, *J* = 6.6 Hz), 1.49 (16H, bs), 1.79 (2H, t, *J* = 6.9 Hz), 2.74 (2H, m), 5.16 (1H, s), 6.82 (1H, t, *J* = 7.6 Hz), 6.97 (1H, t, *J* = 7.6 Hz), 7.02 (2H, d, *J* = 8.6 Hz), 7.06 (1H, t, *J* = 8.1 Hz), 7.27 (1H, d, *J* = 7.7 Hz), 7.51 (1H, d, *J* = 8.8 Hz), 8.14 (1H, d, *J* = 7.4 Hz); ^13^C NMR (150 MHz, CDCl_3_) δ14.1 (CH_3_), 22.7 (CH_2_x2), 28.1 (CH_2_), 29.3 (CH_2_x2), 29.4 (CH_2_), 29.5 (CH_2_), 29.6 (CH_2_), 29.7 (CH_2_), 31.9 (CH_2_), 33.6 (CH), 104.5 (C), 113.2 (C), 115.0 (CH, *J*_C-F_ = 20.8 Hz), 115.9 (CH, *J*_C-F_ = 22.1 Hz), 116.8 (CH), 117.4 (C), 120.0 (C), 123.2 (CH), 124.5 (CH, *J*_C-F_ = 3.1 Hz), 124.8 (CH), 130.2 (CH, *J*_C-F_ = 9.0 Hz), 133.1 (CH), 143.0 (C), 147.7 (C), 151.2 (C), 152.6 (C), 154.5 (C), 160.2 (C), 162.9 (C, *J*_C-F_ = 246.1 Hz), 179.3 (C), 181.4 (C); EIMS *m/z* (%) 544 (M^+^, 100), 450 (16), 404 (12), 309 (12), 307 (9), 279 (5); HREIMS 544.2240 (calcd for C_33_H_33_O_6_F (M^+^) 544.2261); IR (CHCl_3_) ν_max_ 2924, 2852, 2288, 2167, 1699, 1638, 1613, 1561, 1492, 1454, 1376, 1329, 1233, 1188 cm^−1^.

### 3.20. 9-hydroxy-7-(4-nitrophenyl)-10-undecylchromeno[4,3-b]chromene-6,8,11(7H)-trione *(**4e**)*

Following the general procedure described above, 24.8 mg (64%) of **4e** were obtained as an amorphous violet solid. ^1^H NMR (400 MHz, C_6_D_6_) δ: 1.11 (3H, t, *J* = 6.1 Hz), 1.48 (16H, bs), 1.84 (2H, t, *J* = 7.0 Hz), 2.79 (2H, t, *J* = 6.3 Hz), 5.06 (1H, s), 6.83 (1H, s), 7.00 (1H, t, *J* = 7.6 Hz), 7.06 (1H, d, *J* = 8.1 Hz), 7.12 (1H, t, *J* = 7.3 Hz), 7.27 (2H, d, *J* = 8.3 Hz), 7.89 (2H, d, *J* = 8.2 Hz), 8.18 (1H, d, *J* = 7.8 Hz); ^13^C NMR (100 MHz, CDCl_3_) δ 14.1 (CH_3_), 22.7 (CH_2_x2), 28.0 (CH_2_), 29.3 (CH_2_x2), 29.5 (CH_2_x2), 29.6 (CH_2_x2), 31.9 (CH_2_), 34.1 (CH), 103.9 (C), 112.9 (C), 116.8 (C), 116.9 (CH), 120.5 (C), 123.3 (CH), 123.9 (CHx2), 125.0 (CH), 129.9 (CHx2), 133.5 (CH), 147.5 (C), 147.6 (C), 147.9 (C), 151.3 (C), 152.8 (C), 154.7 (C), 160.2 (C), 179.1 (C), 181.4 (C); EIMS *m/z* (%) 571 (M^+^, 100), 450 (20), 449 (44), 431 (20), 309 (14), 306 (11); HREIMS 571.2201 (calcd for C_33_H_33_O_8_N(M^+^) 571.2206); IR (CHCl_3_) ν_max_ 2923, 2852, 1718, 1639, 1612, 1557, 1517, 1458, 1345, 1294, 1229, 1184, 1102 cm^−1^.

### 3.21. 7-(3-fluoro-4-methoxyphenyl)-9-hydroxy-10-undecylchromeno[4,3-b]chromene-6,8,11(7H)-trione *(**4f**)*

Following the general procedure described above, 25.3 mg (65%) of **4f** were obtained as an amorphous violet solid. ^1^H NMR (400 MHz, C_6_D_6_) δ 1.11 (3H, t, *J* = 6.1 Hz), 1.48 (16H, s), 1.81 (2H, t, *J* = 6.6 Hz), 2.75 (2H, m), 3.36 (3H, s), 5.15 (1H, s), 6.57 (1H, t, *J* = 8.6 Hz), 6.85 (1H, s), 6.98 (1H, t, *J* = 7.6 Hz), 7.05 (1H, d, *J* = 8.4 Hz), 7.08 (1H, dd, *J* = 7.4, 1.1 Hz), 7.29 (1H, d, *J* = 7.5 Hz), 7.45 (1H, d, *J* = 10.9 Hz), 8.16 (1H, d, *J* = 7.9 Hz); ^13^C NMR (150 MHz, DMSO-d6) δ: 14.4 (CH_3_), 22.5 (CH_2_), 22.7 (CH_2_), 28.4 (CH_2_), 29.1 (CH_2_), 29.4 (CH_2_x2), 29.5 (CH_2_x2), 29.6 (CH_2_), 31.7 (CH_2_), 33.3 (CH), 56.3 (CH_3_), 104.9 (C), 113.7 (C), 113.9 (C), 116.6 (C), 116.9 (CH), 117.1 (CH), 118.1 (CH, *J*_C-F_= 4.2 Hz), 123.1 (CH), 125.4 (CH), 125.5 (CH), 133.5 (CH), 135.2 (C), 146.7 (C), 147.8 (C), 151.0 (C), 151.5 (C, *J*_C-F_= 242.6 Hz), 152.4 (C), 154.2 (C), 160.3 (C), 177.7 (C), 183.3 (C); EIMS *m/z* (%) 574 (M^+^, 100), 450 (17), 449 (20), 448 (23), 435 (23), 434 (48), 323 (14), 309 (36); HREIMS 574.2330 (calcd for C_34_H_35_O_7_F (M^+^) 574.2367); IR (CHCl_3_) ν_max_ 3344, 2924, 2853, 2482, 1721, 1639, 1612, 1558, 1517, 1456, 1394, 1337, 1283, 1232, 1184, 1123 cm^−1^.

### 3.22. 7-(3,4-dimethoxyphenyl)-9-hydroxy-10-undecylchromeno[4,3-b]chromene-6,8,11(7H)-trione *(**4g**)*

Following the general procedure described above, 27.1 mg (68 %) of **4g** were obtained as an amorphous violet solid. ^1^H NMR (400 MHz, C_6_D_6_) δ 0.91 (3H, t, *J* = 6.6 Hz), 1.28 (16H, bs), 1.64 (2H, m), 2.60 (2H, m), 3.32 (3H, s), 3.45 (3H, s), 5.04 (1H, s), 6.44 (1H, m), 6.74 (1H, m), 6.79 (1H, m), 6.87 (1H, t, *J* = 7.1 Hz), 6.91 (1H, d, *J* = 8.5 Hz), 7.29 (1H, s), 8.02 (1H, d, *J* = 6.8 Hz); ^13^C NMR (100 MHz, CDCl_3_) δ 14.1 (CH_3_, C-24), 22.6 (CH_2_, C-14), 22.7 (CH_2_, C-23), 28.2 (CH_2_, C-15), 29.3 (CH_2_, C-16), 29.4 (CH_2_, C-17), 29.6 (CH_2_, C-18), 29.7 (CH_2_ x4), 31.9 (CH_2_), 33.3 (CH), 55.8 (CH_3_), 56.1 (CH_3_), 105.1 (C), 111.1 (CH), 112.6 (C), 113.3 (C), 116.8 (CH), 120.7 (CH), 123.1 (CH), 124.7 (CH), 128.9 (C), 131.3 (C), 132.8 (CH), 133.5 (CH), 147.4 (C), 148.6 (C), 148.8 (C), 151.4 (C), 152.5 (C), 154.0 (C), 160.5 (C), 179.5 (C), 181.9 (C); EIMS *m/z* (%): 586 (M^+^, 100), 492 (12), 450 (33), 429 (17), 428 (88), 342 (14), 310 (62), 288 (14); HREIMS: 586.2584 (calcd for C_35_H_38_O_8_(M^+^) 586.2567); IR (CHCl_3_) ν_max_ 2922, 2952, 1713, 1631, 1559, 1514, 1455, 1330, 1268, 1230, 1143, 1048, 1025 cm^−1^.

### 3.23. 7-(benzo[d][1,3]dioxo-5-yl)-9-hydroxy-10-undecylchromeno[4,3-b]chromene-6,8,11(7H)-trione *(**4h**)*

Following the general procedure described above, 22.9 mg (59%) of **4h** were obtained as an amorphous violet solid. ^1^H NMR (400 MHz, C_6_D_6_) δ 1.11 (3H, t, *J* = 5.4 Hz), 1.50 (16H, bs), 1.81 (2H, m), 2.75 (2H, d, *J* = 8.7 Hz), 5.13 (1H, s), 5.37 (2H, d, *J* = 8.8 Hz), 5.38 (1H, s), 6.70 (1H, d, *J* = 7.1 Hz), 6.94 (1H, d, *J* = 9.3 Hz), 6.99 (1H, d, *J* = 7.4 Hz), 7.03 (1H, d, *J* = 8.0 Hz), 7.08 (1H, d, *J* = 8.0 Hz), 7.56 (1H, s), 8.17 (1H, d, *J* = 9.2 Hz); ^13^C NMR (150 MHz, DMSO-d6) δ14.4 (CH_3_), 22.6 (CH_2_), 22.7 (CH_2_), 28.3 (CH_2_), 29.1 (CH_2_), 29.4 (CH_2_x2), 29.5 (CH_2_x2), 29.6 (CH_2_), 31.7 (CH_2_), 33.7 (CH), 101.5 (CH_2_), 105.2 (C), 108.4 (CH), 109.9 (CH), 113.7 (C), 117.0 (C), 117.1 (CH), 118.1 (C), 122.6 (CH), 123.0 (CH), 125.4 (CH), 129.2 (C), 131.8 (C), 133.5 (CH), 136.2 (C), 146.8 (C), 147.6 (C), 152.3 (C), 154.1 (C), 160.4 (C), 177.9 (C), 183.0 (C, C-8); EIMS *m/z* (%) 570 (M^+^, 100), 450 (22), 449 (13), 430 (16), 429 (50), 320 (14), 309 (36), 306 (12); HREIMS 570.2260 (calcd for C_34_H_34_O_8_ (M^+^) 570.2254); IR (CHCl_3_) ν_max_ 2921, 2852, 1706, 1635, 1559, 1490, 1442, 1329, 1293, 1229, 1187, 1102, 1040 cm^−1^.

### 3.24. 9-hydroxy-7-phenyl-10-undecylchromeno[4,3-b]chromene-6,8,11(7H)-trione *(**4i**)*

Following the general procedure described above, 22.1 mg (62%) of **4i** were obtained as an amorphous violet solid. ^1^H NMR (400 MHz, C_6_D_6_) δ 0.91 (3H, t, *J* = 5.6 Hz), 1.28 (16H, bs), 1.60 (2H, t, *J* = 7.5 Hz), 2.54 (2H, d, *J* = 6.6 Hz), 5.03 (1H, s), 6.57 (1H, s), 6.80 (2H, t, *J* = 7.1 Hz), 6.88 (1H, d, *J* = 7.9 Hz), 6.95 (1H, t, *J* = 7.2 Hz), 7.04 (2H, t, *J* = 7.9 Hz), 7.42 (2H, d, *J* = 7.0 Hz), 7.98 (1H, d, *J* = 6.7 Hz); ^13^C NMR (100 MHz, CDCl_3_) δ: 14.1 (CH_3_), 22.7 (CH_2_x2), 28.1 (CH_2_, C-22), 29.3 (CH_2_x2), 29.4 (CH_2_), 29.5 (CH_2_), 29.6 (CH_2_), 29.7 (CH_2_), 31.9 (CH_2_), 33.8 (CH), 105.2 (C), 113.3 (C), 116.8 (CH), 117.9 (C), 119.8 (C), 123.2 (CH), 124.7 (CH), 127.9 (CH), 128.7 (CHx4), 132.9 (CH), 140.7 (C), 147.7 (C), 151.2 (C), 152.6 (C), 154.3 (C), 160.2 (C), 179.5 (C), 181.5 (C); EIMS *m/z* (%) 526 (M^+^, 100), 449 (19), 448 (10), 387 (17), 386 (34), 310 (18), 280 (8); HREIMS: 526.2353 (calcd for C_33_H_34_O_6_ (M^+^) 526.2355); IR (CHCl_3_) ν_max_ 2924, 2853, 1701, 1637, 1614, 1556, 1455, 1375, 1329, 1296, 1231, 1186, 1099, 1048 cm^−1^.

### 3.25. 7-hexyl-9-hydroxy-10-undecylchromeno[4,3-b]chromene-6,8,11(7H)-trione *(**4j**)*

Following the general procedure described above, 24.7 mg (68%) of **4j** were obtained as an amorphous violet solid. ^1^H NMR (400 MHz, CD_3_OD) δ: 0.79 (3H, t, *J* = 5.6 Hz), 0.88 (3H, t, *J* = 6.2 Hz), 1.24 (26H, bs), 1.80 (2H, m), 2.40 (2H, m), 4.0 (1H, s), 7.38 (1H, d, *J* = 7.9 Hz), 7.45 (1H, t, *J* = 7.6 Hz), 7.68 (1H, t, *J* = 7.9 Hz), 8.19 (1H, d, *J* = 7.1 Hz); ^13^C NMR (100 MHz, CDCl_3_) δ 14.0 (CH_3_), 14.1 (CH_3_), 22.6 (CH_2_), 22.7 (CH_2_), 25.1 (CH_2_), 28.1 (CH_2_), 29.2 (CH_2_), 29.3 (CH_2_), 29.4 (CH_2_), 29.5 (CH_2_), 29.6 (CH_2_x2), 29.7 (CH_2_), 31.7 (CH_2_), 31.9 (CH_2_), 32.7 (CH), 104.6 (C), 113.5 (C), 116.8 (CH), 117.9 (C), 119.7 (C), 123.1 (CH), 124.6 (CH), 132.8 (CH), 149.4 (C), 151.3 (C), 152.5 (C), 155.8 (C), 160.9 (C), 179.3 (C), 182.0 (C); EIMS *m/z* (%) 534 (M^+^, 0.10), 451 (26), 449 (M^+^-C_6_H_13_, 100), 323 (4), 310 (16), 308 (8); HREIMS 534.2932 (calcd for C_33_H_42_O_6_(M^+^) 534.2981); IR (CHCl_3_) ν_max_ 3350, 2925, 2855, 1720, 1641, 1611, 1555, 1458, 1390, 1346, 1288, 1235, 1185 cm^−1^.

### 3.26. 9-hydroxy-7-propyl-10-undecylchromeno[4,3-b]chromene-6,8,11(7H)-trione *(**4k**)*

Following the general procedure described above, 12.0 mg (37%) of **4k** were obtained as an amorphous violet solid. ^1^H NMR (400 MHz, CDCl_3_) δ: 0.87 (6H), 1.25 (18H, bs), 1.44 (2H, m), 1.77 (2H, t, *J* = 16.6 Hz), 2.41 (2H, m), 4.01 (1H, s), 7.40 (1H, d, *J* = 8.4 Hz), 7.45 (1H, t, *J* = 8.0 Hz), 7.69 (1H, t, *J* = 8.1 Hz), 8.19 (1H, d, *J* = 7.0 Hz); EIMS *m/z* (%) 492 (M^+^, 1), 450 (56), 449 (M^+^-C_3_H_7_, 100), 421 (4), 310 (11); HREIMS 492.2517 (calcd for C_30_H_36_O_6_ (M^+^) 492.2512); IR (CHCl_3_) ν_max_ 2930, 2859, 2491, 1725, 1642, 1612, 1560, 1461, 1394, 1340, 1290, 1243, 1189, 1092 cm^−1^.

### 3.27. 7-ethyl-9-hydroxy-10-undecylchromeno[4,3-b]chromene-6,8,11(7H)-trione *(**4l**)*

Following the general procedure described above, 12.7 mg (39%) of **4l** were obtained as an amorphous violet solid. ^1^H NMR (400 MHz, CDCl_3_) δ 0.78 (3H, t, *J* = 6.9 Hz), 0.87 (3H, t, *J* = 5.8 Hz), 1.25 (16H, bs), 1.51 (2H, t, *J* = 7.4 Hz), 1.82 (1H, m), 2.00 (1H, m), 2.50 (2H, t, *J* = 8.3 Hz), 4.19 (1H, t, *J* = 5.6 Hz), 7.14 (1H, s), 7.38 (2H, t, *J* = 7.3, 7.7 Hz), 7.61 (1H, t, *J* = 7.4 Hz), 8.01 (1H, d, *J* = 7.7 Hz); ^13^C NMR (100 MHz, CDCl_3_) δ 9.1 (CH_3_), 14.1 (CH_3_), 22.6 (CH_2_), 22.7 (CH_2_), 25.1 (CH_2_), 28.1 (CH_2_), 28.9 (CH_2_), 29.3 (CH_2_), 29.4 (CH_2_), 29.5 (CH_2_), 29.6 (CH_2_x2), 29.6 (CH_2_), 31.9 (CH), 103.9 (C), 113.3 (C), 116.7 (CH), 117.5 (C), 119.7 (C), 122.9 (CH), 124.7 (CH), 132.7 (CH), 149.6 (C), 151.2 (C), 152.5 (C), 156.0 (C), 160.9 (C), 179.4 (C), 182.0 (C); EIMS *m/z* (%) 492 (M^+^, 0.04), 450 (33), 449 (M^+^-C_2_H_5_, 100), 421 (5), 310 (11), 309 (8); HREIMS: 478.2361 (calcd for C_29_H_34_O_6_ (M^+^) 478.2355); IR (CHCl_3_) ν_max_ 3352, 2924, 2853, 1721, 1640, 1611, 1458, 1390, 1347, 1288, 1233, 1185, 1114, 1036 cm^−1^.

### 3.28. General Procedures for the Multicomponent Reaction between Embelin *(**1**)*, Aldehyde *(**2**)*, And 2-Naphthol *(**5**)*, with Indium Trichloride as Catalyst

Embelin (**1**) (20.0 mg, 0.068 mmol), the corresponding aldehyde (**2**) (0.068 mmol), and 2-naphthol (**5**) (1.0 mmol) were grinded in a mortar for 5 min. Then, 3.1 mg of InCl_3_ (20 mol %) was added and the reaction mixture was grinded again for 15 min, placed in a sealed tube and kept in an oven at 120 °C for 1.5 h. The resulting crude was purified by preparative-TLC chromatography using toluene: EtOAc (9:1) as eluant.

### 3.29. 12-(4-bromophenyl)-10-hydroxy-9-undecyl-8H-benzo[a]xanthene-8,11(12H)-dione *(**5a**)*

Following the general procedure described above, 26.0 mg (65%) of **5a** were obtained as an amorphous violet solid. ^1^H NMR (400 MHz, C_6_D_6_) δ 1.11 (3H, t, *J* = 6.2 Hz), 1.47 (16H, bs), 1.80 (2H, t, *J* = 6.5 Hz), 2.76 (2H, q, *J* = 6.7 Hz), 5.70 (1H, s), 7.05 (1H, s), 7.19 (2H, d, *J* = 8.5 Hz), 7.23 (2H, d, *J* = 8.5 Hz), 7.30 (2H, t, *J* = 10.8, 7.7 Hz), 7.50 (1H, d, *J* = 9.0 Hz), 7.55 (1H, d, *J* = 8.9 Hz), 7.66 (1H, d, *J* = 8.1 Hz), 7.92 (1H, d, *J* = 8.4 Hz); ^13^C NMR (100 MHz, CDCl_3_) δ 14.0 (CH_3_, C-23), 22.6 (CH_2_), 22.7 (CH_2_), 28.0 (CH_2_), 29.3 (CH_2_), 29.4 (CH_2_), 29.5 (CH_2_), 29.6 (CH_2_x3), 31.9 (CH_2_), 34.8 (CH), 115.1 (C), 115.7 (C), 117.5 (CH), 119.2 (C), 121.2 (C), 123.3 (CH), 125.6 (CH), 127.5 (CH), 128.7 (CH), 130.0 (CH), 130.4 (CHx2), 130.6 (C), 131.8 (CHx2), 132.0 (C), 141.8 (C), 147.5 (C), 149.3 (C), 151.1 (C), 180.5 (C), 182.1 (C); EIMS *m/z* (%) 586 (M^+^, 1), 447 (18), 445 (15), 433 (22), 432 (33), 431 (M^+^-C_6_H_4_Br, 100), 403 (4), 303 (10), 292 (13), 291 (12), 289 (11); HREIMS 588.1680 (calcd for C_34_H_35_O_4_^81^Br (M^+^) 588.1698), 586.1752 (calcd for C_34_H_35_O_4_^79^Br (M^+^) 586.1719); IR (CHCl_3_) ν_max_ 2923, 2852, 1630, 1593, 1516, 1463, 1394, 1327, 1214, 1177, 1115, 1072, 1009, 974, 814 cm^−1^.

### 3.30. 12-(4-chlorophenyl)-10-hydroxy-9-undecyl-8H-benzo[a]xanthene-8,11(12H)-dione *(**5b**)*

Following the general procedure described above, 44.0 mg (81 %) of **5b** were obtained as an amorphous violet solid. ^1^H NMR (400 MHz, C_6_D_6_) δ: 1.11 (3H, t), 1.47 (16H, bs), 1.79 (2H, m), 2.75 (2H, q, *J* = 6.6 Hz), 5.73 (1H, s), 6.99 (1H, s), 7.08 (2H, d, *J* = 8.2 Hz), 7.27 (2H, d, *J* = 8.2 Hz), 7.33 (2H, t, *J* = 7.5 Hz), 7.50 (1H, d, *J* = 8.9 Hz), 7.55 (1H, d, *J* = 8.4 Hz), 7.66 (1H, d, *J* = 8.0 Hz), 7.91 (1H, d, *J* = 8.2 Hz); ^13^C NMR (100 MHz, DMSO-d6) δ 14.0 (CH_3_), 22.6 (CH_2_), 22.7 (CH_2_), 28.0 (CH_2_), 29.3 (CH_2_), 29.4 (CH_2_), 29.5 (CH_2_), 29.6 (CH_2_x3), 31.9 (CH_2_), 34.8 (CH), 115.1 (C), 115.7 (C), 117.5 (CH), 119.2 (C), 121.2 (C), 123.3 (CH), 125.6 (CH), 127.5 (CH), 128.7 (CH), 130.0 (CH), 130.4 (CHx2), 130.6 (C), 131.8 (CHx2), 132.0 (C), 141.8 (C), 147.5 (C), 149.3 (C), 151.1 (C), 180.5 (C), 182.1 (C); EIMS *m/z* (%) 542 (M^+^, 1), 433 (19), 432 (49), 431 (M^+^-C_6_H_4_Cl, 100), 404 (16), 403 (21), 402 (21), 401 (36), 303 (16), 292 (21), 288 (19); HREIMS 544.2135 (calcd for C_34_H_35_O_4_^37^Cl (M^+^) 544.2194), 542.2208 (calcd for C_34_H_35_O_4_^35^Cl (M^+^) 542.2224); IR (CHCl_3_) ν_max_ 2929, 2858, 1635, 1598, 1520, 1493, 1467, 1399, 1330, 1216, 1182, 1093, 977, 825, 745 cm^−1^.

### 3.31. 12-(4-fluorophenyl)-10-hydroxy-9-undecyl-8H-benzo[a]xanthene-8,11(12H)-dione *(**5c**)*

Following the general procedure described above, 21.5 mg (60%) of **5c** were obtained as an amorphous violet solid. ^1^H NMR (400 MHz, C_6_D_6_) δ: 1.11 (3H, t, *J* = 6.0 Hz), 1.47 (16H, bs), 1.78 (2H, t, *J* = 7.2 Hz), 2.74 (2H, q, *J* = 7.4 Hz), 5.77 (1H, s), 6.77 (2H, t, *J* = 8.6 Hz), 6.99 (1H, s), 7.32 (4H, dd, *J* = 8.8, 4.0 Hz), 7.51 (1H, d, *J* = 8.8 Hz), 7.55 (1H, d, *J* = 8.8 Hz), 7.66 (1H, d, *J* = 7.8 Hz), 7.95 (1H, d, *J* = 8.4 Hz); ^13^C NMR (100 MHz, CDCl_3_) δ 14.1 (CH_3_), 22.6 (CH_2_), 22.7 (CH_2_), 28.1 (CH_2_), 29.3 (CH_2_), 29.4 (CH_2_), 29.5 (CH_2_), 29.6 (CH_2_), 29.7 (CH_2_x2), 31.9 (CH_2_), 34.6 (CH), 115.4 (C), 115.6 (CHx2, *J*_C-F_ = 21.4 Hz), 116.0 (C), 117.6 (CH), 119.1 (C), 123.4 (CH), 125.6 (CH), 127.4 (CH), 128.7 (CH), 129.9 (CH), 130.3 (CHx2, *J*_C-F_ = 8.0 Hz), 130.7 (C), 132.0 (C), 138.6 (C), 147.5 (C), 149.2 (C), 151.2 (C), 161.7 (C, *J*_C-F_ = 245.2 Hz), 180.7 (C), 182.2 (C); EIMS *m/z* (%) 526 (M^+^, 1), 508 (34), 433 (13), 432 (41), 431 (M^+^-C_6_H_4_F, 100), 387 (15), 386 (25), 302 (13), 292 (15), 289 (13), 280 (16), 268 (13), 230 (17), 218 (11); HREIMS 526.2542 (calcd for C_34_H_35_O_4_F (M^+^) 526.2519); IR (CHCl_3_) ν_max_ 2928, 2857, 2475, 1621, 1598, 1509, 1467, 1393, 1320, 1228, 1182, 1073, 1048, 975, 834, 818, 746 cm^−1^.

### 3.32. 12-(3-fluorophenyl)-10-hydroxy-9-undecyl-8H-benzo[a]xanthene-8,11(12H)-dione *(**5d**)*

Following the general procedure described above, 22.9 mg (64%) of **5d** were obtained as an amorphous violet solid. ^1^H NMR (400 MHz, C_6_D_6_) δ 1.11 (3H, t, *J* = 7.2 Hz), 1.48 (16H, bs), 1.76 (2H, t, *J* = 7.0 Hz), 2.72 (2H, d, *J* = 8.0 Hz), 5.79 (1H, s), 6.69 (1H, t, *J* = 8.4 Hz), 6.84 (1H, q, *J* = 6.4 Hz), 6.97 (1H, s), 7.13 (1H, d, *J* = 8.2 Hz), 7.28 (2H, t, *J* = 8.1 Hz), 7.48 (1H, d, *J* = 8.7 Hz), 7.53 (2H, d, *J* = 8.8 Hz), 7.64 (1H, d, *J* = 8.0 Hz), 7.93 (1H, d, *J* = 8.1 Hz); ^13^C NMR (100 MHz, CDCl_3_) δ 14.0 (CH_3_), 22.6 (CH_2_), 22.7 (CH_2_), 28.1 (CH_2_), 29.3 (CH_2_), 29.4 (CH_2_), 29.5 (CH_2_), 29.6 (CH_2_), 31.9 (CH_2_), 35.0 (CH), 114. 2 (CH, *J*_C-F_= 20.6 Hz), 115.8 (CH, *J*_C-F_ = 20.9 Hz), 117.6 (CH), 119.2 (C), 123.4 (CH), 124.3 (CH, *J*_C-F_= 2.6 Hz), 125.6 (CH), 127.5 (CH, *J*_C-F_ = 9.4 Hz), 127.8 (C), 128.0 (C), 128.7 (CH), 130.0 (CH), 130.1 (CH), 130.7 (C), 132.0 (C), 145.1 (C, *J*_C-F_ = 5.78 Hz), 147.6 (C), 149.4 (C), 151.2 (C), 162.9 (C, *J*_C-F_= 245.8 Hz), 180.5 (C), 182.0 (C); EIMS *m/z* (%) 526 (M^+^, 1), 434 (12), 433 (26), 432 (78), 431 (M^+^-C_6_H_4_F, 100), 388 (10), 387 (25), 386 (24), 302 (13), 292 (16), 289 (10), 275 (6), 263 (8); HREIMS 526.2496 (calcd for C_34_H_35_O_4_F(M^+^) 526.2519); IR (CHCl_3_) ν_max_ 2925, 2854, 2480, 1616, 1591, 1448, 1323, 1237, 1211, 1124, 1073, 975, 864, 820 cm^−1^.

### 3.33. 10-hydroxy-12-(4-nitrophenyl)-9-undecyl-8H-benzo[a]xanthene-8,11(12H)-dione *(**5e**)*

Following the general procedure described above, 32.7 mg (87 %) of **5e** were obtained as an amorphous violet solid. ^1^H NMR (400 MHz, CDCl_3_) δ 0.87 (3H, t, *J* = 6.3 Hz), 1.24 (16H, bs), 1.47 (2H, m, *J* = 6.9 Hz), 2.46 (2H, t, *J* = 7.3 Hz), 5.93 (1H, s), 7.13 (1H, s), 7.46 (2H, t, *J* = 7.4, 3.6 Hz), 7.53 (2H, d, *J* = 8.4 Hz), 7.58 (1H, d, *J* = 9.0 Hz), 7.76 (1H, d, *J* = 8.0 Hz), 7.85 (1H, d, *J* = 6.7 Hz), 7.89 (1H, d, *J* = 9.0 Hz), 8.08 (2H, d, *J* = 8.4 Hz); ^13^C NMR (100 MHz, CDCl_3_) δ 14.1 (CH_3_), 22.6 (CH_2_), 22.7 (CH_2_), 28.1 (CH_2_), 29.3 (CH_2_), 29.4 (CH_2_), 29.5 (CH_2_), 29.6 (CH_2_x3), 31.9 (CH_2_), 35.4 (CH), 114.3 (C), 114.9 (C), 117.6 (CH), 119.6 (C), 123.1 (CH), 123.9 (CHx2), 125.8 (CH), 127.8 (CH), 128.9 (CH), 129.7 (CHx2), 130.5 (CH), 130.9 (C), 132.1 (C), 146.9 (C), 147.6 (C), 149.7 (C), 151.2 (C), 180.2 (C), 181.9 (C); EIMS *m/z* (%) 553 (M^+^, 100), 432 (27), 431 (M^+^-C_6_H_4_NO_2_, 81), 414 (20), 413(29), 302 (13), 291(15), 290 (12). HREIMS: 553.2459 (calcd. for C_34_H_35_O_6_N(M^+^) 553.2464). IR (CHCl_3_) ν_max_: 3339, 2925, 2854, 1631, 1594, 1516, 1463, 1343, 1213, 1179, 1113, 1072, 976 cm^−1^.

### 3.34. 12-(3-fluoro-4-methoxyphenyl)-10-hydroxy-9-undecyl-8H-benzo[a]xanthene-8,11(12H)-dione *(**5f**)*

Following the general procedure described above, 30.6 mg (81%) of **5f** were obtained as an amorphous violet solid. Twenty milligrams of embelin (0.068 mmol), 9.79 mg (0.068 mmol) of 2-naphthol and 10.58 mg of 3-fluoro-4-methoxybenzaldehyde (0.068 mmol) were grinded for 5 min, then 3.1 mg (20 mol %) of InCl_3_ was added and the reaction mixture was grinded again for 15 min. After 1.5 h at 120 °C, the resulting crude was purified by preparative-TLC using toluene: EtOAc (9:1) as eluant, to provide ^1^H NMR (400 MHz, C_6_D_6_) δ: 1.11 (3H, t, *J* = 6.2 Hz), 1.47 (16H, bs), 1.78 (2H, t, *J* = 6.8 Hz), 2.75 (2H, d, *J* = 6.7 Hz), 3.26 (3H, s), 5.77 (1H, s), 6.39 (1H, t, *J* = 8.5 Hz), 7.01 (1H, d, *J* = 8.2 Hz), 7.31 (2H, m, *J* = 7.8 Hz), 7.51 (1H, d, *J* = 8.8 Hz), 7.55 (1H, d, *J* = 4.8 Hz), 7.58 (1H, dd, *J* = 8.4, 0.9 Hz), 7.67 (1H, d, *J* = 8.0 Hz), 7.99 (1H, d, *J* = 8.3 Hz); ^13^C NMR (100 MHz, CDCl_3_) δ 14.1 (CH_3_), 22.6 (CH_2_), 22.7 (CH_2_), 28.1 (CH_2_), 29.3 (CH_2_), 29.4 (CH_2_), 29.5 (CH_2_), 29.6 (CH_2_x3), 31.9 (CH_2_), 34.3 (CH), 56.1 (CH_3_), 113.2 (C), 115.2 (C), 115.8 (C), 116.5 (CH, *J*_C-F_= 18 Hz), 117.6 (CH), 119.1 (C), 123.4 (CH), 124.3 (CH, *J*_C-F_ = 1.8 Hz), 125.5 (CH), 127.4 (CH, *J*_C-F_ = 4 Hz), 128.7 (CH), 129.9 (CH), 130.7 (CH), 130.9 (C), 132.0 (C), 135.8 (C), 146.6 (C, *J*_C-F_ = 8.8 Hz), 147.5 (C), 149.2 (C), 151.2 (C), 152.2 (C, *J*_C-F_ = 244.7 Hz), 180.7 (C), 182.2 (C); EIMS *m/z* (%) 556 (M^+^, 100), 433 (13), 432 (29), 431 (M^+^-C_7_H_6_O_1_F, 65), 415 (28), 413(29), 304 (19), 302 (16), 291(26), 290 (14), 289 (13), 288 (22), 263 (11); HREIMS 556.2629 (calcd. for C_35_H_37_O_5_F (M^+^) 556.2625). IR (CHCl_3_) ν_max_ 2930, 2858, 1635, 1598, 1518, 1466, 1443, 1331, 1275, 1214, 1122, 1075, 978 cm^−1^.

### 3.35. 12-(3,4-dimethoxyphenyl)-10-hydroxy-9-undecyl-8H-benzo[a]xanthene-8,11(12H)-dione *(**5g**)*

Following the general procedure described above, 32.1 mg (83%) of **5g** were obtained as an amorphous violet solid. ^1^H NMR (400 MHz, CDCl_3_) δ: 0.87 (3H, t), 1.25 (16H, bs), 1.47 (2H, t, *J* = 9.2 Hz), 2.45 (2H, t, *J* = 7.0 Hz), 3.76 (3H, s), 3.79 (3H, s), 5.75 (1H, s), 6.69 (1H, d, *J* = 8.2 Hz), 6.80 (1H, d, *J* = 7.9 Hz), 6.92 (1H, s), 7.19 (1H, s), 7.44 (2H, m), 7.54 (1H, d, *J* = 8.9 Hz), 7.83 (2H, t, *J* = 8.4 Hz), 7.89 (1H, d, *J* = 7.8 Hz); ^13^C NMR (100 MHz, CDCl_3_) δ 14.1 (CH_3_), 22.6 (CH_2_), 22.7 (CH_2_), 28.1 (CH_2_), 29.3 (CH_2_), 29.4 (CH_2_), 29.5 (CH_2_), 29.6 (CH_2_), 29.7 (CH_2_x2), 31.9 (CH_2_), 34.8 (CH), 55.8 (CH_3_), 55.9 (CH_3_), 111.2 (CH), 112.0 (CH), 115.8 (C), 116.3 (C), 117.5 (CH), 119.0 (C), 121.1 (CH), 123.6 (CH), 125.5 (CH), 127.3 (CH), 128.6 (CH), 128.8 (C), 129.7 (CH), 130.9 (C), 131.9 (C), 135.5 (C), 147.6 (C), 148.0 (C), 149.1 (C), 151.2 (C), 180.8 (C), 182.4 (C); EIMS *m/z* (%) 568 (M^+^, 100), 432 (17), 431 (M^+^-C_8_H_9_O_2_, 27), 428 (18), 317 (14), 292 (14), 288 (10); HREIMS: 568.2806 (calcd. for C_36_H_40_O_6_(M^+^) 568.2825). IR (CHCl_3_) ν_max_ 2926, 2854, 1635, 1594, 1513, 1461, 1328, 1266, 1231, 1140, 1072, 1026, 812 cm^−1^.

### 3.36. 12-(benzo[d]dioxo-5-yl)-10-hydroxy-9-undecyl-8H-benzo[a]xanthene-8,11(12H)-dione *(**5h**)*

Following the general procedure described above, 33.0 mg (88%) of **5h** were obtained as an amorphous violet solid. ^1^H NMR (400 MHz, C_6_D_6_) δ 1.11 (3H, t, *J* = 5.9 Hz), 1.47 (16H, bs), 1.77 (2H, m), 2.74 (2H, d, *J* = 4.9 Hz), 5.29 (2H, dd, *J* = 5.9, 4.2 Hz), 5.79 (1H, s), 6.59 (1H, d, *J* = 7.7 Hz), 6.93 (1H, d, *J* = 8.2 Hz), 7.06 (1H, d, *J* = 8.9 Hz), 7.24 (1H, s), 7.31 (1H, d, *J* = 7.8 Hz), 7.50 (2H, m), 7.66 (1H, d, *J* = 7.8 Hz), 8.08 (1H, d, *J* = 8.5 Hz); ^13^C NMR (100 MHz, CDCl_3_) δ 14.1 (CH_3_), 22.6 (CH_2_, C-22), 22.7 (CH_2_, C-13), 28.1 (CH_2_, C-14), 29.3 (CH_2_, C-15), 29.4 (CH_2_, C-16), 29.5 (CH_2_, C-17), 29.6 (CH_2_x2), 29.7 (CH_2_), 31.9 (CH_2_), 34.9 (CH), 101.1 (CH_2_), 108.2 (CH), 109.1 (CH), 115.7 (C), 116.3 (C), 117.6 (CH), 119.1 (C), 122.1 (CH), 123.5 (CH), 125.5 (CH), 127.3 (CH), 128.7 (CH), 129.7 (CH), 130.8 (C), 131.9 (C), 136.8 (C), 146.5 (C), 147.4 (C), 147.9 (C), 149.3 (C), 153.2 (C), 180.8 (C), 182.4 (C); EIMS *m/z* (%) 552 (M^+^, 100), 430 (M^+^-C_7_H_5_O_2_, 10), 413(11), 412 (34), 330 (4), 300(30), 292 (18), 291 (18), 230(14); HREIMS 552.2494 (calcd for C_35_H_36_O_6_ (M^+^) 552.2512); IR (CHCl_3_) ν_max_ 2924, 2853, 1626, 1554, 1486, 1441, 1395, 1329, 1213, 1174, 1116, 1037, 973, 926, 810, 744 cm^−1^.

### 3.37. 10-hydroxy-12-phenyl-9-undecyl-8H-benzo[a]xanthene-8,11(12H)-dione *(**5i**)*

Following the general procedure described above, 28.3 mg (82 %) of **5i** were obtained as an amorphous violet solid. ^1^H NMR (400 MHz, C_6_D_6_) δ 1.11 (3H, t, *J* = 5.5 Hz), 1.48 (16H, bs), 1.76 (2H, t, *J* = 7.2 Hz), 2.71 (2H, d, *J* = 8.7 Hz), 5.87 (1H, s), 7.02 (1H, t, *J* = 6.8 Hz), 7.14 (2H, t, *J* = 7.2 Hz), 7.27 (1H, t, *J* = 7.7 Hz), 7.32 (1H, d, *J* = 7.7 Hz), 7.56 (3H, t, *J* = 7.6 Hz), 7.65 (1H, d, *J* = 7.6 Hz), 8.06 (1H, d, *J* = 8.3 Hz); EIMS *m/z* (%): 508 (M^+^, 0.99), 434 (3), 433 (12), 432 (35), 431 (M^+^-C_6_H_5_, 100), 369 (9), 368 (17), 302 (11), 292 (12). HREIMS: 508.2594 (calcd. para C_34_H_36_O_4_(M^+^) 508.2614). IR (CHCl_3_) ν_max_: 2922, 2851, 1631, 1511, 1385, 1356, 1330, 1218, 1176, 1116, 1078, 975, 751cm^−1^.

### 3.38. 12-hexyl-10-hydroxy-9-undecyl-8H-benzo[a]xanthene-8,11(12H)-dione *(**5j**)*

Following the general procedure described above, 15.8 mg (45 %) of **5j** were obtained as an amorphous violet solid. ^1^H NMR (500 MHz, C_6_D_6_) δ 0.87 (3H, t, *J* = 7.2 Hz), 1.06 (2H, m), 1.11 (3H, t, *J* = 6.6 Hz), 1.24 (2H, m), 1.38 (2H, m), 1.48 (16H, bs), 1.60 (2H, m), 1.87 (2H, t, *J* = 6.9 Hz), 2.01 (2H, m), 2.85 (2H, t, *J* = 8.5 Hz), 4.90 (1H, t, *J* = 4.9 Hz), 7.41 (2H, m), 7.50 (2H, d, *J* = 8.8 Hz), 7.71 (1H, d, *J* = 8.0 Hz), 7.95 (1H, d, *J* = 8.1 Hz); ^13^C NMR (100 MHz, CDCl_3_) δ 13.9 (CH_3_), 14.1 (CH_3_), 22.5 (CH_2_), 22.7 (CH_2_x2), 24.9 (CH_2_t, C-14), 28.2 (CH_2_), 28.6 (CH_2_), 29.2 (CH_2_), 29.3 (CH_2_), 29.4 (CH_2_), 29.6 (CH_2_), 29.7 (CH_2_x3), 31.6 (CH_2_), 34.9 (CH), 115.7 (C), 116.4 (C), 117.4 (CH), 118.9 (C), 122.9 (CH), 125.3 (CH), 127.1 (CH), 128.8 (CHx2), 130.6 (C), 131.9 (C), 148.2 (C), 151.2 (C), 151.7 (C), 180.6 (C), 182.5 (C); EIMS *m/z* (%) 516 (M^+^, 0.05), 433 (16), 432 (41), 431 (M^+^-C_6_H_13_, 100), 334 (27), 317 (19), 292 (12), 291 (13), 277 (27), 263 (20). HREIMS 516.3255 (calcd for C_34_H_44_O_4_(M^+^) 516.3240); IR (CHCl_3_) ν_max_ 3347, 2927, 2857, 1633, 1596, 1521, 1466, 1332, 1276, 1209, 1119, 973, 819, 742 cm^−1^.

### 3.39. 10-hydroxy-12-propyl-9-undecyl-8H-benzo[a]xanthene-8,11(12H)-dione *(**5k**)*

Following the general procedure described above, 15.8 mg (49%) of **5k** were obtained as an amorphous violet solid. ^1^H NMR (400 MHz, CDCl_3_) δ: 0.74 (3H, t, *J*= 7.1 Hz), 0.87 (3H, t, *J* = 6.2 Hz), 0.95 (2H, m), 1.26 (16H, bs), 1.52 (2H, t, *J* = 7.7 Hz), 1.82 (2H, m), 2.50 (2H, t, *J* = 7.2 Hz), 4.83 (1H, t, *J* = 4.8 Hz), 7.41 (1H, d, *J* = 8.9 Hz), 7.49 (1H, t, *J* = 7.5 Hz), 7.59 (1H, t, *J* = 7.2 Hz), 7.76 (1H, d, *J* = 8.9 Hz), 7.85 (1H, d, *J* = 8.0 Hz), 8.05 (1H, d, *J* = 8.4 Hz); ^13^C NMR (100 MHz, CDCl_3_) δ 13.9 (CH_3_), 14.1 (CH_3_), 18.3 (CH_2_), 22.6 (CH_2_), 22.7 (CH_2_), 28.1 (CH_2_), 28.6 (CH_2_), 29.3 (CH_2_), 29.4 (CH_2_), 29.5 (CH_2_), 29.6 (CH_2_x3), 31.9 (CH_2_), 37.2 (CH), 115.8 (C), 116.5 (C), 117.4 (CH), 118.9 (C), 122.9 (CH), 125.4 (CH), 127.1 (CH), 128.8 (CHx2), 130.7 (C), 131.9 (C), 148.2 (C), 151.2 (C), 151.8 (C), 180.6 (C), 182.5 (C); EIMS *m/z* (%) 474 (M^+^, 1), 433 (8), 432 (31), 431 (M^+^-C_3_H_7_, 100), 389 (7), 302 (7), 292 (8); HREIMS 474.2762 (calcd. for C_31_H_38_O_4_(M^+^) 474.2770); IR (CHCl_3_) ν_max_ 2928, 2857, 1635, 1597, 1522, 1465, 1363, 1335, 1276, 1213, 1179, 1118, 1084 cm^−1^.

### 3.40. 12-ethyl-10-hydroxy-9-undecyl-8H-benzo[a]xanthene-8,11(12H)-dione *(**5l**)*

Following the general procedure described above, 10.7 mg (27%) of **5l** were obtained as an amorphous violet solid. ^1^H NMR (400 MHz, CDCl_3_) δ 0.65 (3H, t, *J* = 7.1 Hz), 0.87 (3H, t, *J* = 5.9 Hz), 1.25 (16H, bs), 1.51 (2H, t, *J* = 8.0 Hz), 1.92 (2H, m), 2.50 (2H, t, *J* = 6.9 Hz), 4.85 (1H, t, *J* = 4.0 Hz), 7.41 (1H, d, *J* = 8.8 Hz), 7.48 (1H, t, *J* = 7.4 Hz), 7.58 (1H, d, *J* = 6.4 Hz), 7.77 (1H, d, *J* = 8.8 Hz), 7.85 (1H, d, *J* = 7.9 Hz), 8.04 (1H, d, *J* = 7.9 Hz); ^13^C NMR (100 MHz, CDCl_3_) δ 9.0 (CH_3_), 14.1 (CH_3_), 22.6 (CH_2_), 22.7 (CH_2_), 28.1 (CH_2_), 29.3 (CH_2_), 29.4 (CH_2_x2), 29.5 (CH_2_x2), 29.6 (CH_2_x3), 31.9 (CH), 115.1 (C), 115.8 (C), 117.4 (CH), 118.9 (C), 122.9 (CH), 125.4 (CH), 127.1 (CH), 128.8 (CH), 128.9 (CH), 130.7 (C), 131.9 (C), 148.4 (C), 151.2 (C), 151.9 (C), 180.6 (C), 182.5 (C); EIMS *m/z* (%) 460 (M^+^, 1), 433 (13), 432 (36), 431 (M^+^-C_2_H_5_, 100), 302 (5). HREIMS: 460.2610 (calcd for C_30_H_36_O_4_(M^+^) 460.2614); IR (CHCl_3_) ν_max_ 3379, 2926, 2856, 1618, 1595, 1465, 1324, 1272, 1228, 1119, 1087, 969, 821 cm^−1^.

### 3.41. Biological Assays

Antibacterial activity was determined using the standard broth microdilution method as recommended by the National Committee for Clinical Laboratory Standards [8,11,25]. We used three Gram-positive bacterial strains; methicillin-sensitive *Staphylococcus aureus* ATCC25923 (MSSA), methicillin-resistant vancomycin-intermediate resistant *Staphylococcus aureus* NRS402 (VISA), and *Enterococcus faecalis* ATCC29212; as well as the Gram-negative *Escherichia coli* ATCC35218. Bacterial strains stored at −80 °C were first plated on brain heart infusion (BHI) agar and incubated at 37 °C overnight followed by a second overnight growth in cation-adjusted Mueller–Hinton (MH) broth. Bacterial suspensions were then normalized in fresh MH broth and added to premade 1:2 serial dilutions of each tested compounds and control antibiotics in the same media. The range of concentrations was from 0.5 to 128 (μM for the tested compounds and μg/mL for the reference antibiotics) and the final volume was 200 μL. The expected initial concentration in all wells was 1 × 10^5^ cells/mL. The minimum inhibitory concentration (MIC) was estimated by eye after 24 h of incubation at 37 °C without shaking. 

A similar procedure was used for the yeast *Saccharomyces cerevisiae* BY4741 wild-type strain [26]. In this case, the growing media was YPD and the inoculum was ~2 × 10^4^ cells/mL. The growth was measured at 30 °C after 24 h and 48 h.

### 3.42. Calculation of Electrostatic Polar Potentials, Electron Density, and Fukui Indices

The calculations of Density Functional Theory (DFT) was employed for optimization and minimization of geometry of the compounds shown in Scheme 2, as well as to examine the reactivity of the calculated compounds, their structural and electronic properties were obtained by parameters of reactivity and theoretical properties such as the energy values of highest occupied molecular orbital (HOMO) and lowest unoccupied molecular orbital (LUMO), electronic density and electrostatic potential using the B3LYP functional and the 6-31+G(d,p) basis set implemented in Jaguar-v.10.6 computational program [27,28]. Atomic Fukui indices were derived from Mulliken population of the highest occupied molecular orbital (HOMO) and the LUMO, were used to quantify electrophilicity of a molecule at a particular atomic site. The default convergence criterion implemented in Jaguar was used for self-consistent field (SCF) calculations (accuracy level = quick, convergence criteria: maximum iteration = 48, and energy change = 5 × 10^−5^ hartree) and optimization (maximum steps = 100, convergence criteria = default, initial Hessian = Schlegel guess [29,30].

## 4. Conclusions

In summary, three series of new embelin conjugates were obtained through an InCl_3_ catalyzed three component reaction from embelin (**1**), aldehydes and privileged substructures of antimicrobial interest such as 4-hydroxy-2*H*-pyran-2-one, 4-hydroxy-coumarin, and 2-naphthol. This MCR implies Knoevenagel condensation, Michael addition, intramolecular cyclization, and dehydration. Most part of the conjugates synthesized from 4-hydroxy-2*H*-pyran-2-one and 4-hydroxy-coumarin resulted to be active and selective toward Gram positive bacteria. Some structure–activity relationships were outlined and the most active compounds were **3a**–**3c**, **4c**, **4d,** and **4g** with MICs around 1–2 μM. The present results encourage further research with these compounds in order to develop novel antibiotic agents against Gram-positive bacteria.

## Figures and Tables

**Table 1:** Optimization of the MCR of **1**, **2** and 4-bromobenzaldehyde.

Entry	Conditions	Yield (%) *
1	10 mol% EDDA, DCE, Δ, 24 h	-
2	10 mol% EDDA, EtOH, Δ, 24 h	-
3	30 mol% EDDA, DCE, MW, 120 °C, 30′	-
4	10 mol% InCl_3_, EtOH, Δ, 3 h	32
5	10 mol% InCl_3_, neat, 120 °C, 3 h	52
6	10 mol% Sc(OTf)_3_, neat, 120 °C, 1.5 h	32
7	10 mol% Yb(OTf)_3_, neat, 120 °C, 1.5 h	50
8	10 mol% PTSA, neat, 120 °C, 1.5 h	24
9	neat, 120 °C, 3 h	11
10	20 mol% InCl_3_, neat, 120 °C, 1.5 h	58
11	30 mol % InCl_3,_ neat, 120 °C, 1.5 h	54

* Isolated yields.

**Table 2:** Synthesis of novel tricyclic embelin adducts (**3a**–**3l**).

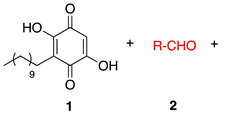	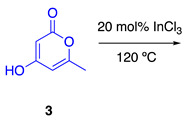	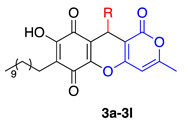
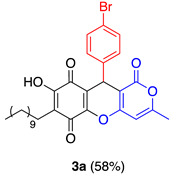	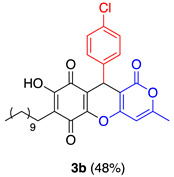	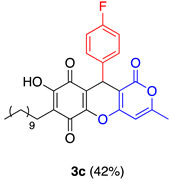
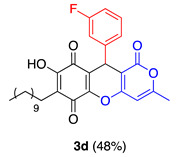	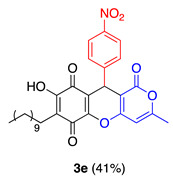	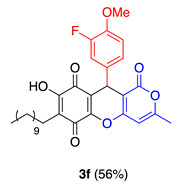
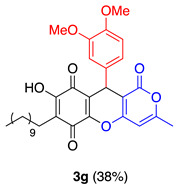	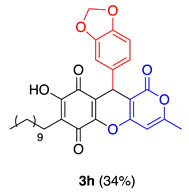	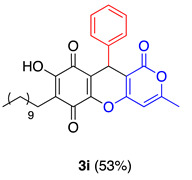
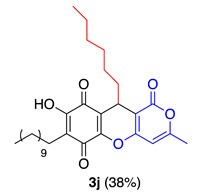	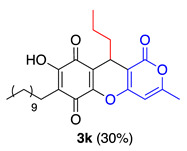	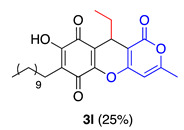

**Table 3:** Synthesis of novel embelin–coumarin conjugates (**4a–4l**).

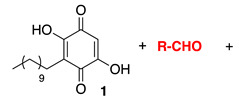	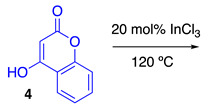	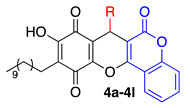
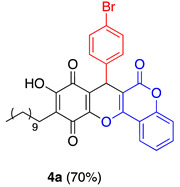	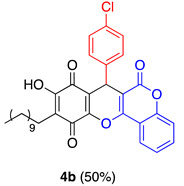	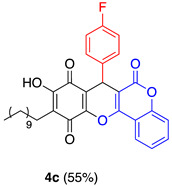
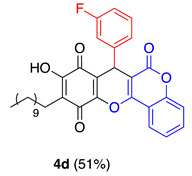	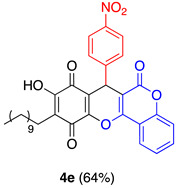	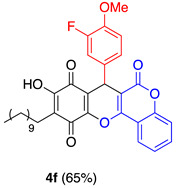
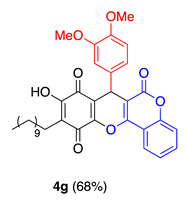	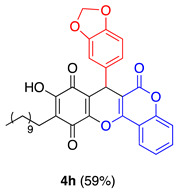	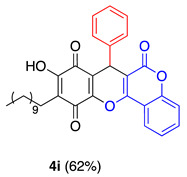
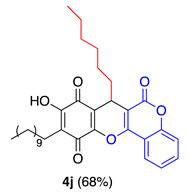	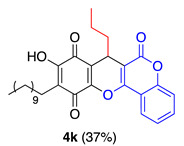	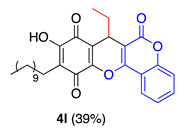

**Table 4:** Synthesis of novel tetracyclic embelin adducts (**5a**–**5l**).

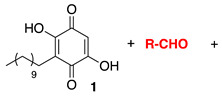	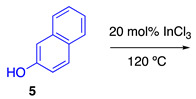	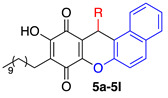
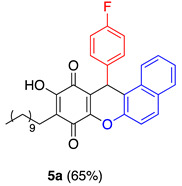	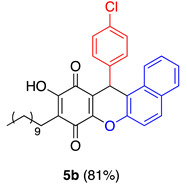	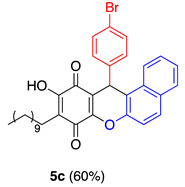
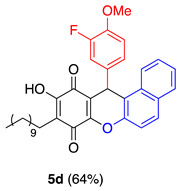	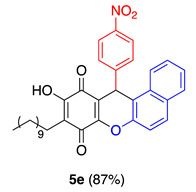	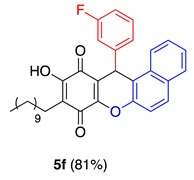
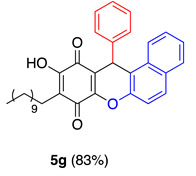	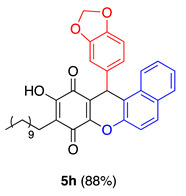	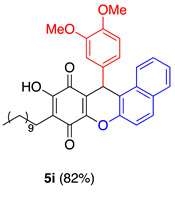
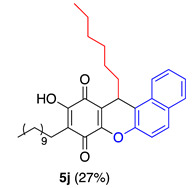	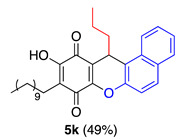	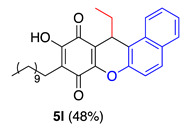

**Table 5:** Minimum inhibitory concentration (MIC) for compounds **1**, **3a**–**l**, **4a**–**l**, and **5a**–**l** against the three selected Gram-positive bacterial strains.

Entry	Compound *	*S. aureus* (ATCC25923)	*S. aureus* (NRS402)	*E. faecalis* (ATCC29212)
1	**1**	32	32	128
2	**3a**	1	<1	2
3	**3b**	2	2	1
4	**3c**	2	2	1
5	**3d**	2	2	2
6	**3e**	4	4	2
7	**3f**	2	2	2
8	**3g**	2	4	4
9	**3h**	4	4	2
10	**3i**	4	4	2
11	**3j**	8	4	2
12	**3k**	4	4	4
13	**3l**	8	8	2
14	**4a**	16	4	8
15	**4b**	2	2	4
16	**4c**	2	2	1
17	**4d**	1	2	2
18	**4e**	2	2	2
19	**4f**	8	2	4
20	**4g**	1	2	2
21	**4h**	2	2	4
22	**4i**	4	2	2
23	**4j**	>128	>128	>128
24	**4k**	>128	>128	>128
25	**4l**	128	64	32
26	**5a**	>128	>128	>128
27	**5b**	>128	>128	>128
28	**5c**	>128	16	32
29	**5d**	>128	32	32
30	**5e**	>128	>128	>128
31	**5f**	>128	>128	>128
32	**5g**	>128	16	16
33	**5h**	>128	>128	>128
34	**5i**	>128	63	32
35	**5j**	>128	>128	>128
36	**5k**	>128	32	32
37	**5l**	>128	32	8
38	ampilicin	<1	>128	8
39	oxacillin	<1	>128	8
40	vancomycin	<1	4	4
41	mupirocin	<1	<1	16

* MICs for tested compounds are in μM and for reference antibiotics are in mg/L.

## Supplementary Materials

The following are available online ^1^H NMR and ^13^C NMR spectra of compounds **3a**–**3l**, **4a**–**4l** and **5a**–**5l**.
