# Supplementary material for: Efficient Multicomponent Synthesis of Diverse Antibacterial Embelin-Privileged Structure Conjugates
Source: Molecules. 2020 Jul 20;25(14):3290. doi: 10.3390/molecules25143290 (PMC7397138; doi:10.3390/molecules25143290)

Supplementary Material

Efficient multicomponent synthesis of diverse antibacterial embelin-privileged structure conjugates

Pedro Martín-Acosta [1], Rosalyn Peña [1], Gabriela Ferensin [2], Alejandro Tapia [2], Isabel Lorenzo-Castrillejo [3], Félix Machín [3,4,5,*], Ángel Amesty [1,*] and Ana Estévez-Braun [1,*]

[1] Instituto Universitario de Bio-Orgánica Antonio González. Departamento de Química Orgánica. Universidad de La Laguna Avda. Astrofísico Fco. Sánchez Nº 2, 38206, La Laguna, Spain; pmartina@ull.edu.es; rosalyn_pf@hotmail.com; aarnesty@ull.edu.es; aestebra@ull.edu.es

[2] Instituto de Biotecnología-Instituto de Ciencias Básicas, Universidad Nacional de San Juan, Av. Libertador General San Martín 1109 (O), CP 5400, San Juan, Argentina; gferesin@unsj.edu.ar; atapia@unsj.edu.ar

[3] Unidad de Investigación Hospital Universitario Nuestra Señora de La Candelaria, 38010, Tenerife, Spain; fmachin@funcanis.es

[4] Instituto de Tecnologías Biomédicas, Universidad de la Laguna, 38200, Tenerife, Spain.

[5] Facultad de Ciencias de la Salud, Universidad Fernando Pessoa Canarias, 35450, Las Palmas de Gran Canaria, Spain.

Contents

^{1}H NMR and ^{13}C NMR of embelin conjugates **3a-3l**, **4a-4l** and **5a-5l**

^{1}H NMR (C_6D_6, 400 MHz) of compound 3a

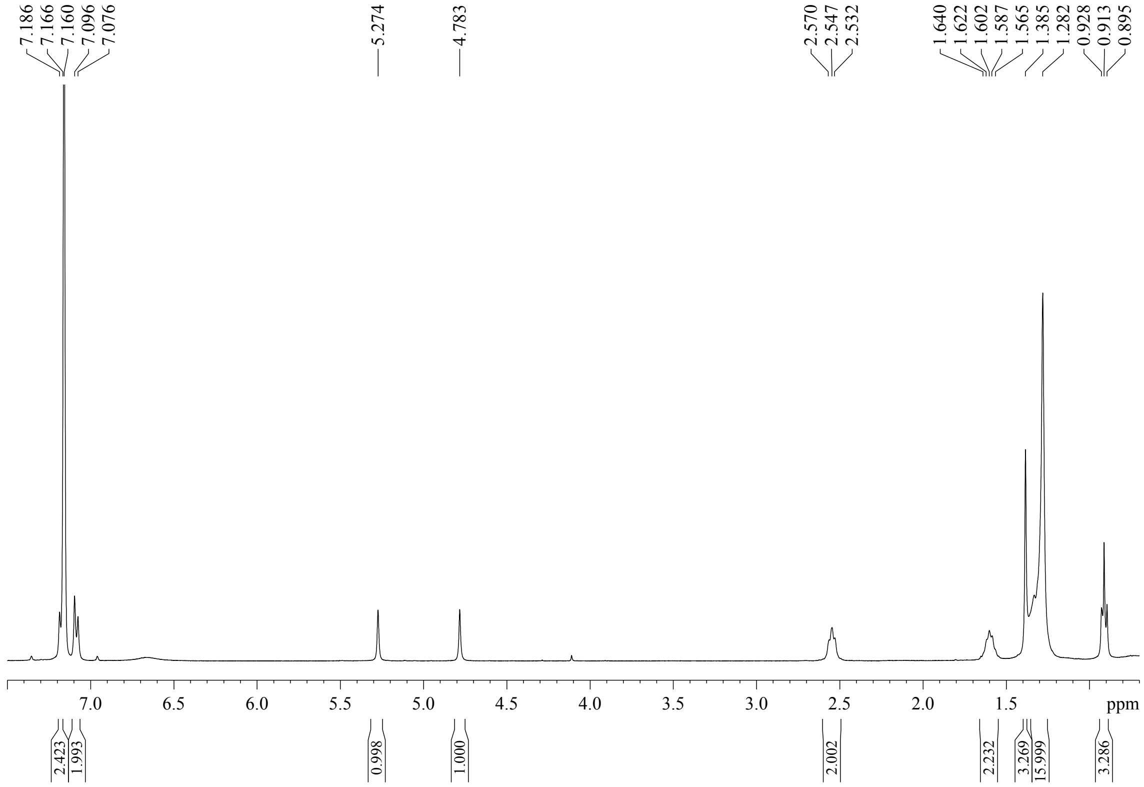

^{13}C NMR ($CDCl_3$, 100 MHz) of compound 3a

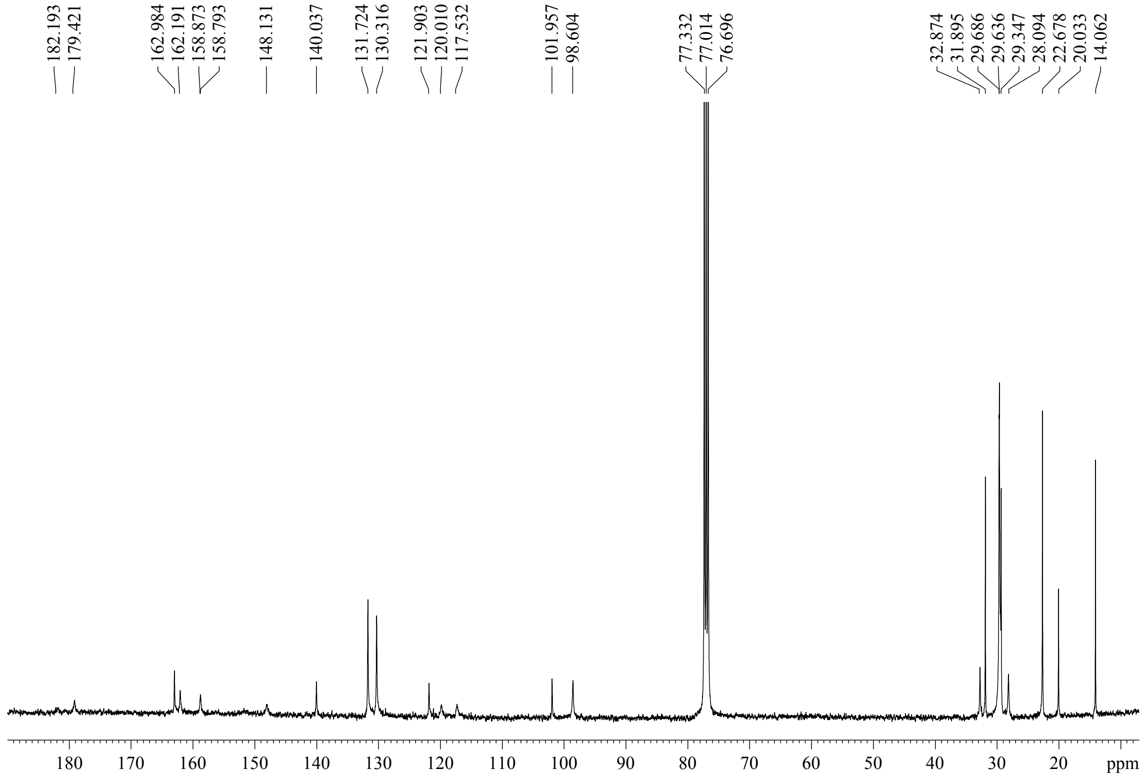

^{1}H NMR (C_6D_6, 400 MHz) of compound 3b

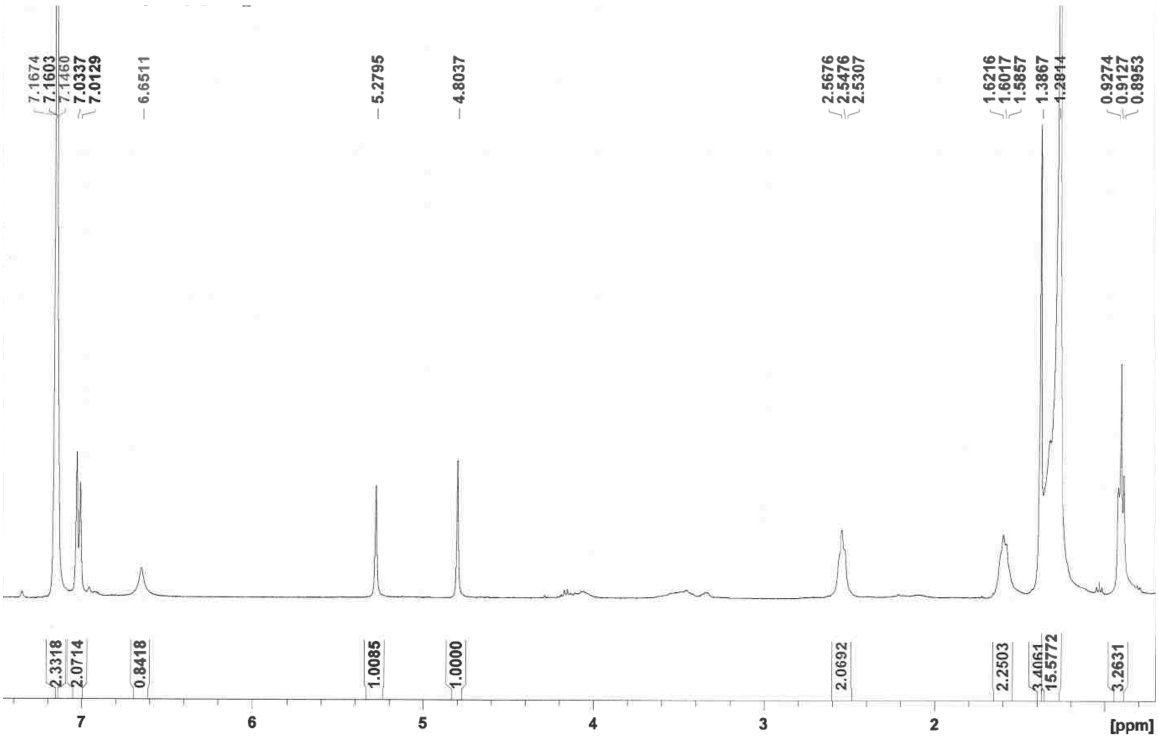

^{13}C NMR ($CDCl_3$, 100 MHz) of compound 3b

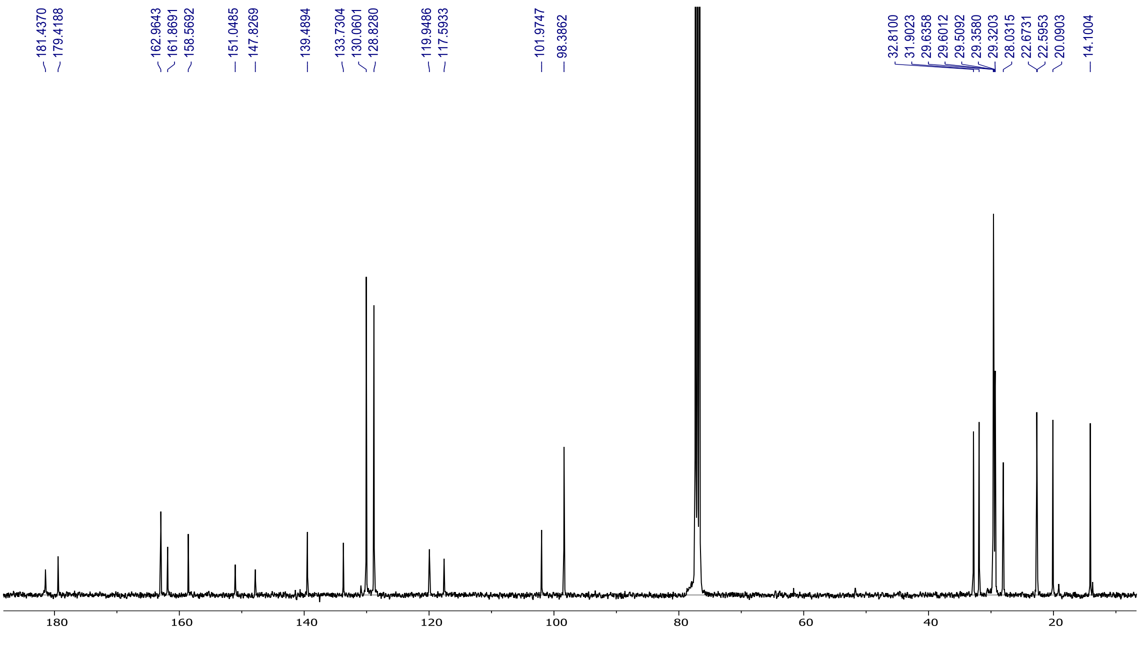

^{1}H NMR ($CDCl_3$, 400 MHz) of compound 3c

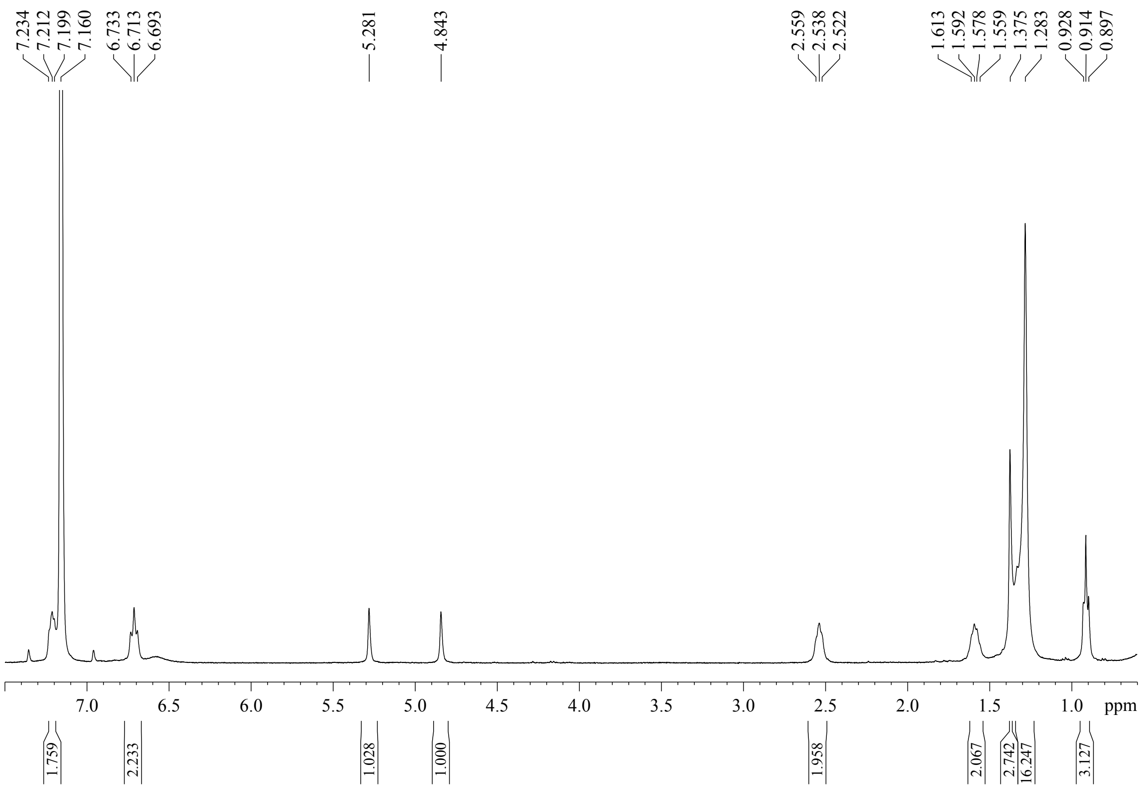

^{13}C NMR ($CDCl_3$, 100 MHz) of compound 3c

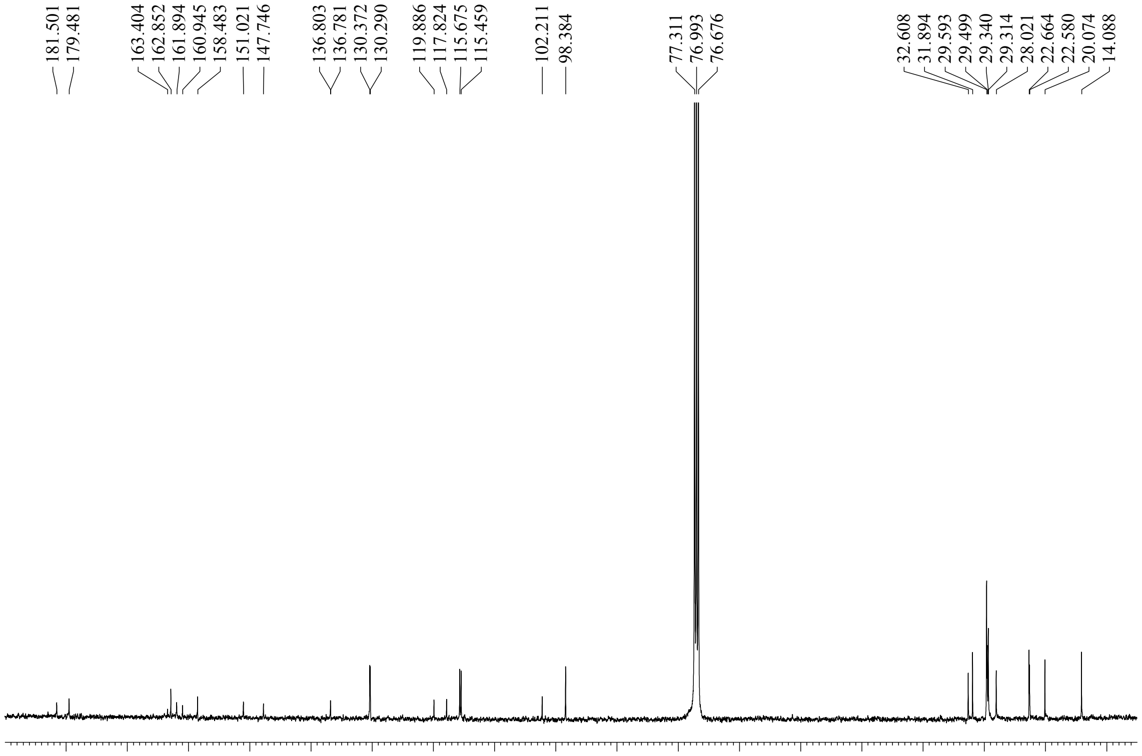

^{1}H NMR ($CDCl_3$, 400 MHz) of compound 3d

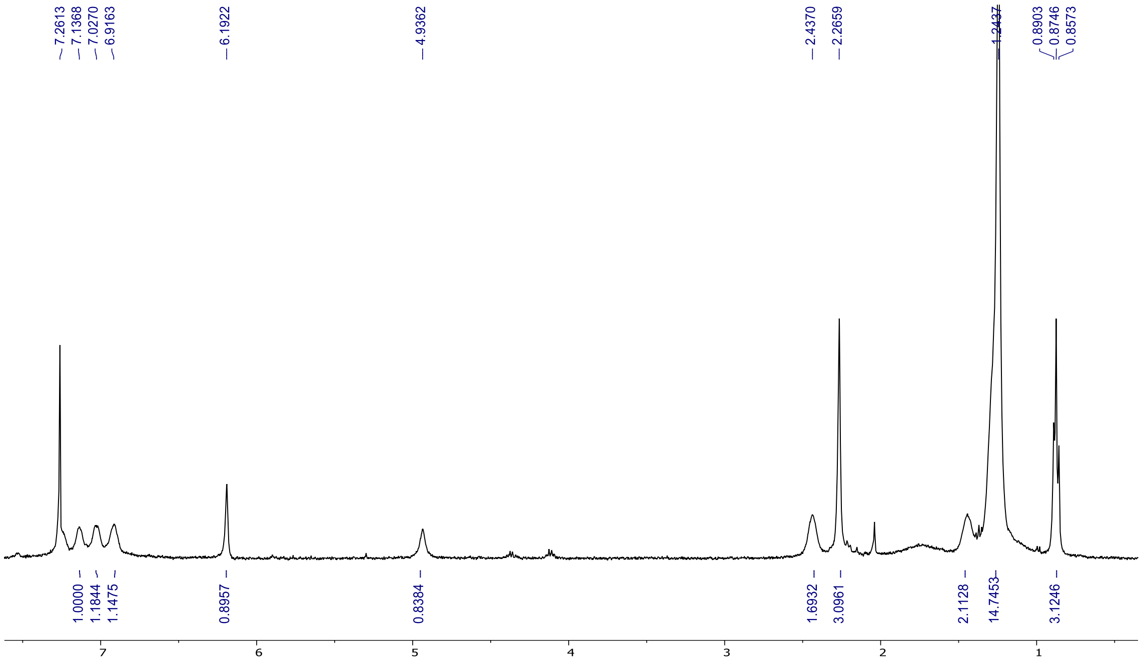

^{13}C NMR ($CDCl_3$, 100 MHz) of compound 3d

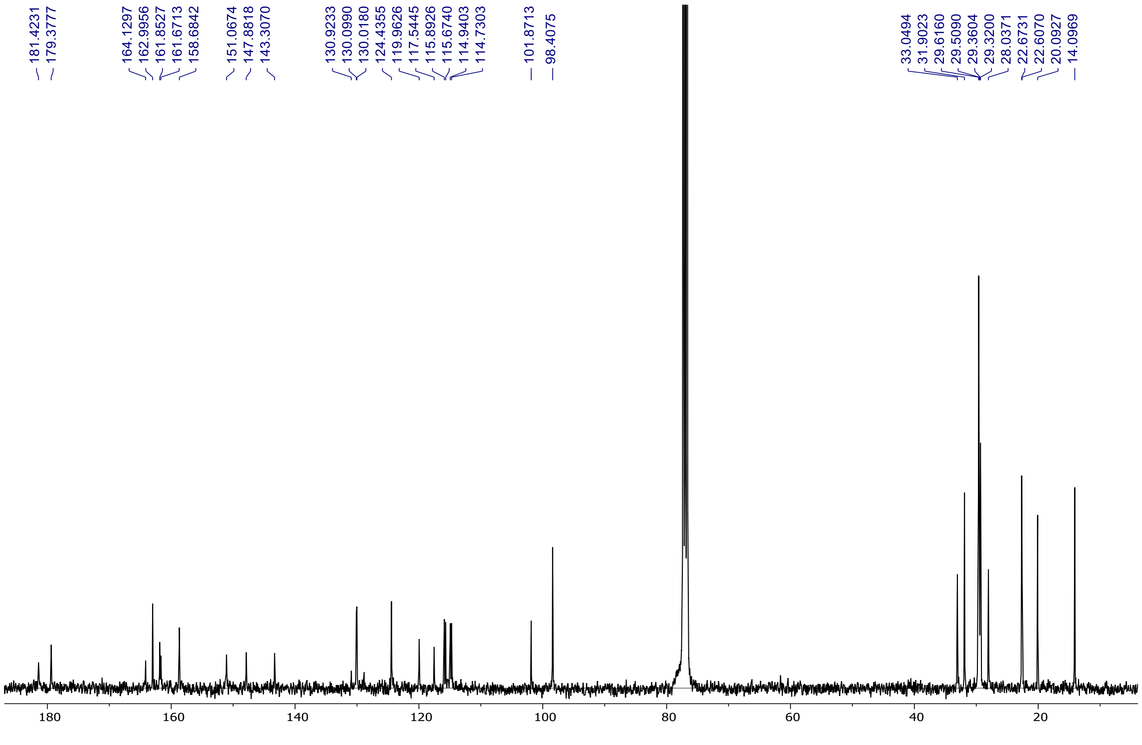

^{1}H NMR (C_6D_6, 400 MHz) of compound 3e

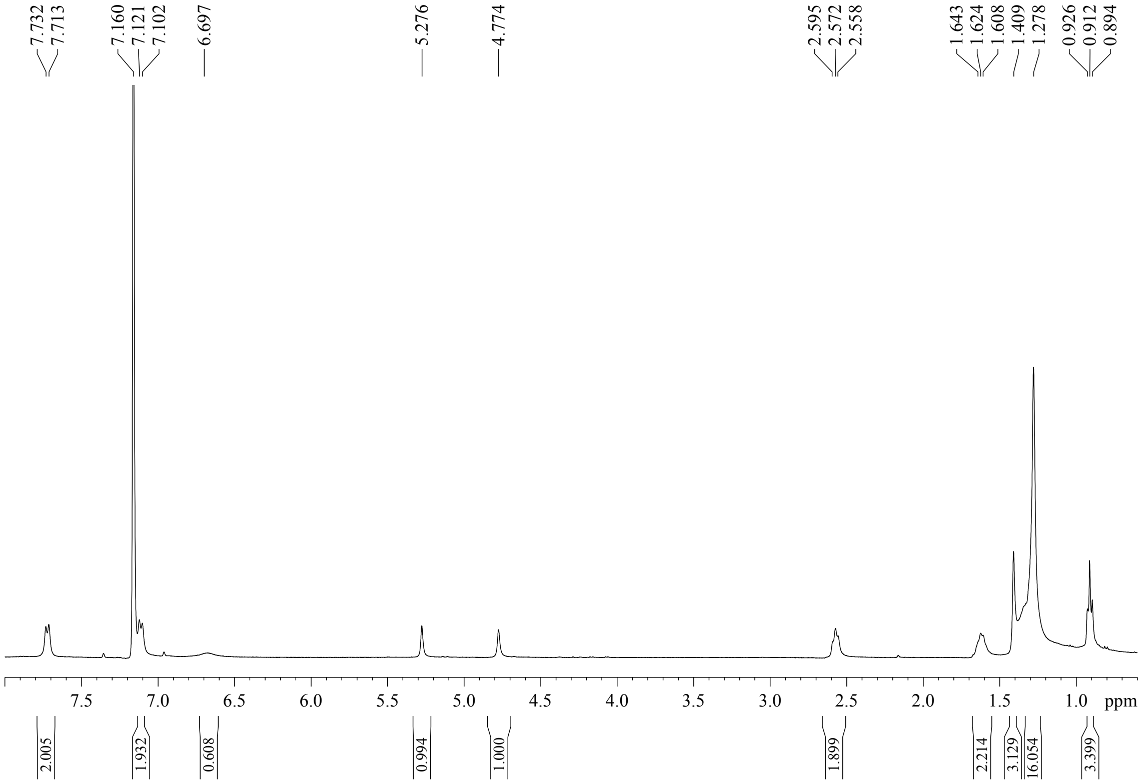

^{13}C NMR ($CDCl_3$, 100 MHz) of compound 3e

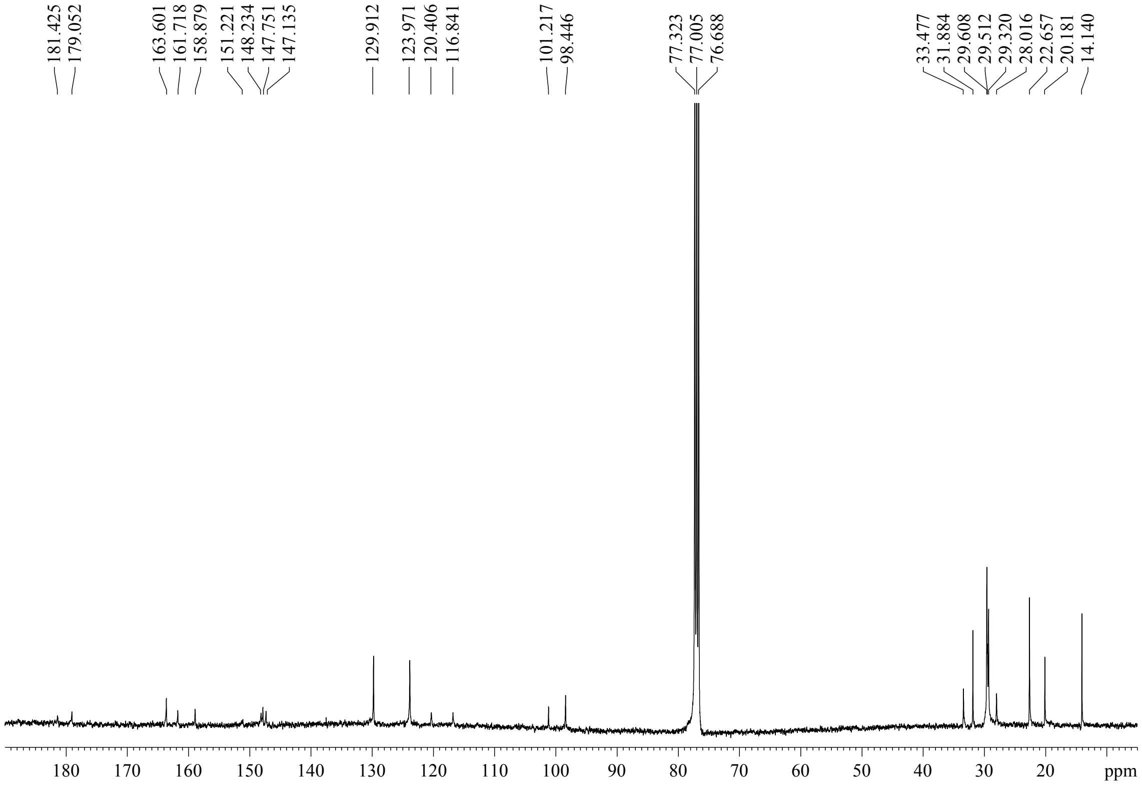

^{1}H NMR ($CDCl_3$, 400 MHz) of compound 3f

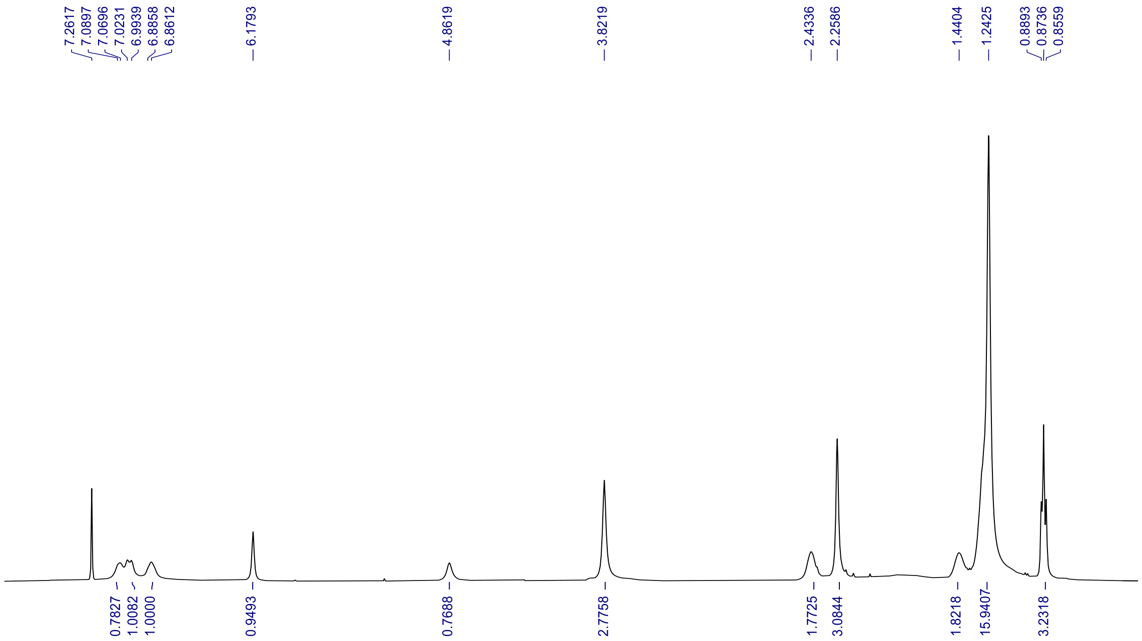

^{13}C NMR ($CDCl_3$, 100 MHz) of compound 3f

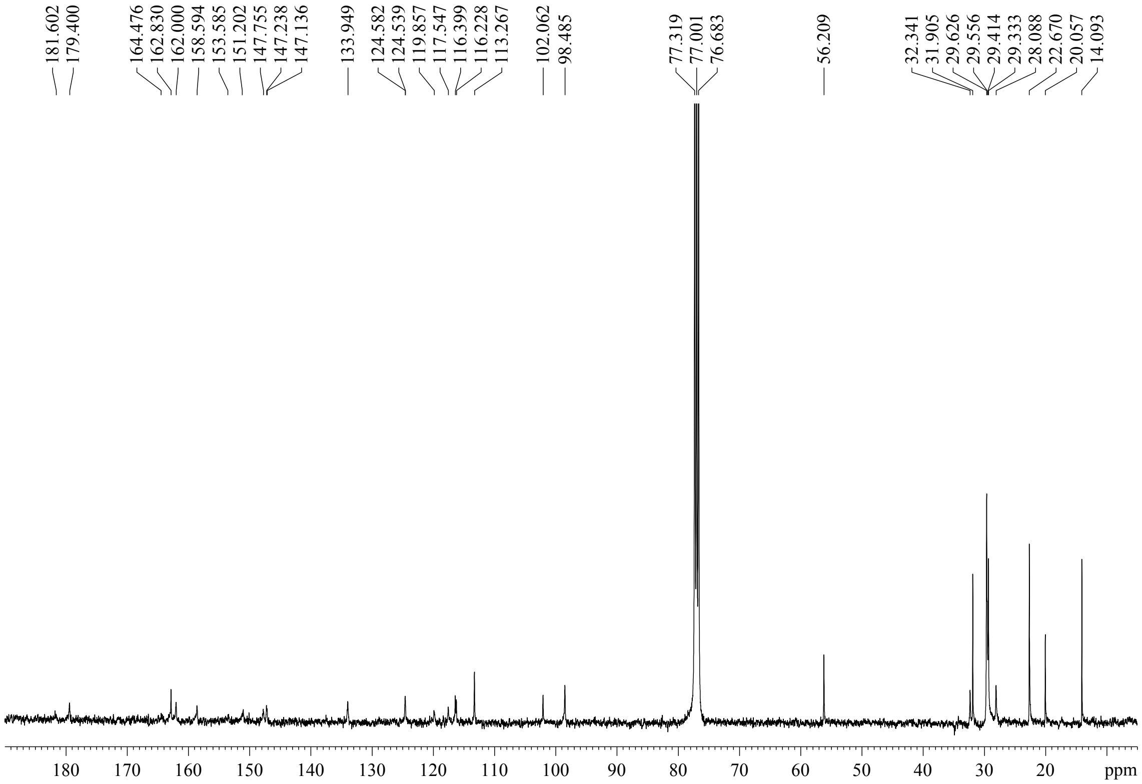

^{1}H NMR ($CDCl_3$, 400 MHz) of compound 3g

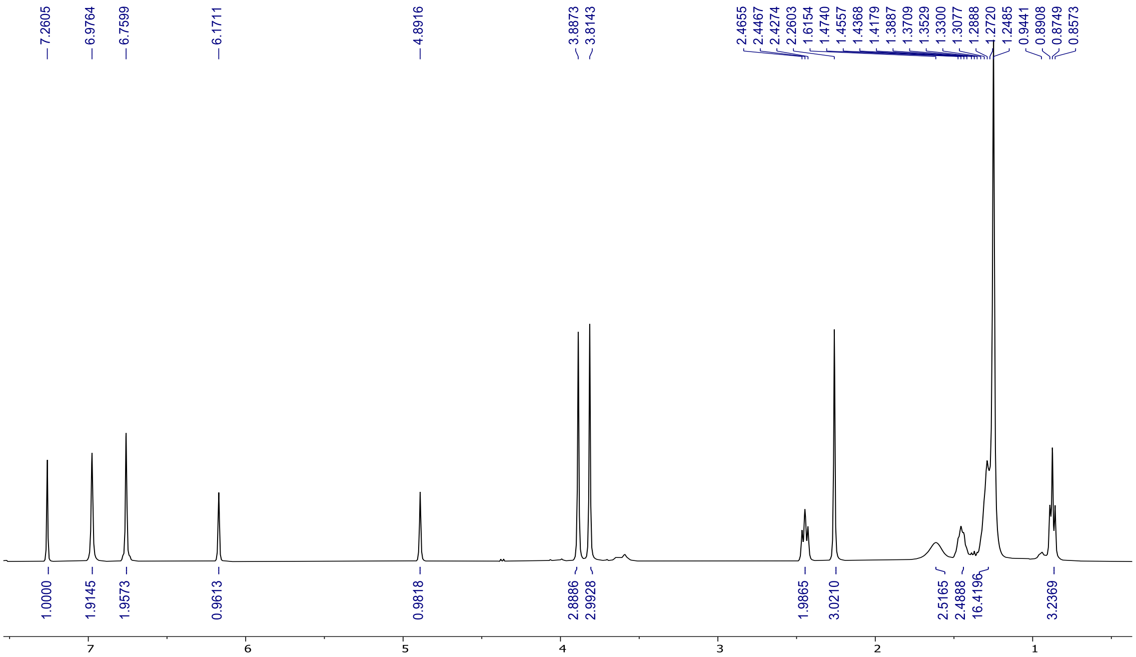

^{13}C NMR ($CDCl_3$, 100 MHz) of compound 3g

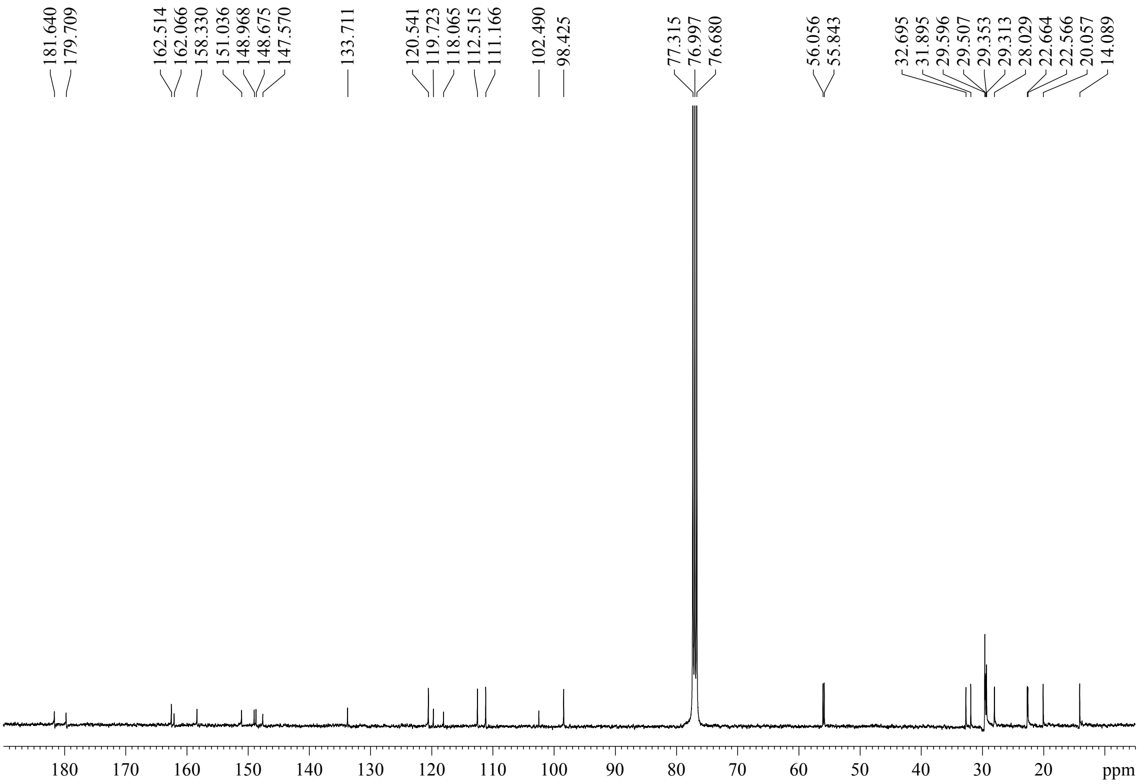

^{1}H NMR (C_6D_6, 400 MHz) of compound 3h

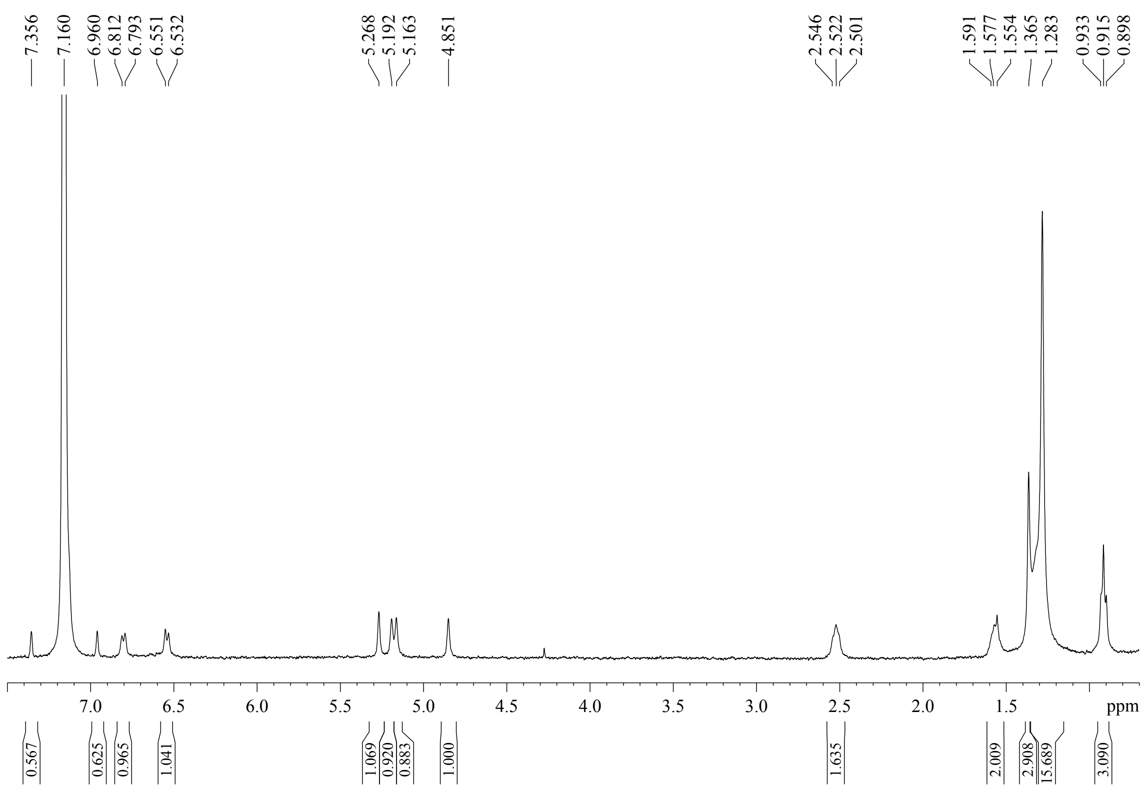

^{13}C NMR ($CDCl_3$, 100 MHz) of compound 3h

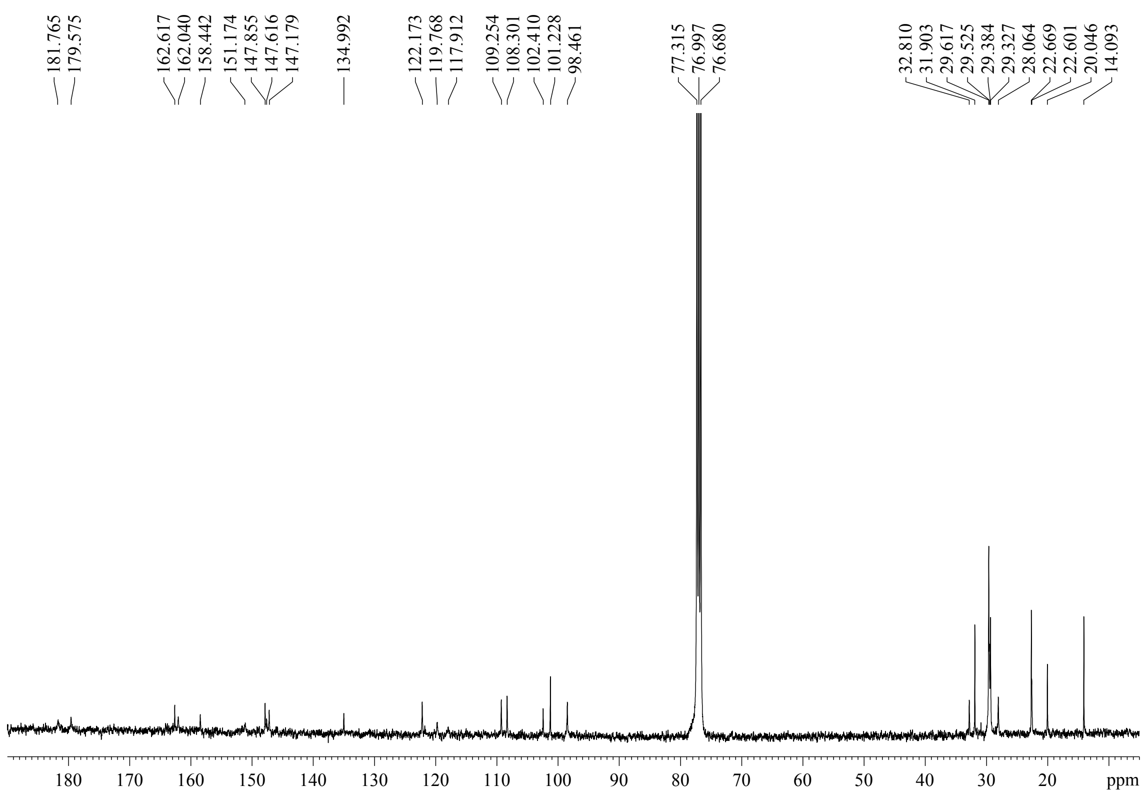

^{1}H NMR ($CDCl_3$, 400 MHz) of compound 3i

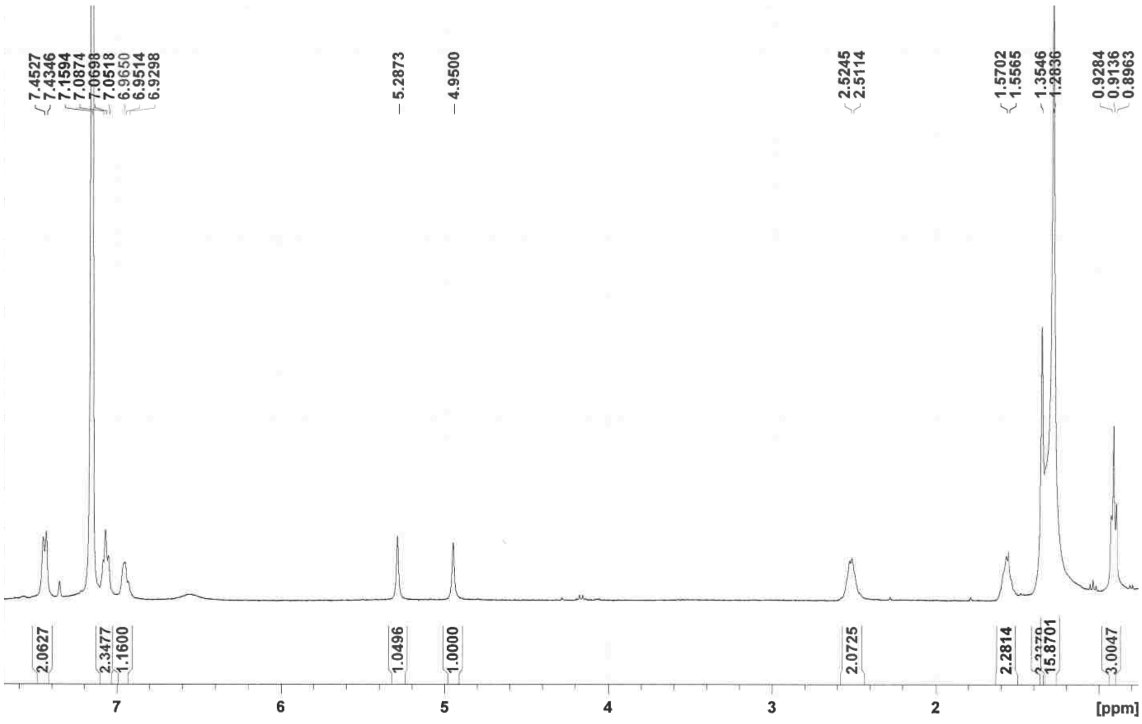

^{13}C NMR ($CDCl_3$, 100 MHz) of compound 3i

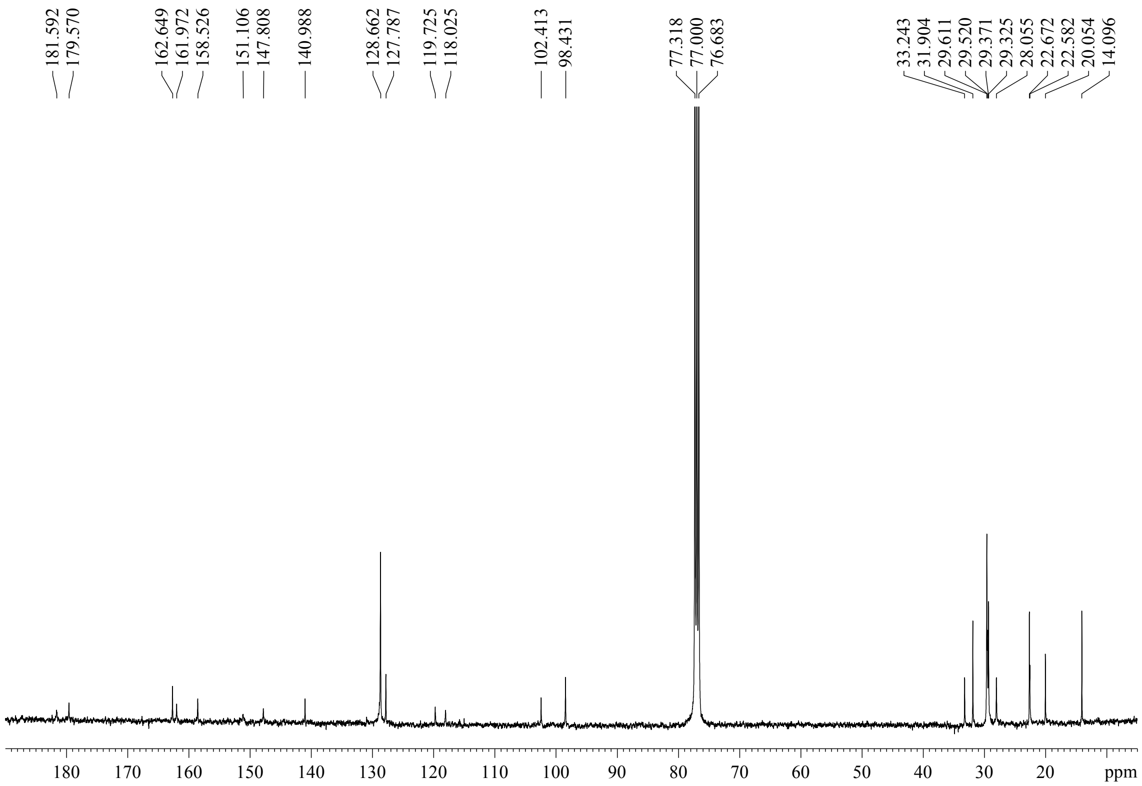

^{1}H NMR ($CDCl_3$, 400 MHz) of compound 3j

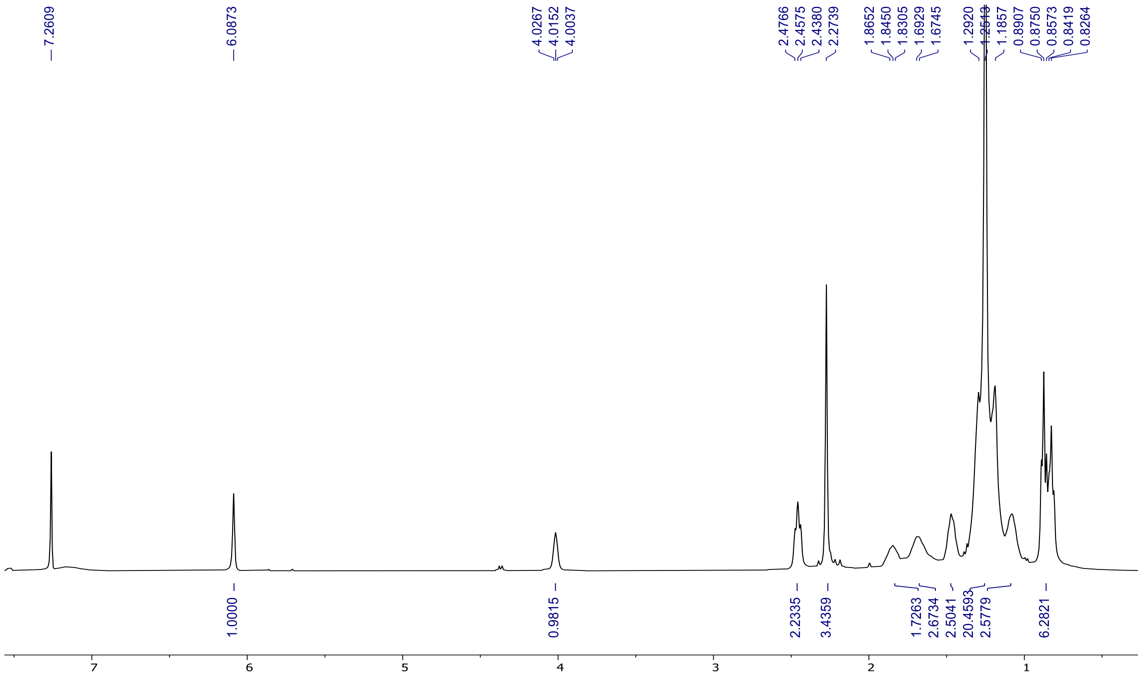

^{13}C NMR ($CDCl_3$, 100 MHz) of compound 3j

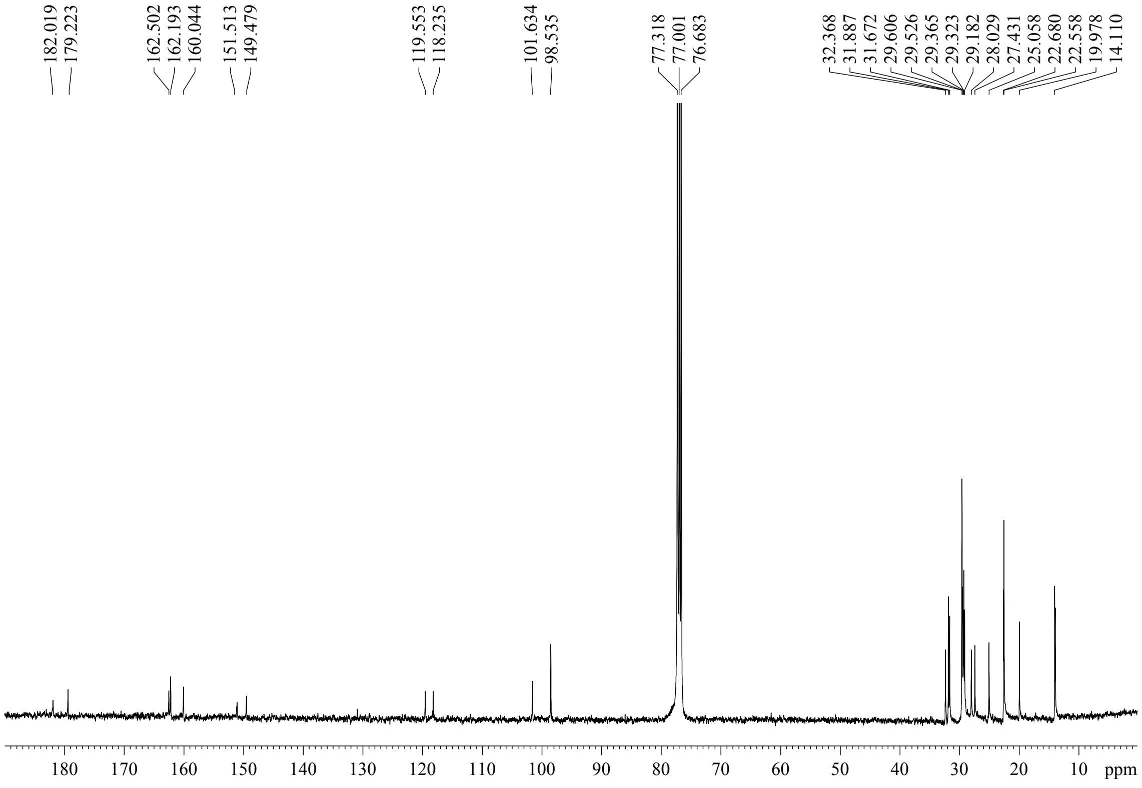

¹H NMR (CDCl₃, 400 MHz) of compound 3k

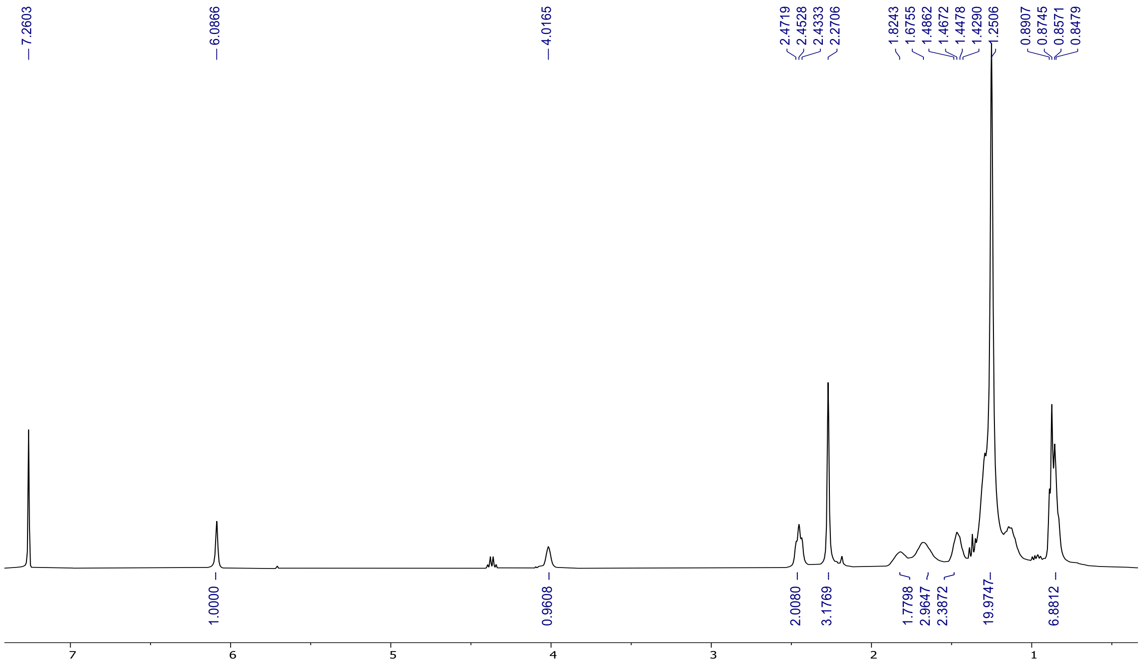

¹³C NMR (CDCl₃, 100 MHz) of compound 3k

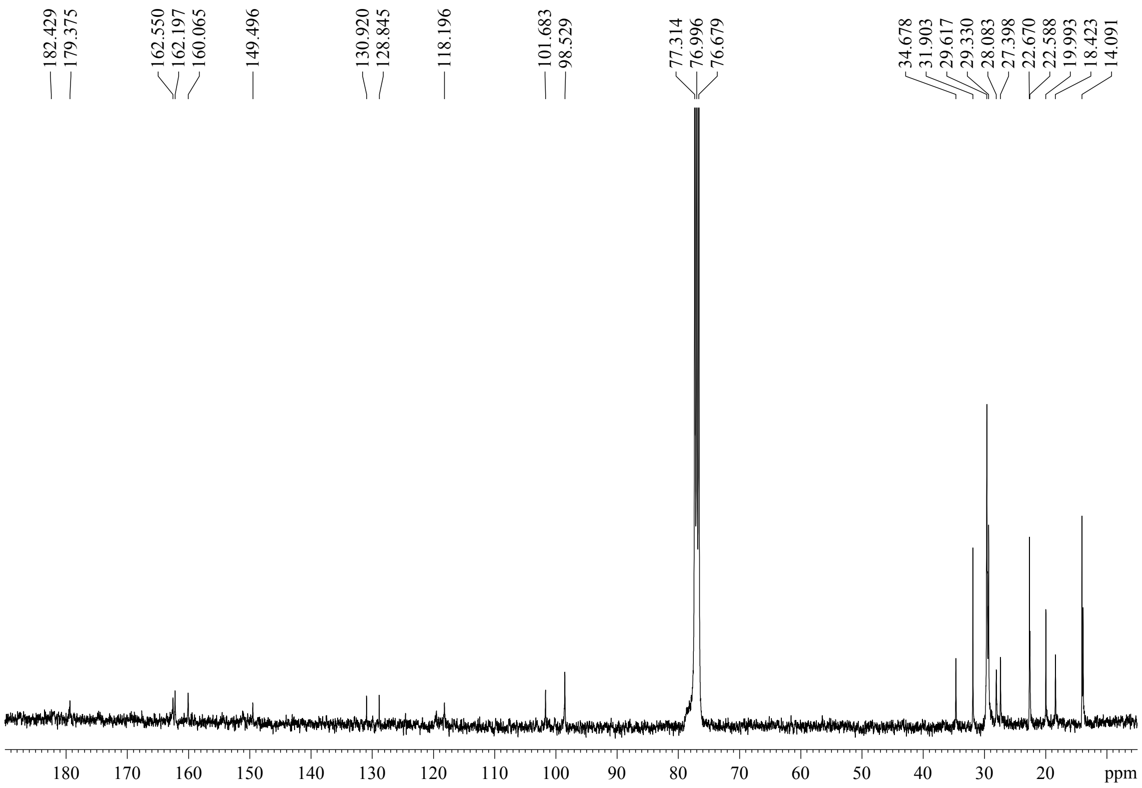

^{1}H NMR ($CDCl_3$, 400 MHz) of compound 3l

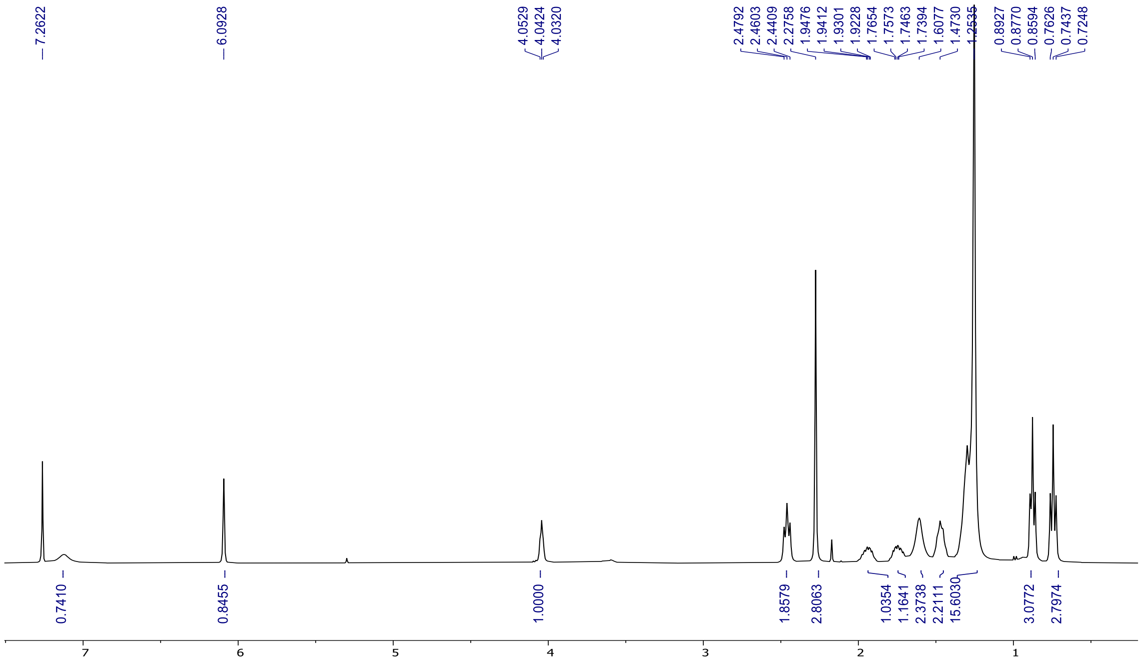

^{13}C NMR ($CDCl_3$, 100 MHz) of compound 3l

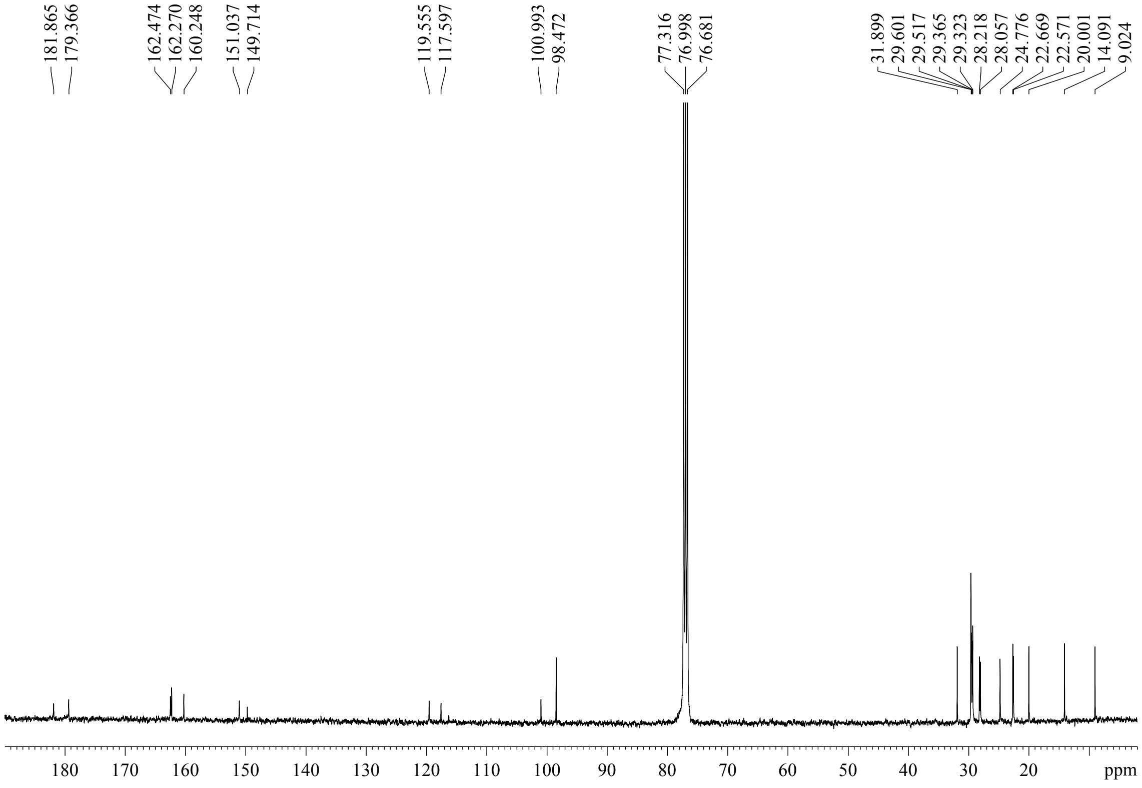

^{1}H NMR spectrum (C_6D_6, 400 MHz) of compound 4a

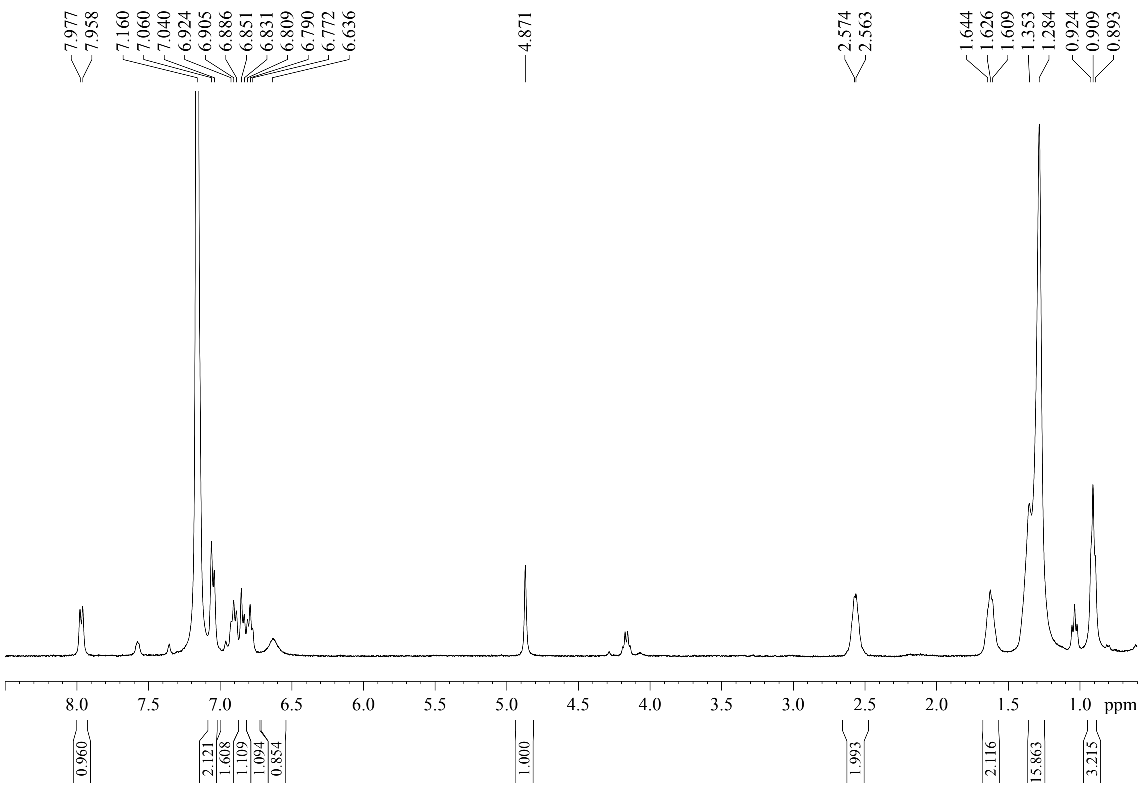

^{13}C NMR spectrum ($CDCl_3$, 100 MHz) of compound 4a

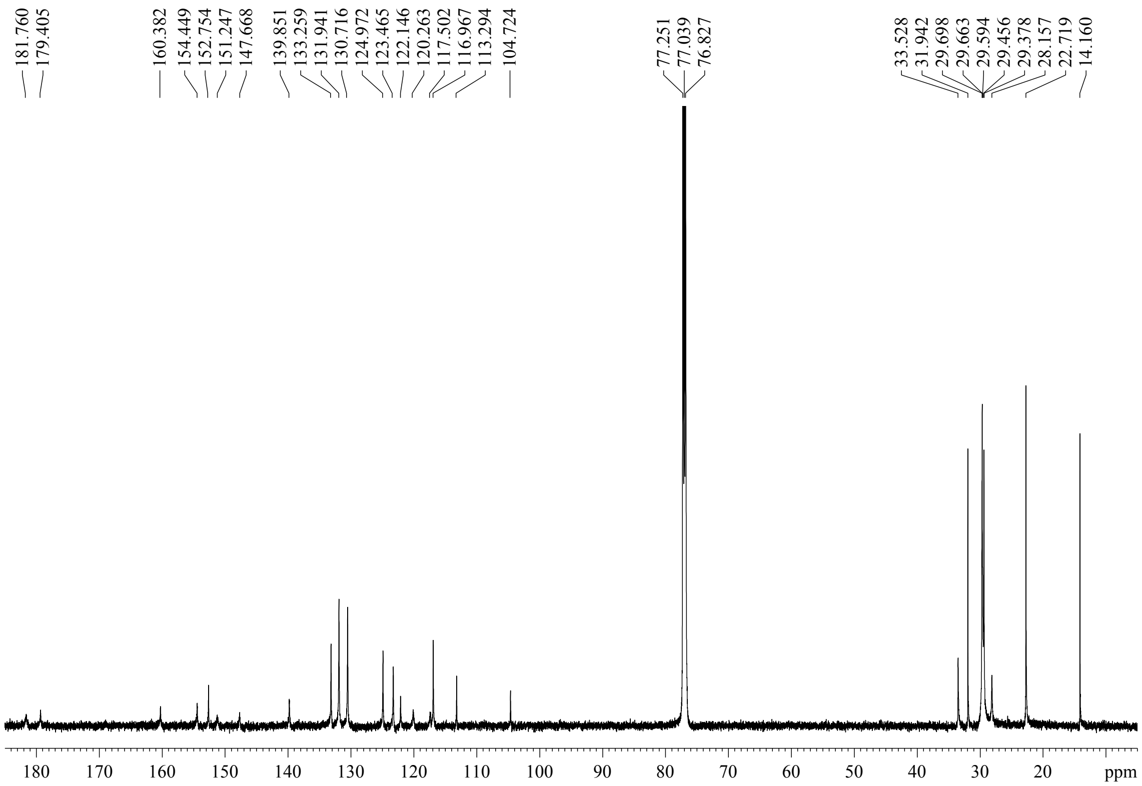

^{1}H NMR (C_6D_6, 400 MHz) of compound 4b

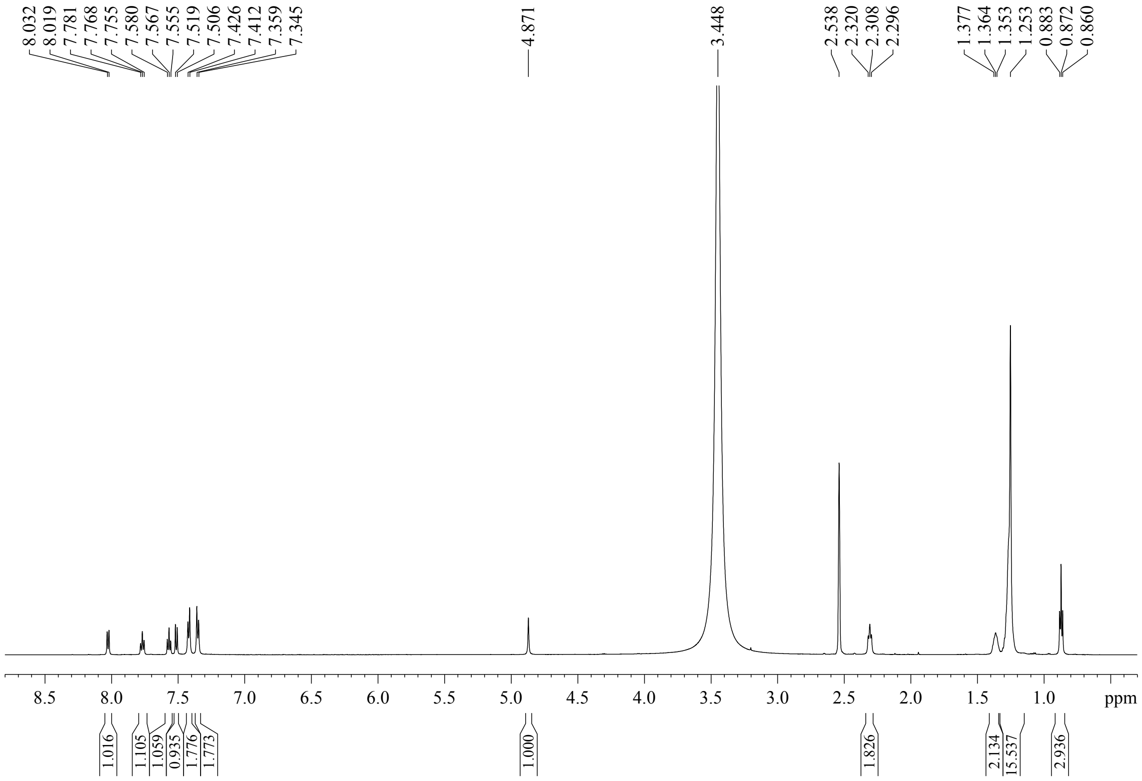

^{13}C NMR (DMSO-d6, 150 MHz) of compound 4b

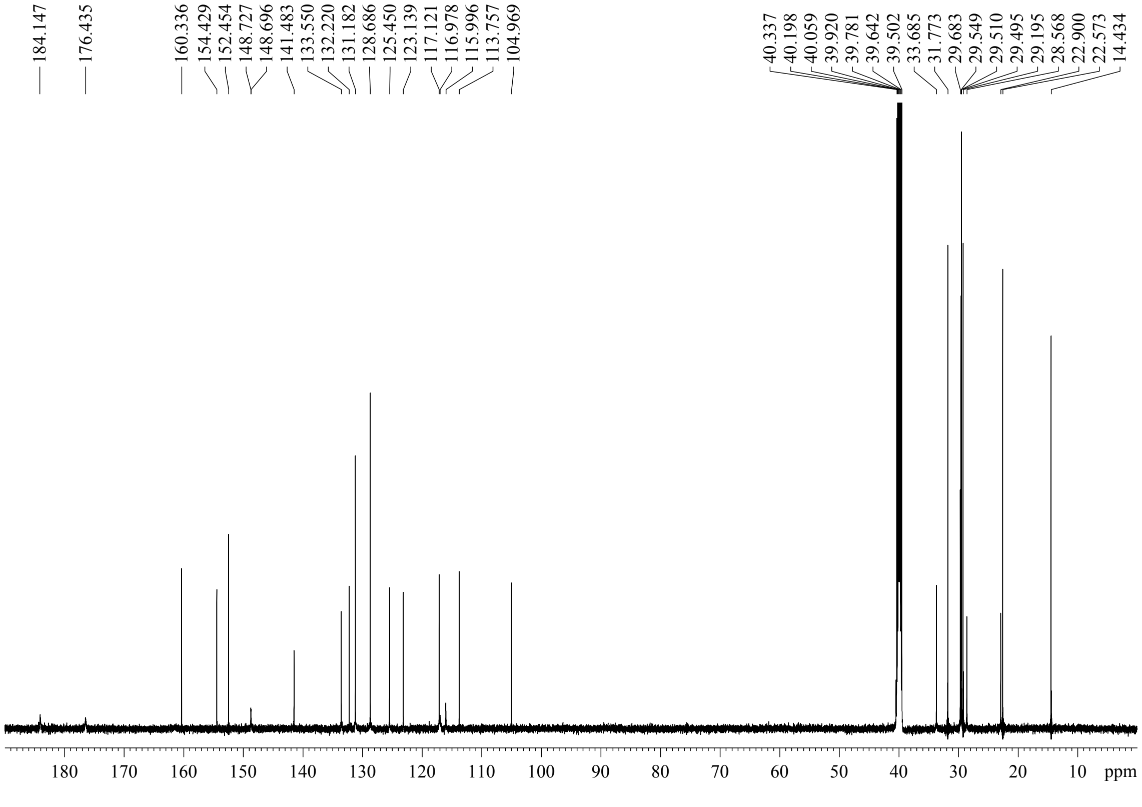

^{1}H NMR (C_6D_6, 400 MHz) of compound 4c

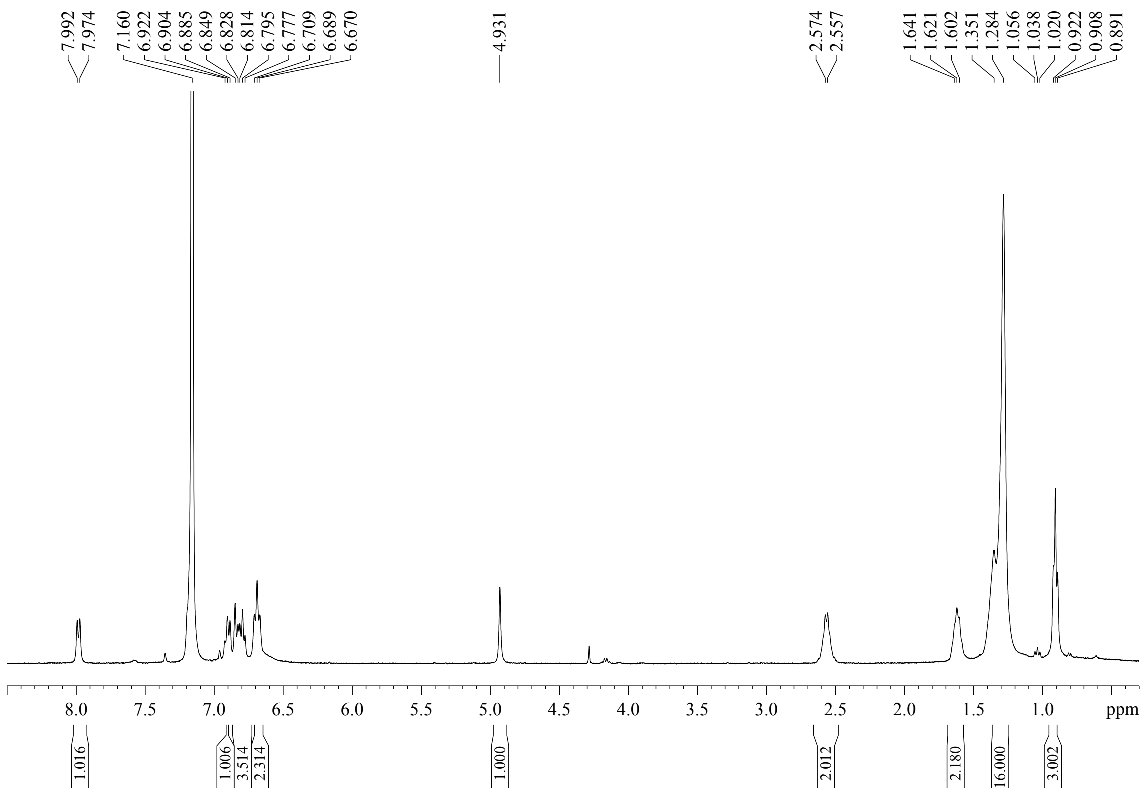

^{13}C NMR ($CDCl_3$, 100 MHz) of compound 4c

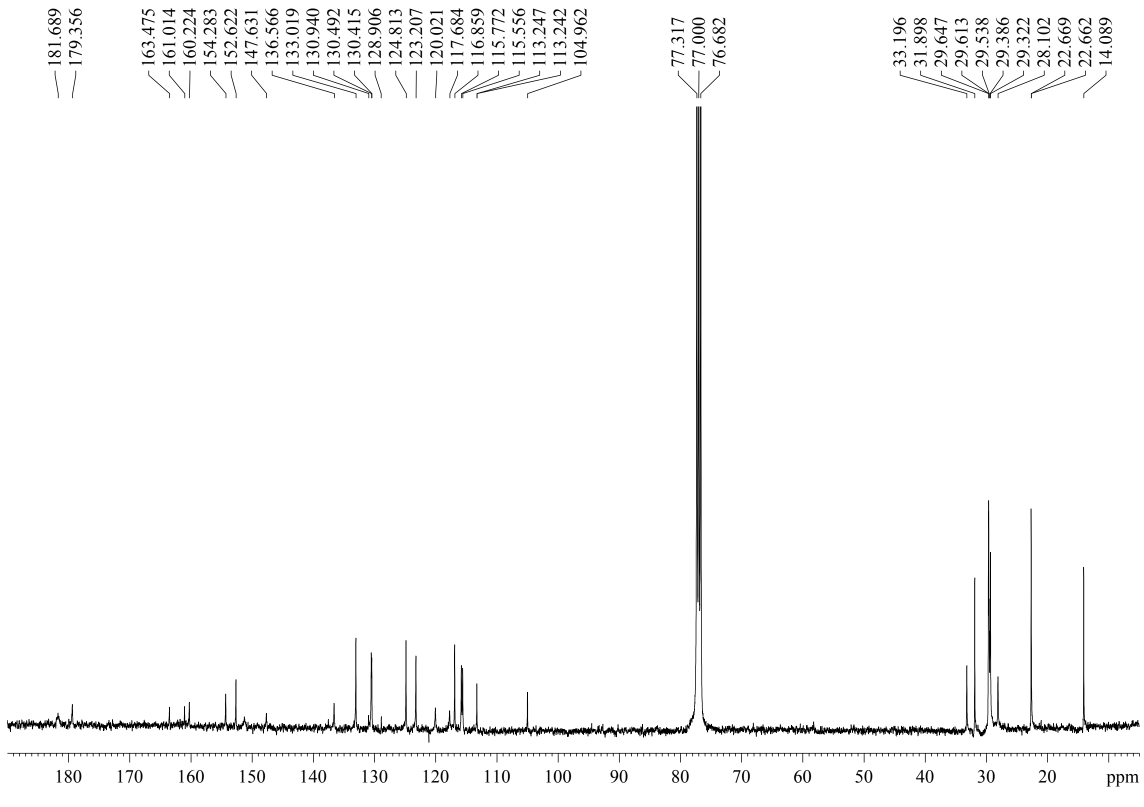

^{1}H-NMR spectrum (C_6D_6, 500 MHz) of compound 4d

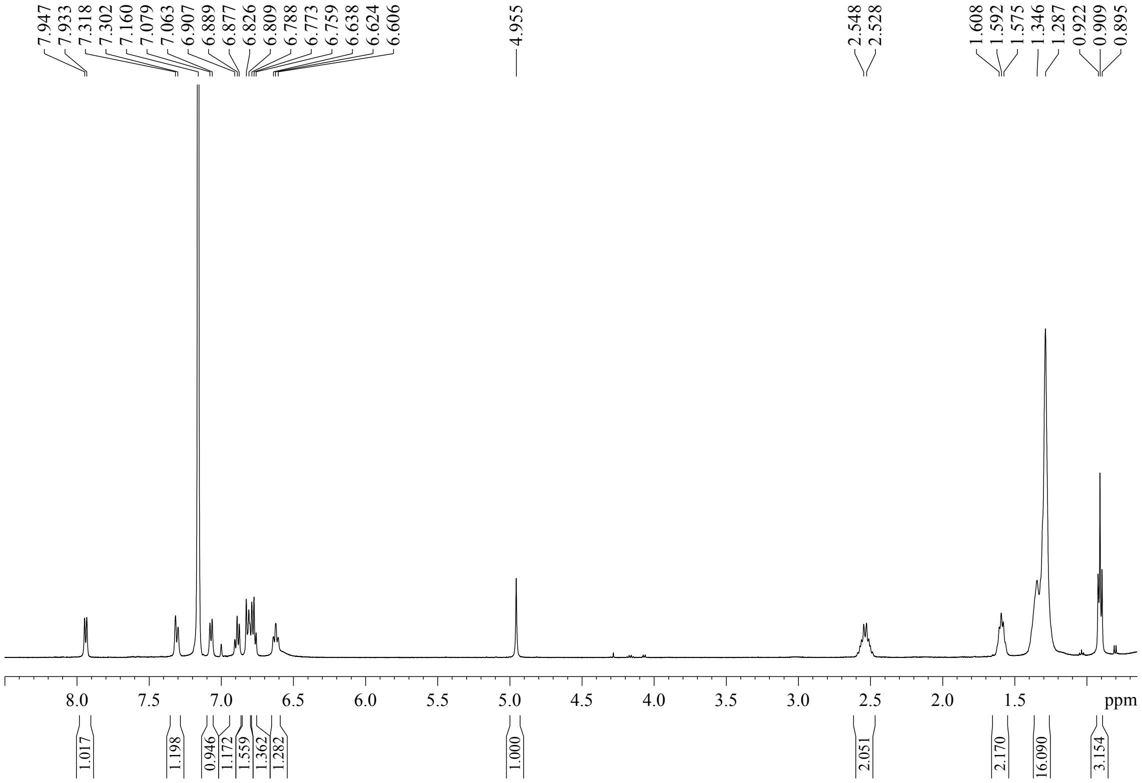

^{13}C NMR ($CDCl_3$, 150 MHz) of compound 4d

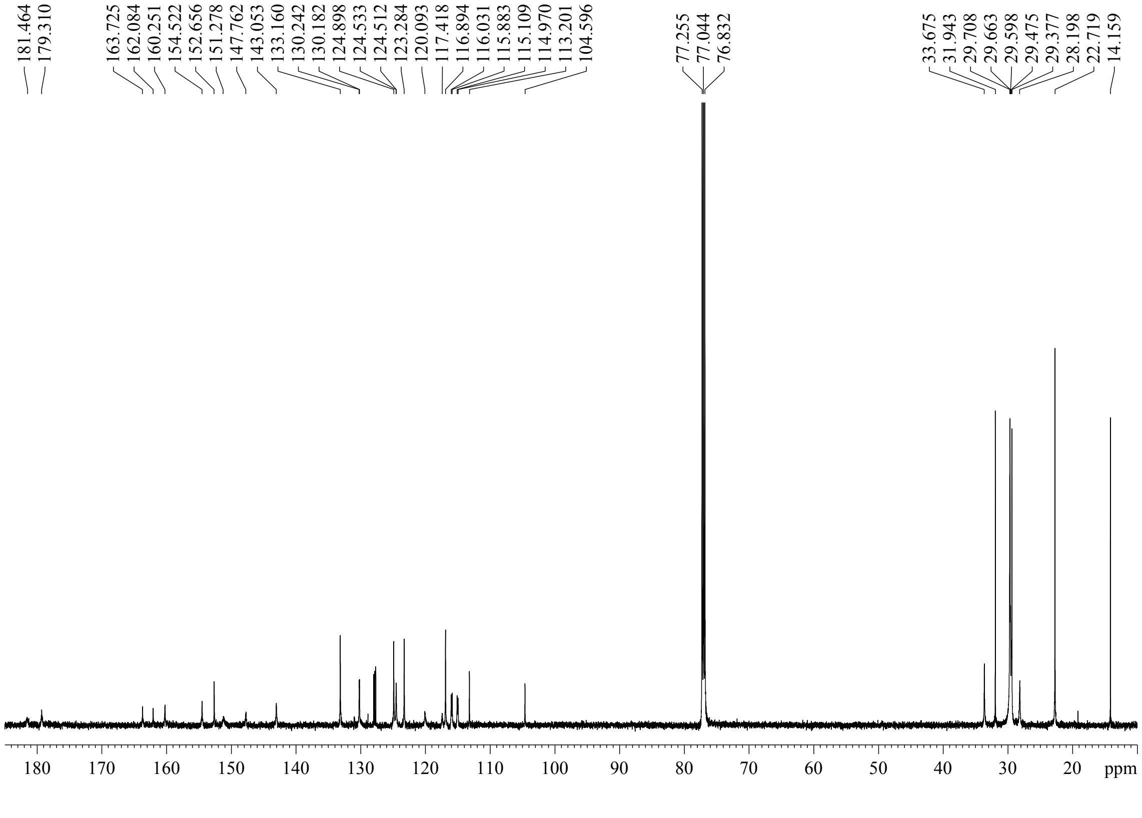

^{1}H NMR (C_6D_6, 400 MHz) of compound 4e

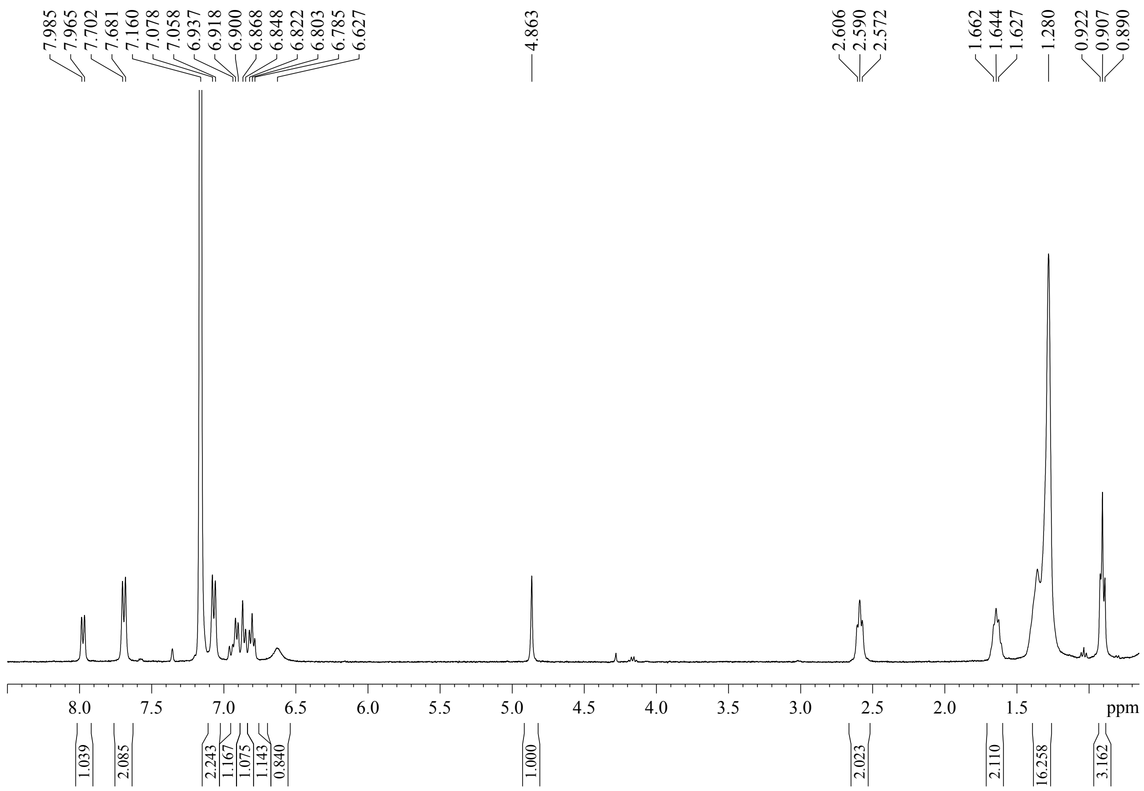

^{13}C NMR spectrum ($CDCl_3$, 100 MHz) of compound 4e

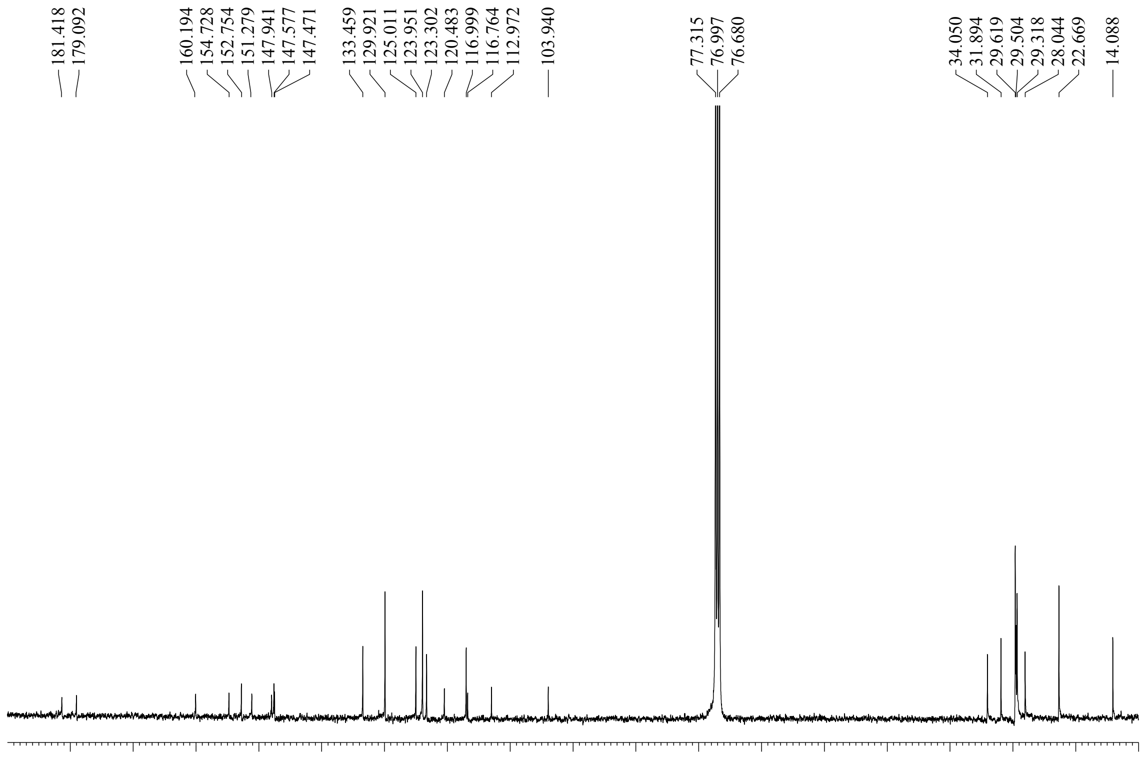

^{1}H NMR (C_6D_6, 400 MHz) of compound 4f

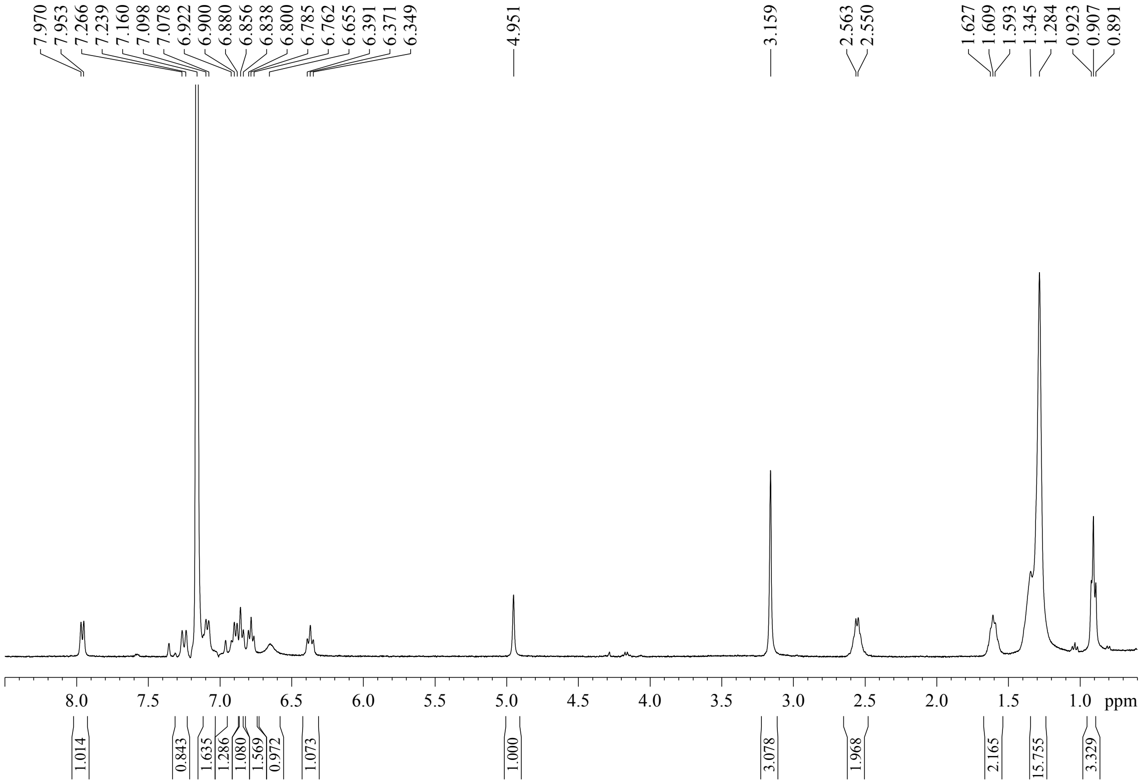

^{13}C NMR (DMSO-d6, 150 MHz) of compound 4f

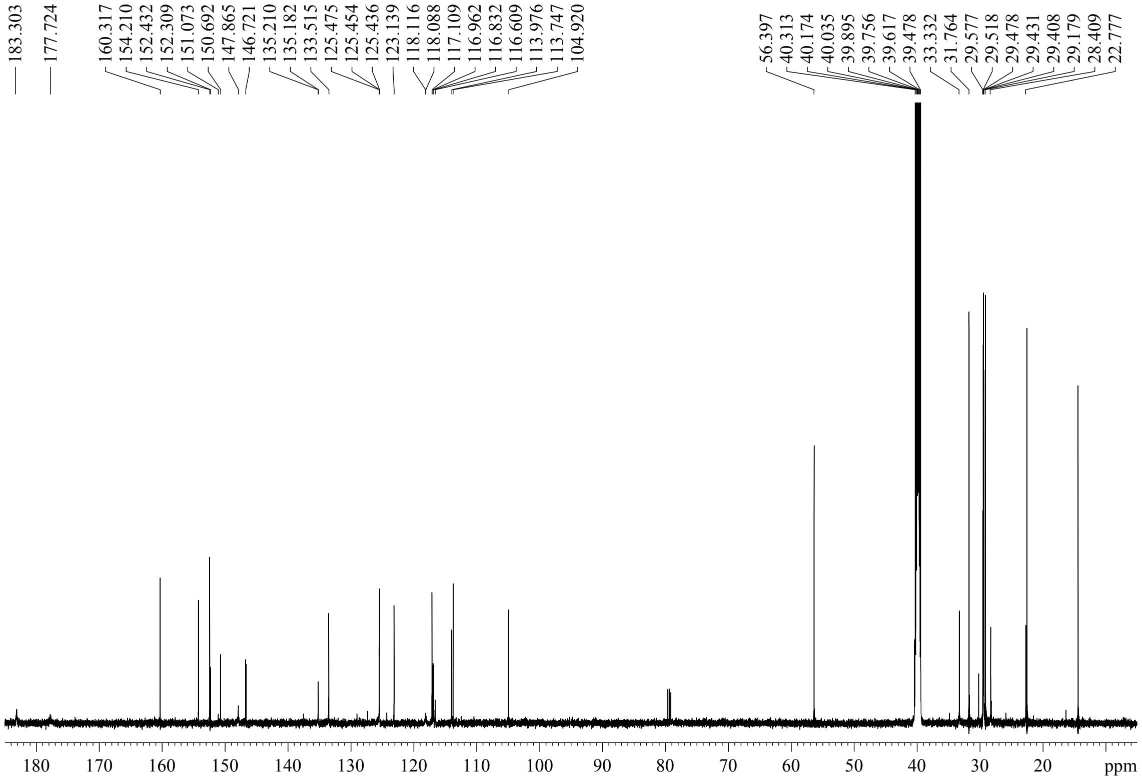

^{1}H NMR (C_6D_6, 400 MHz) of compound 4g

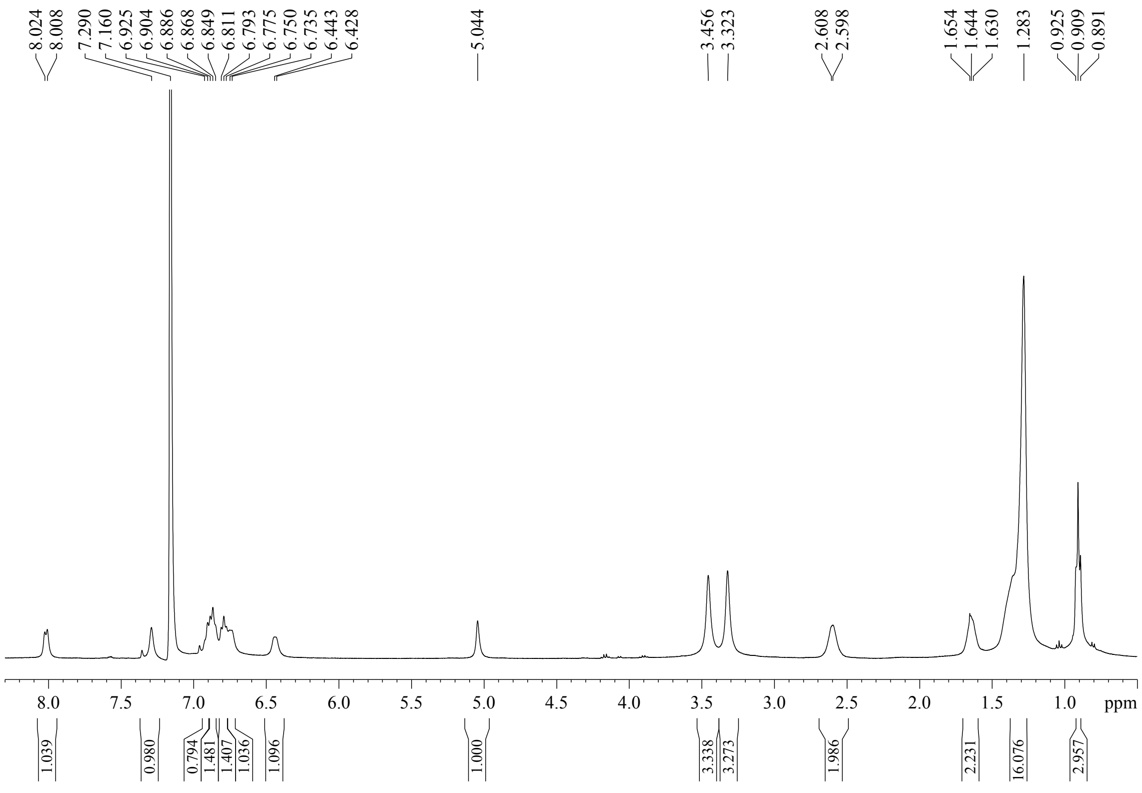

^{13}C NMR ($CDCl_3$, 100 MHz) of compound 4g

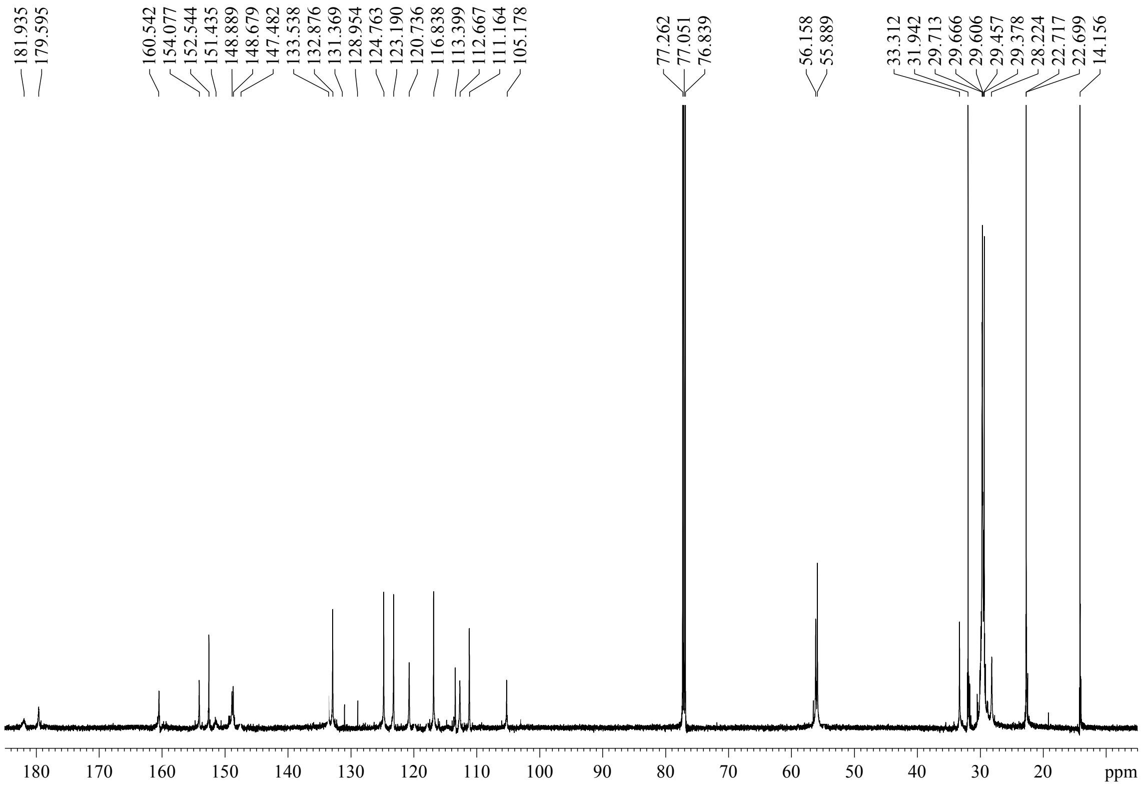

^{1}H NMR (C_6D_6, 400 MHz) of compound 4h

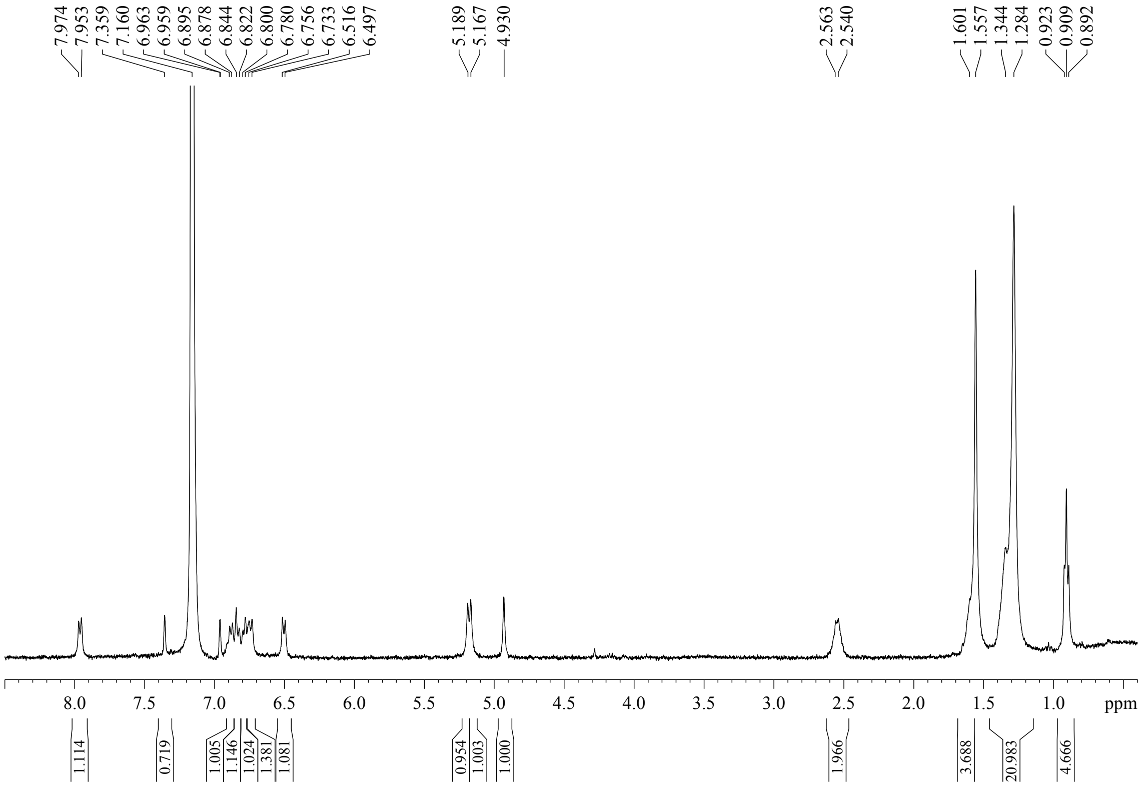

^{13}C NMR (DMSO-d_6, 150 MHz) of compound 4h

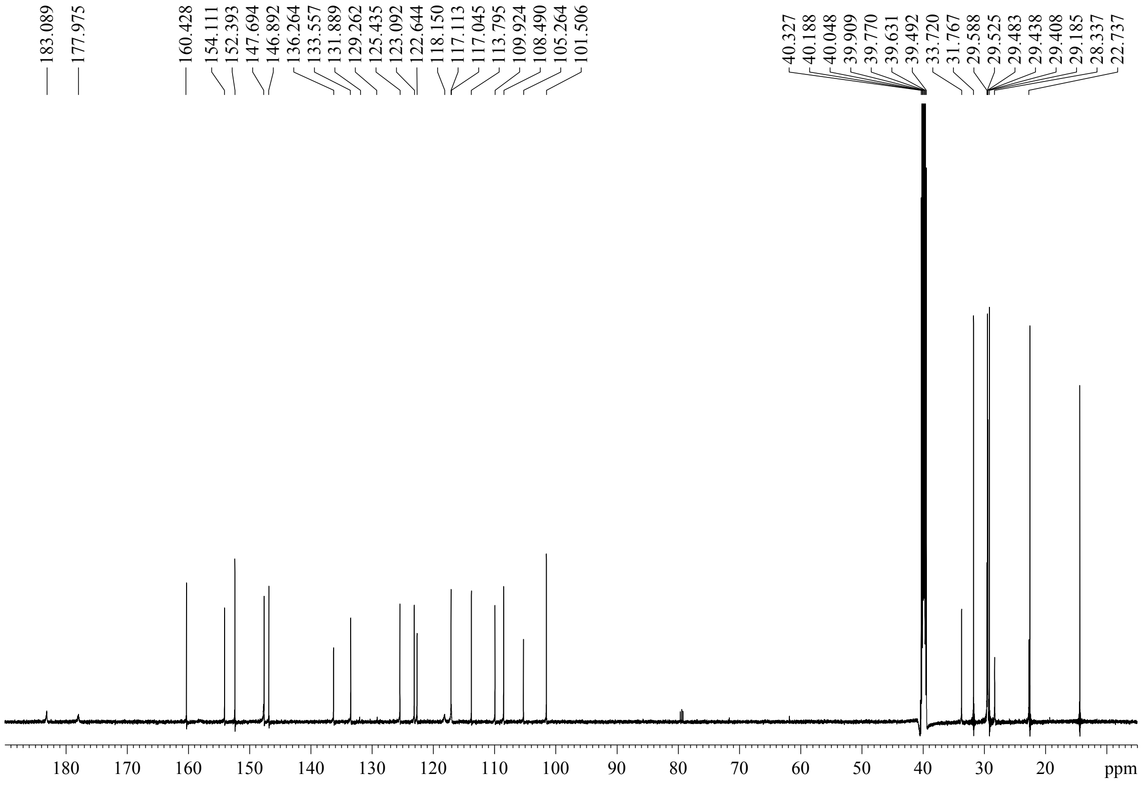

^{1}H NMR (C_6D_6, 400 MHz) of compound 4i

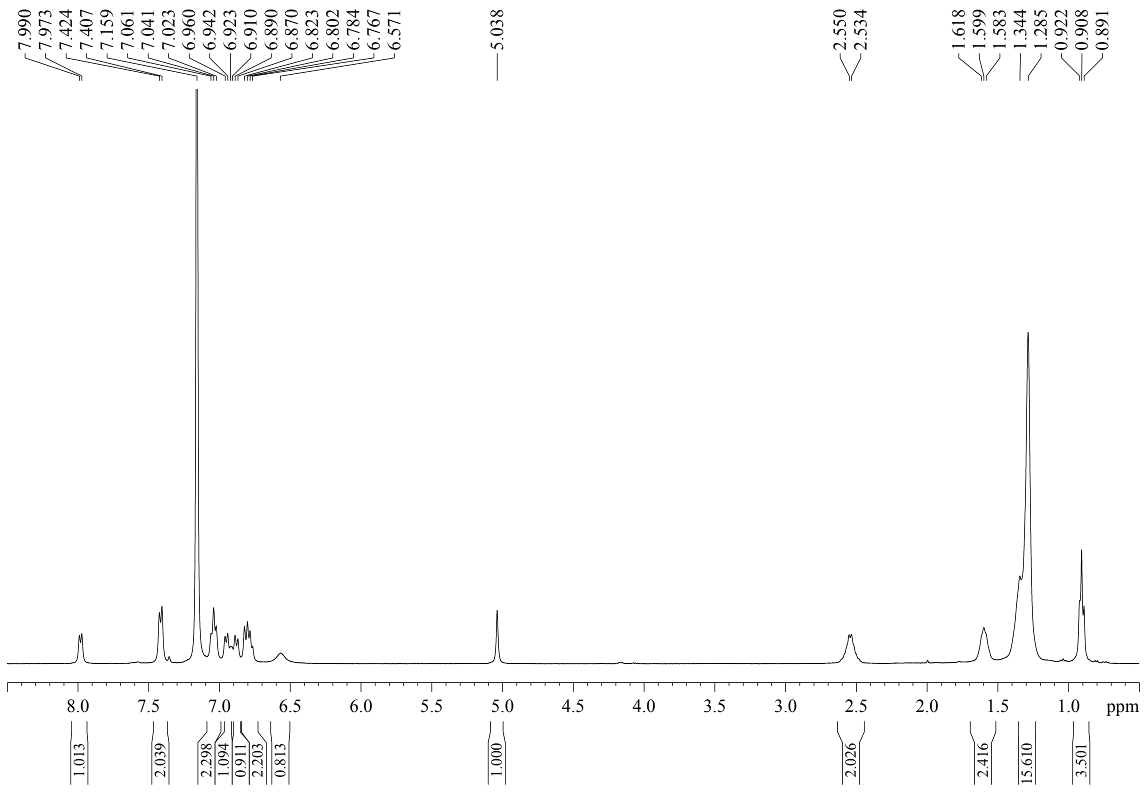

^{13}C NMR ($CDCl_3$, 100 MHz) of compound 4i

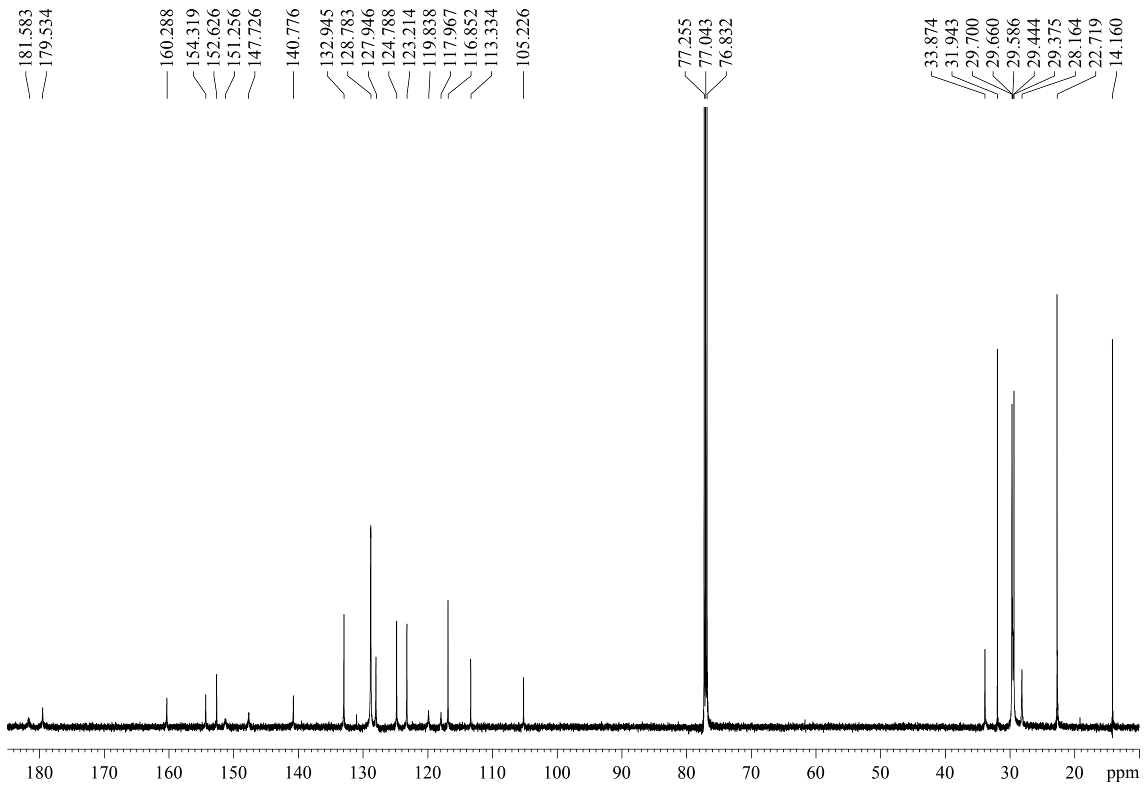

^{1}H NMR (CD_3OD, 400 MHz) of compound 4j

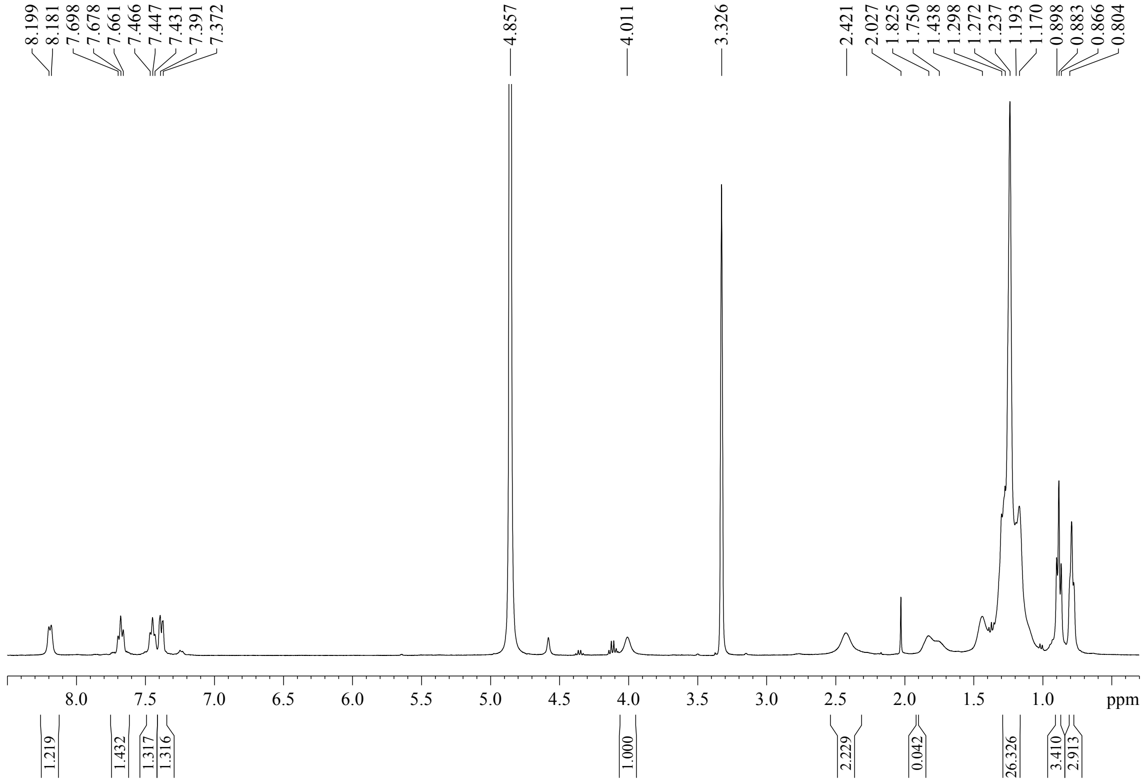

^{13}C NMR ($CDCl_3$, 100 MHz) of compound 4j

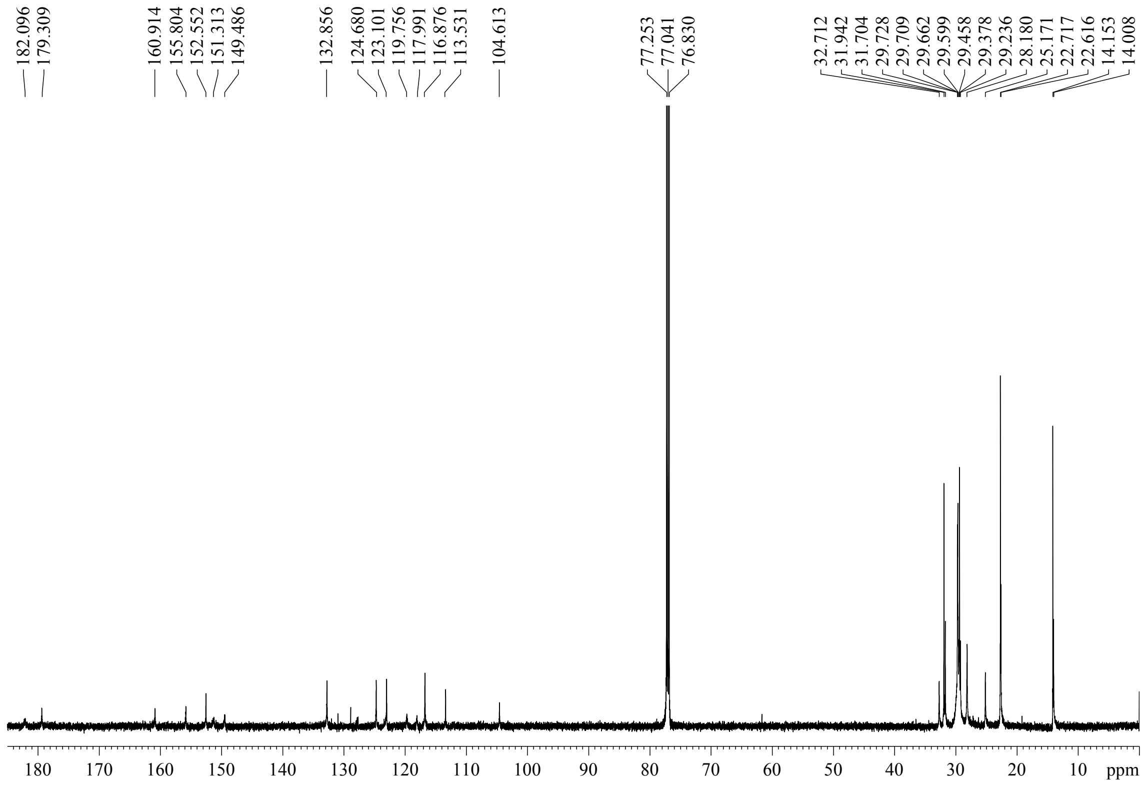

¹H NMR ($CDCl_3$, 400 MHz) of compound 4k

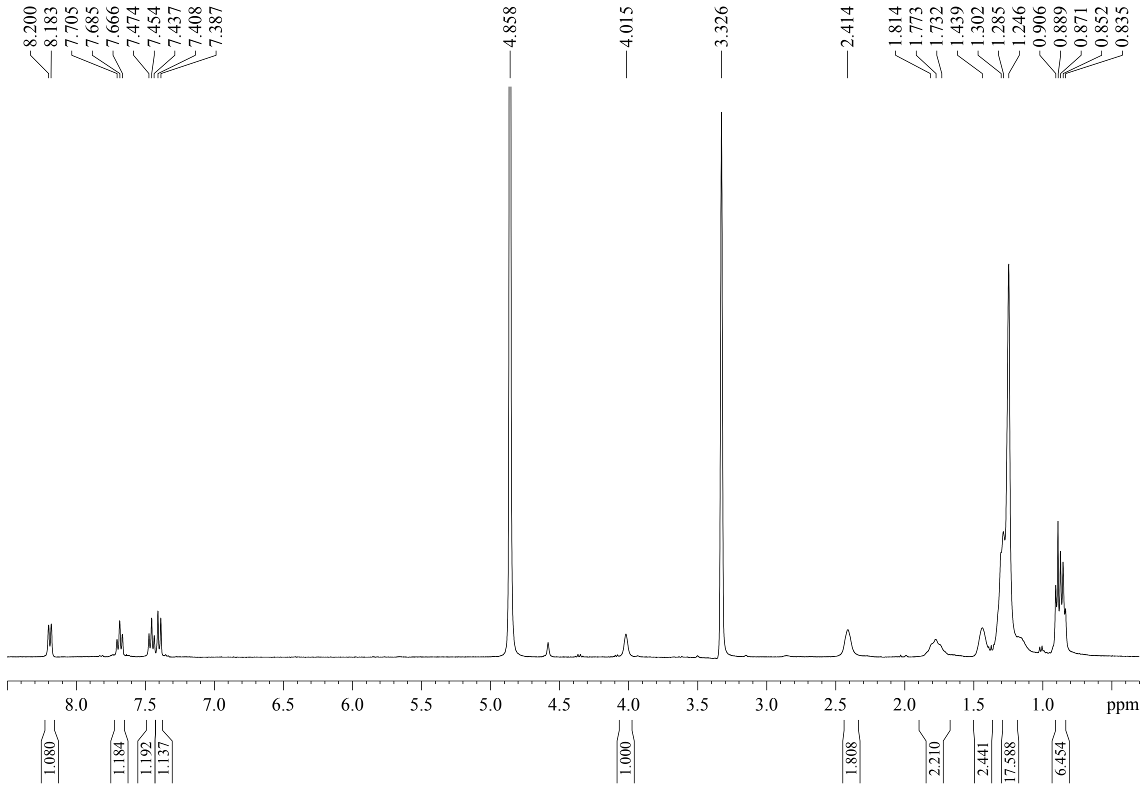

^{1}H NMR ($CDCl_3$, 400 MHz) of compound 4l

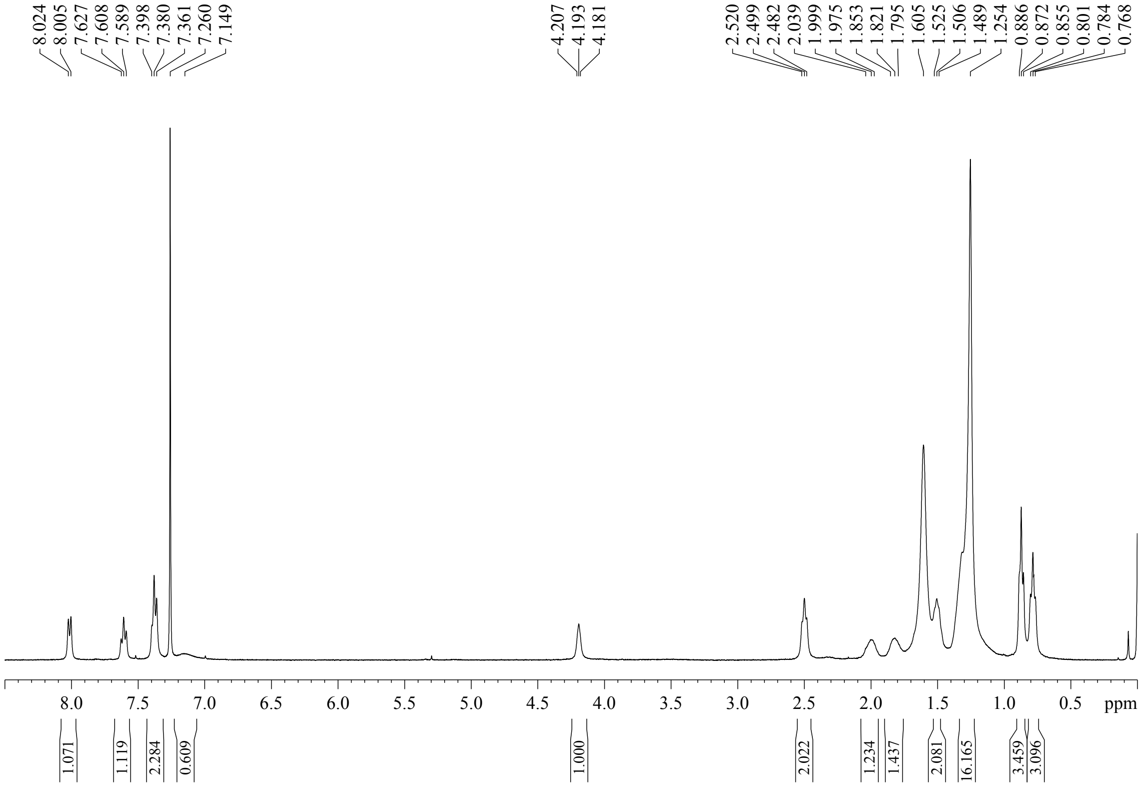

^{13}C NMR ($CDCl_3$, 100 MHz) of compound 4l

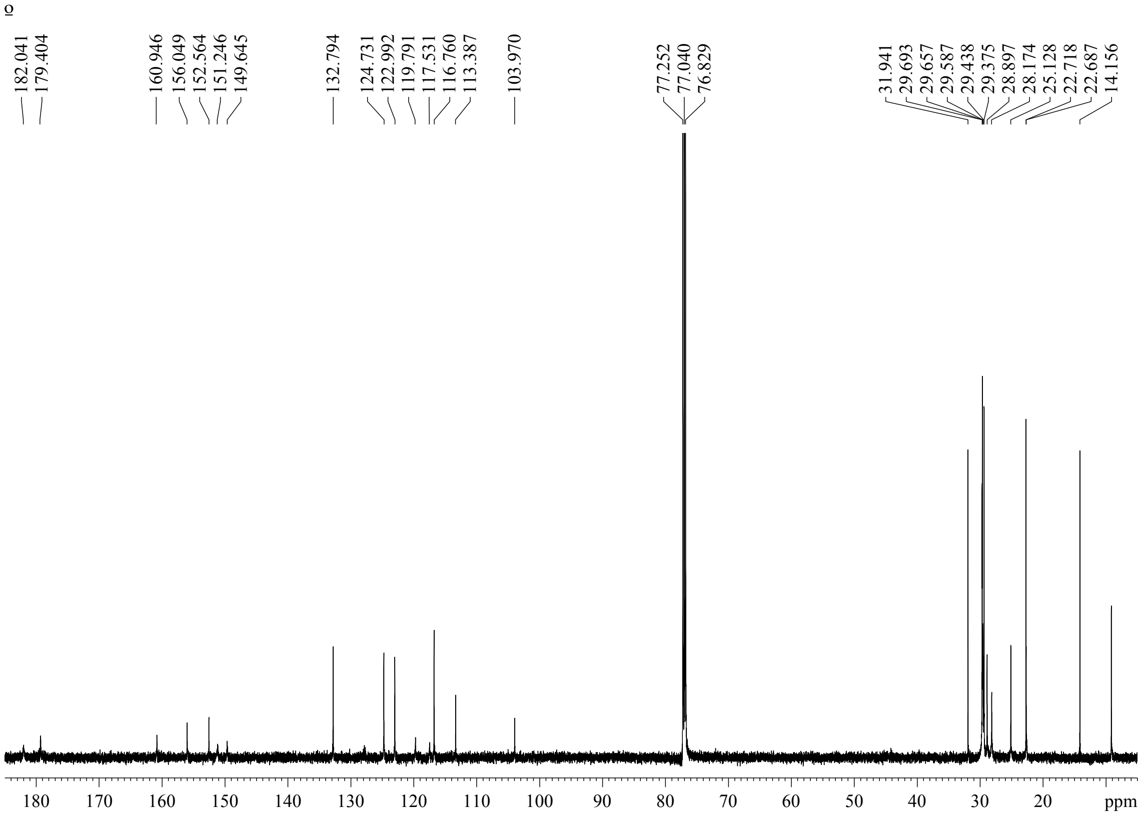

^{1}H NMR (C_6D_6, 400 MHz) of compound 5a

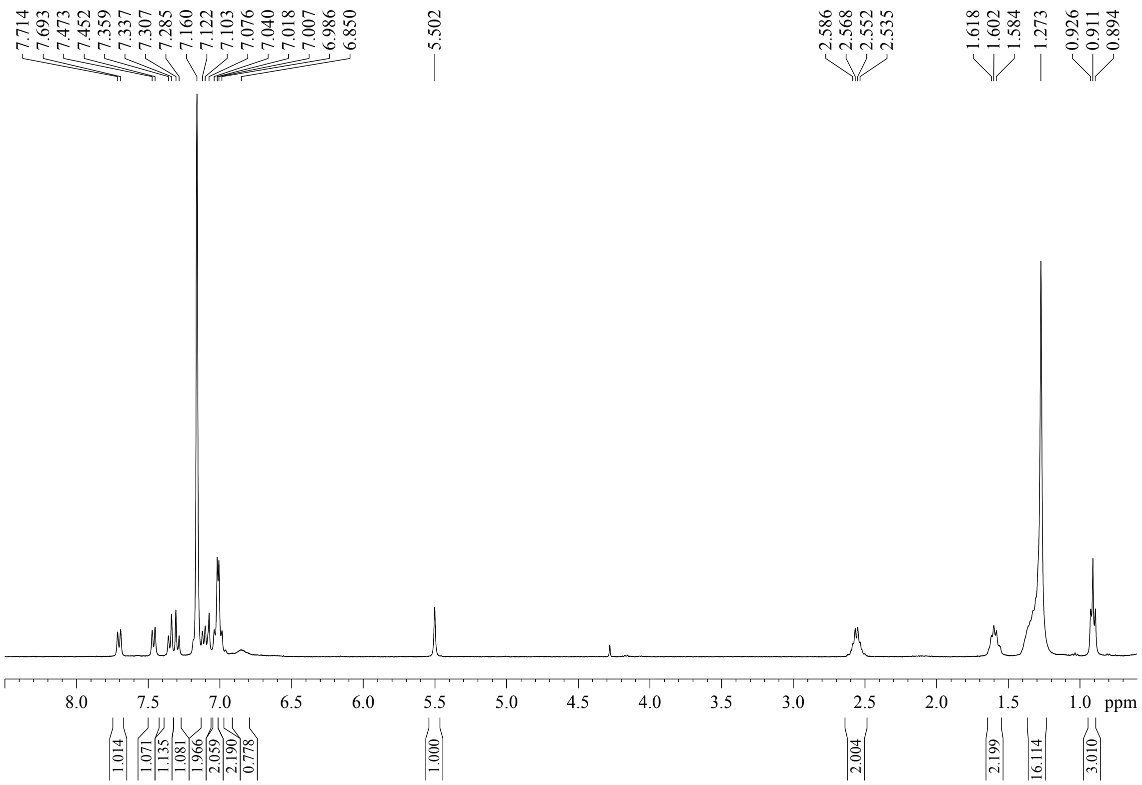

^{13}C NMR ($CDCl_3$, 100 MHz) of compound 5a

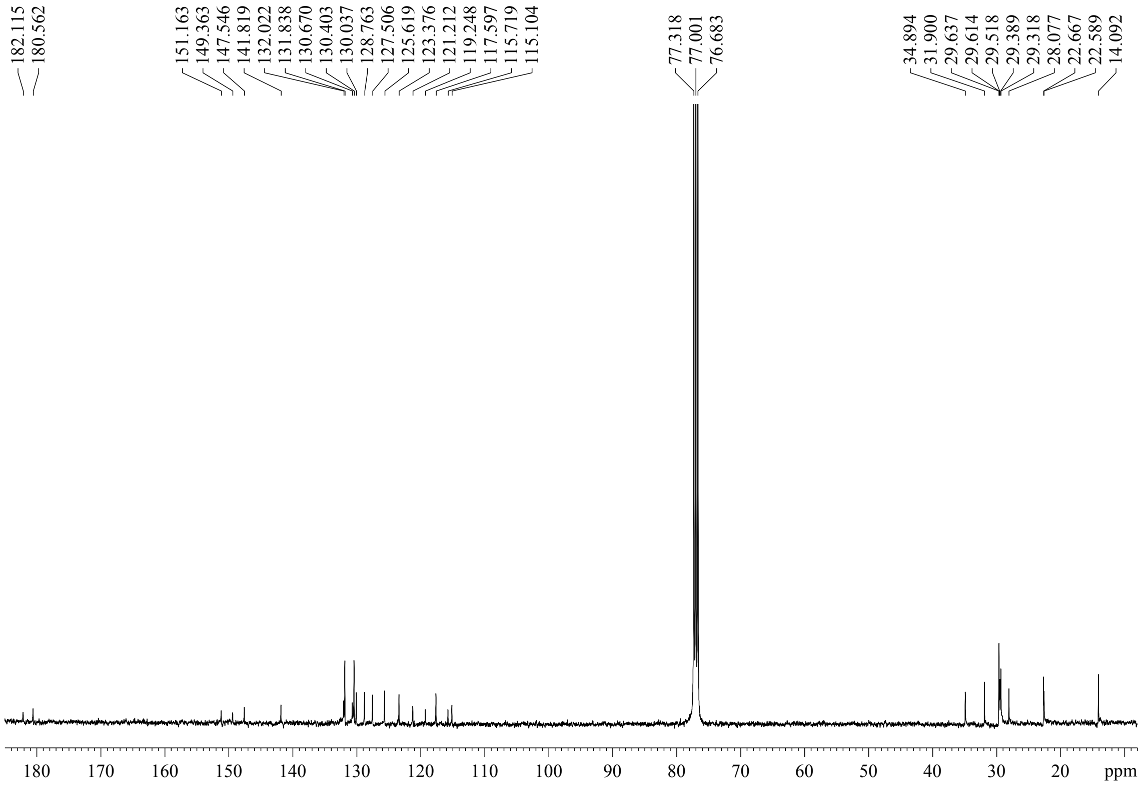

¹H NMR (C_6D_6, 400 MHz) of compound 5b

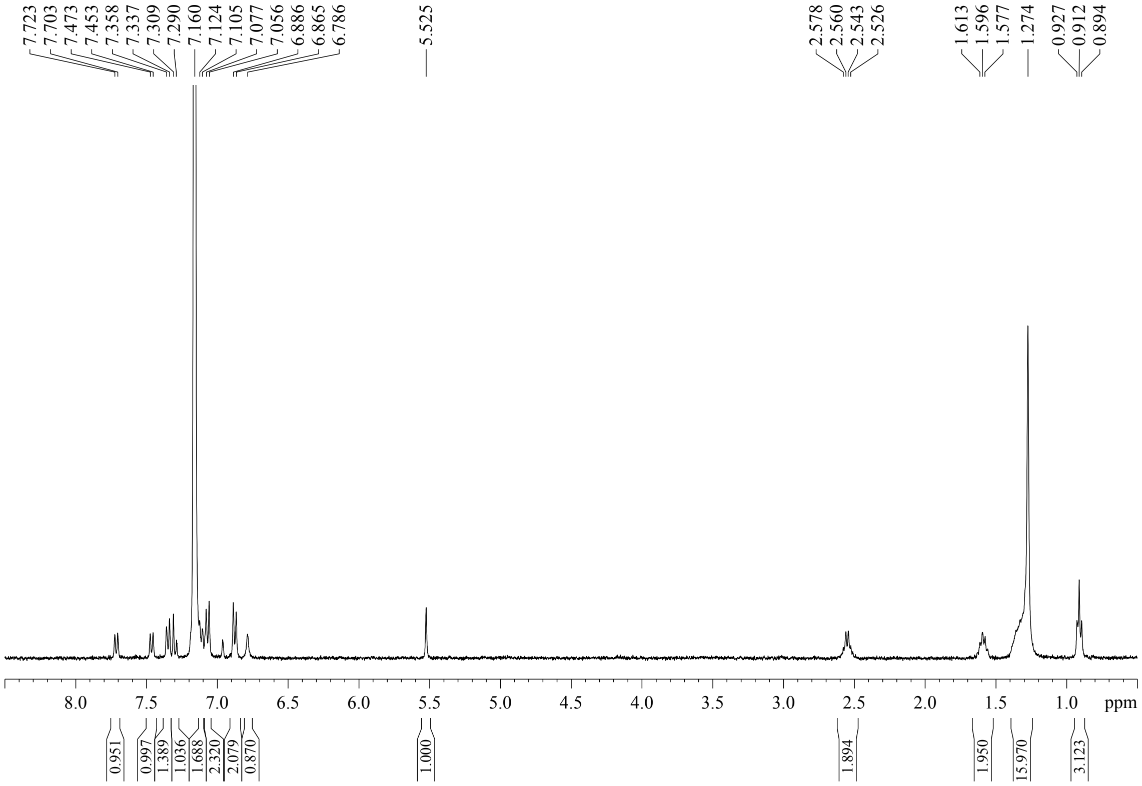

¹³C NMR (DMSO-d_6, 100 MHz) of compound 5b

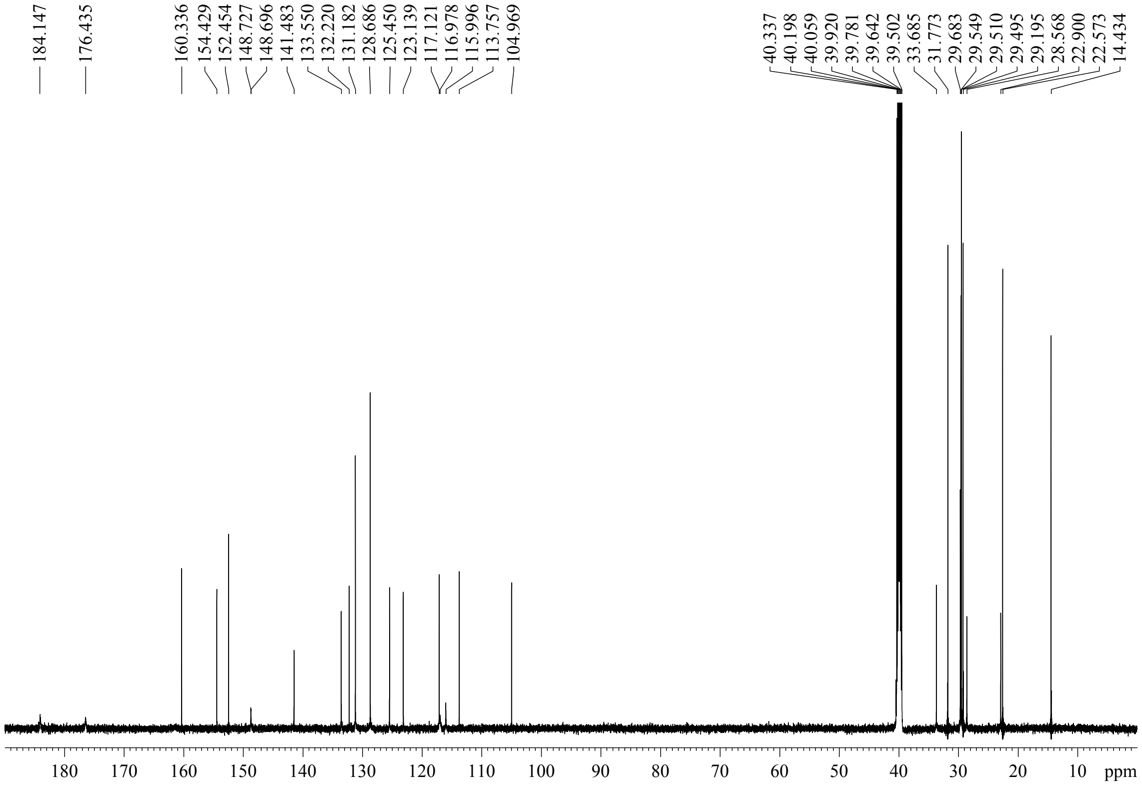

^{1}H NMR (C_6D_6, 400 MHz) of compound 5c

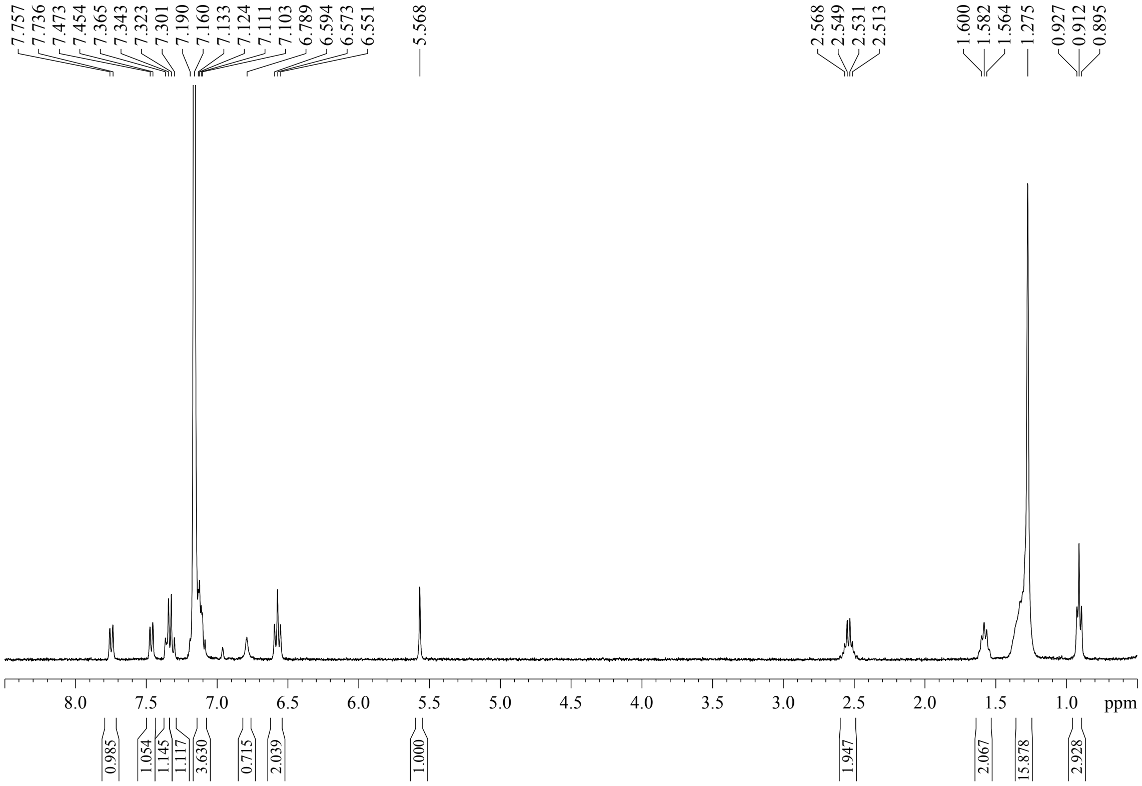

^{13}C NMR ($CDCl_3$, 100 MHz) of compound 5c

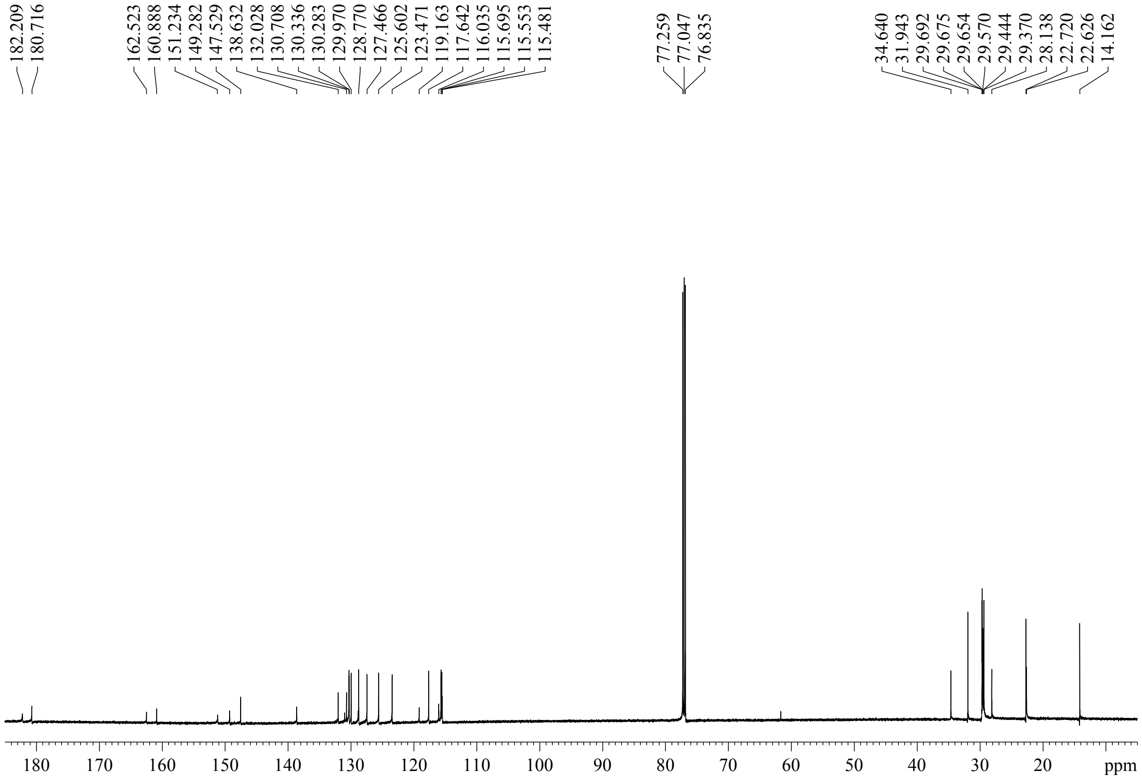

^{1}H NMR (C_6D_6, 400 MHz of compound 5d

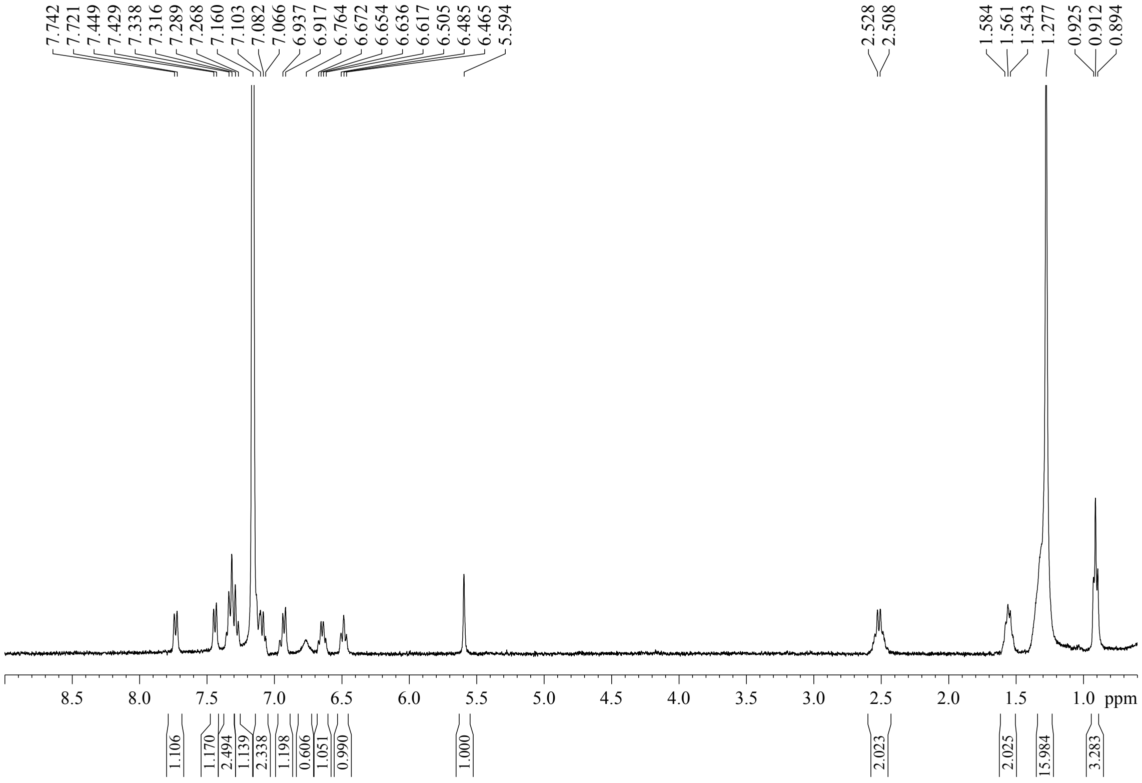

^{13}C NMR ($CDCl_3$, 100 MHz) of compound 5d

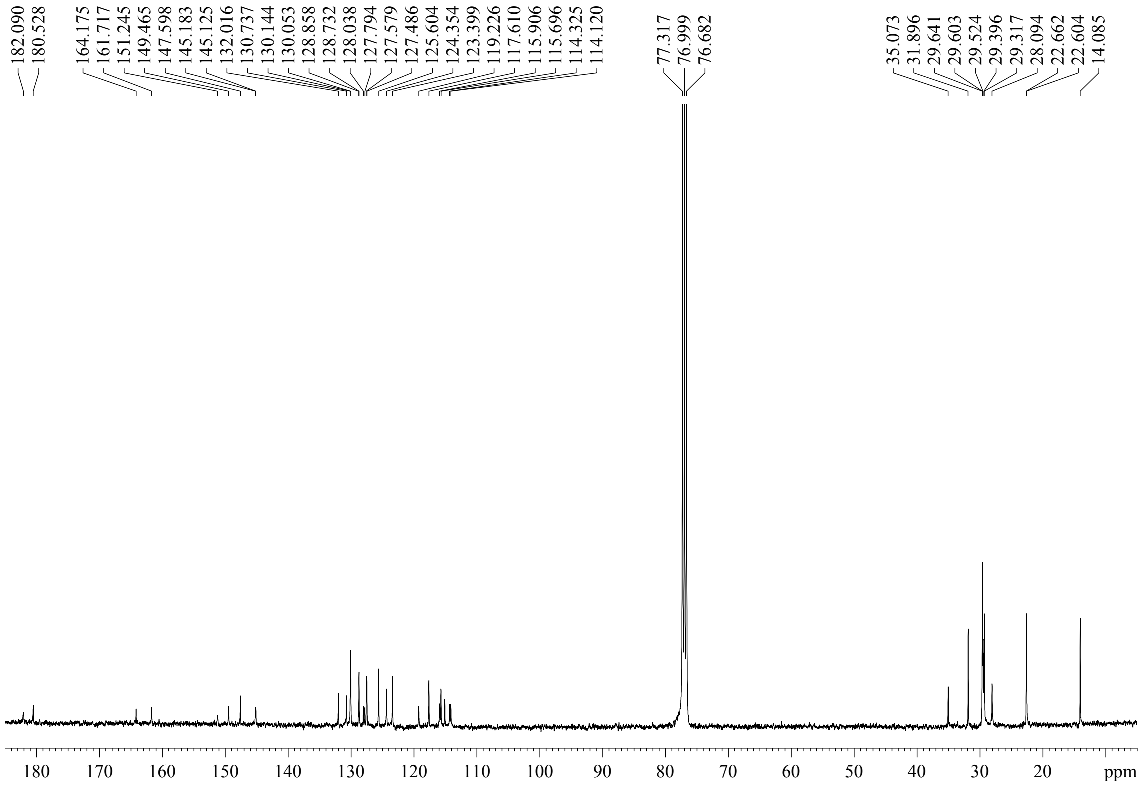

^{1}H NMR ($CDCl_3$, 400 MHz) of compound 5e

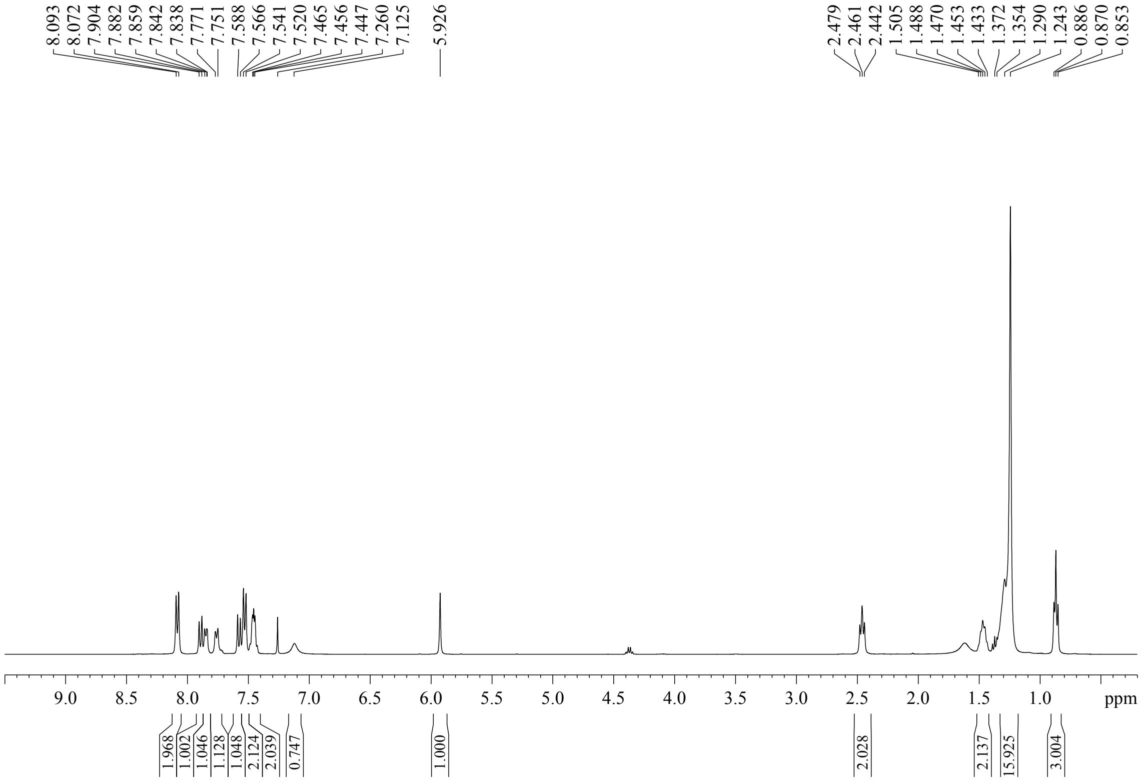

^{13}C NMR ($CDCl_3$, 100 MHz) of compound 5e

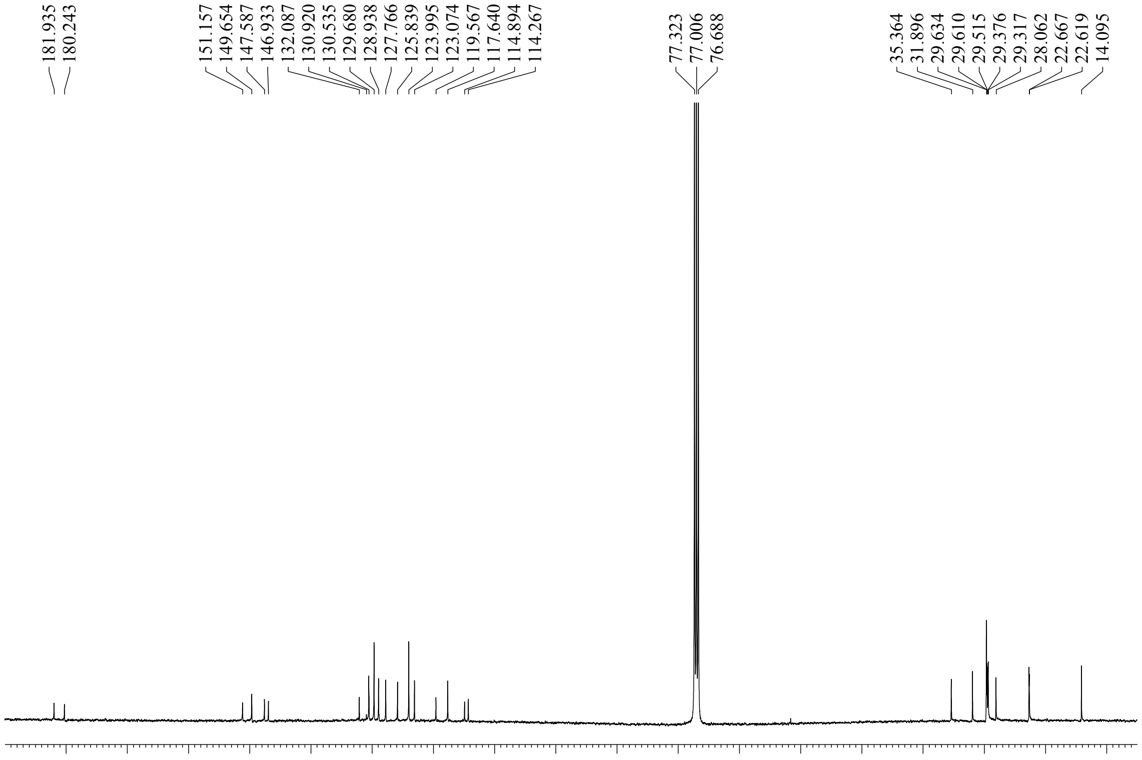

^{1}H NMR (C_6D_6, 400 MHz) of compound 5f

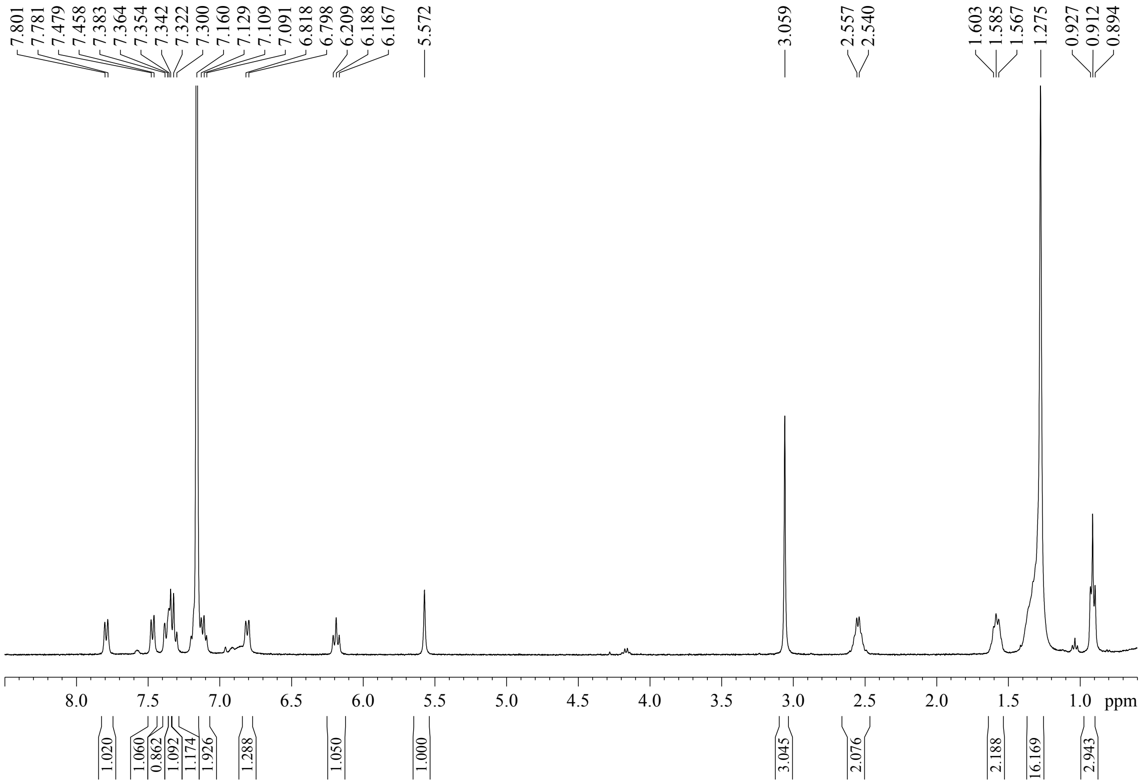

^{13}C NMR ($CDCl_3$, 100 MHz) of compound 5f

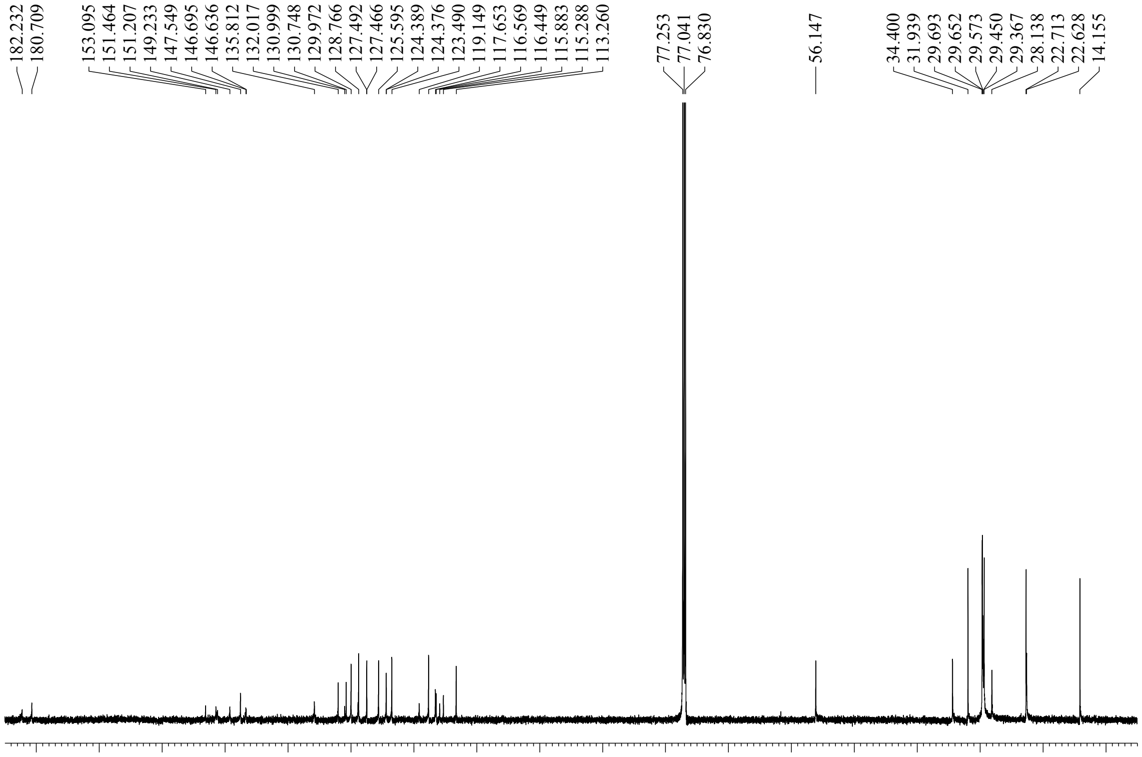

^1H NMR ($CDCl_3$, 400 MHz) of compound 5g

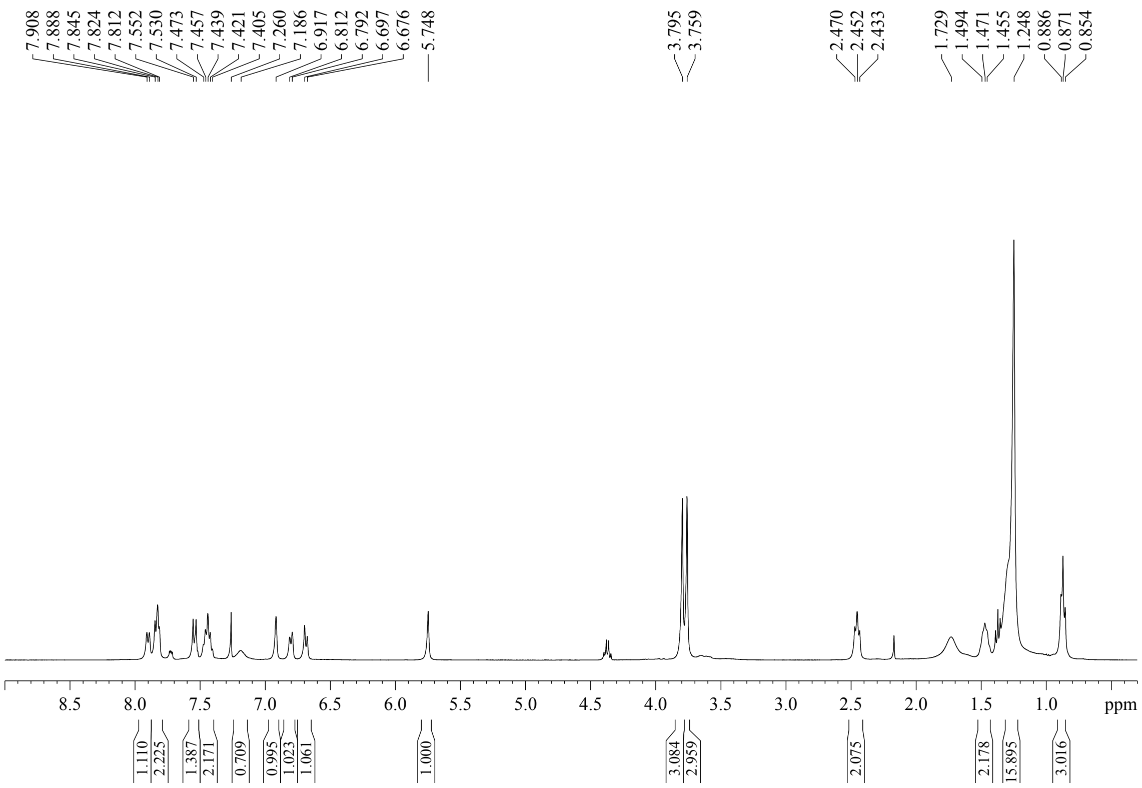

^{13}C NMR ($CDCl_3$, 100 MHz) of compound 5g

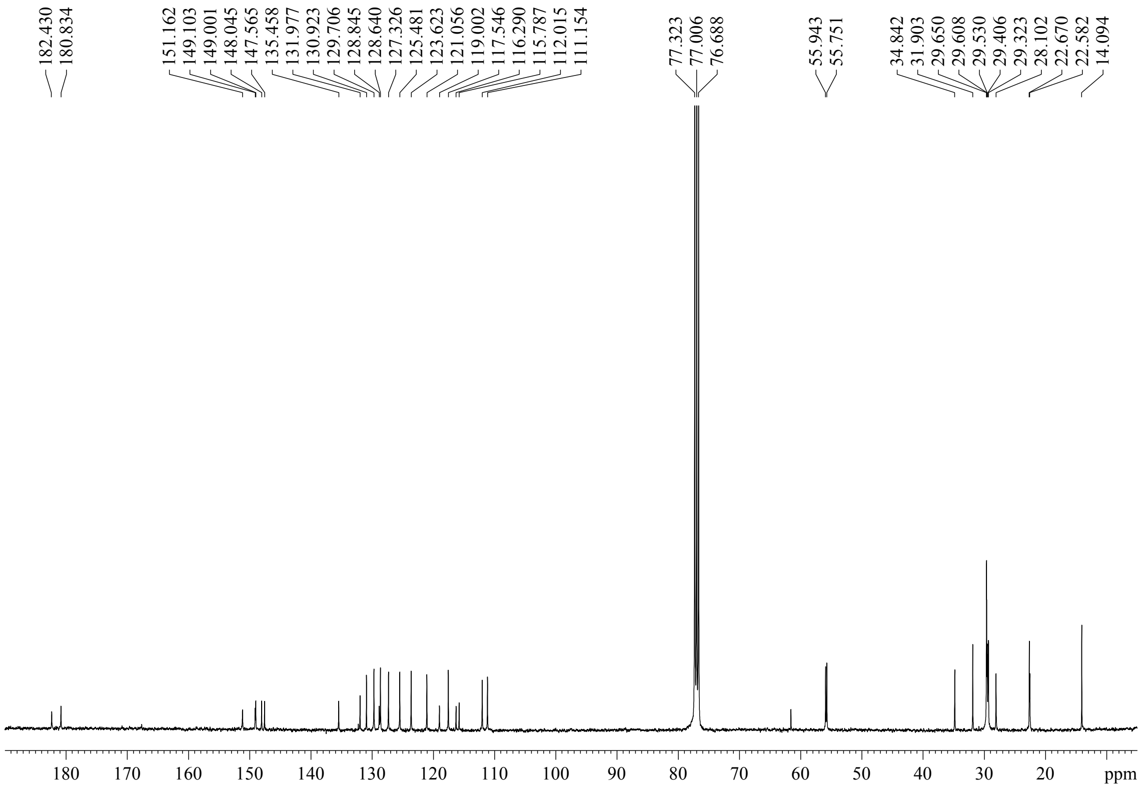

^1H NMR (C_6D_6, 400 MHz) of compound 5h

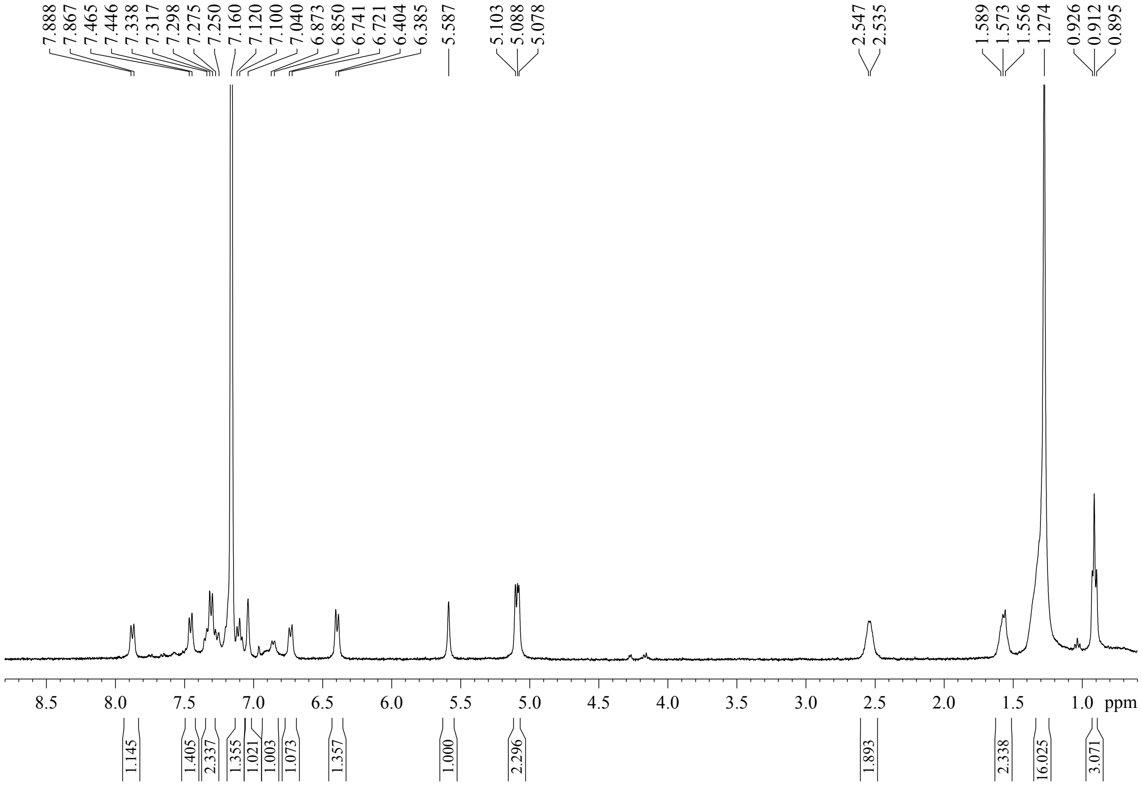

^{13}C NMR ($CDCl_3$, 100 MHz) of compound 5h

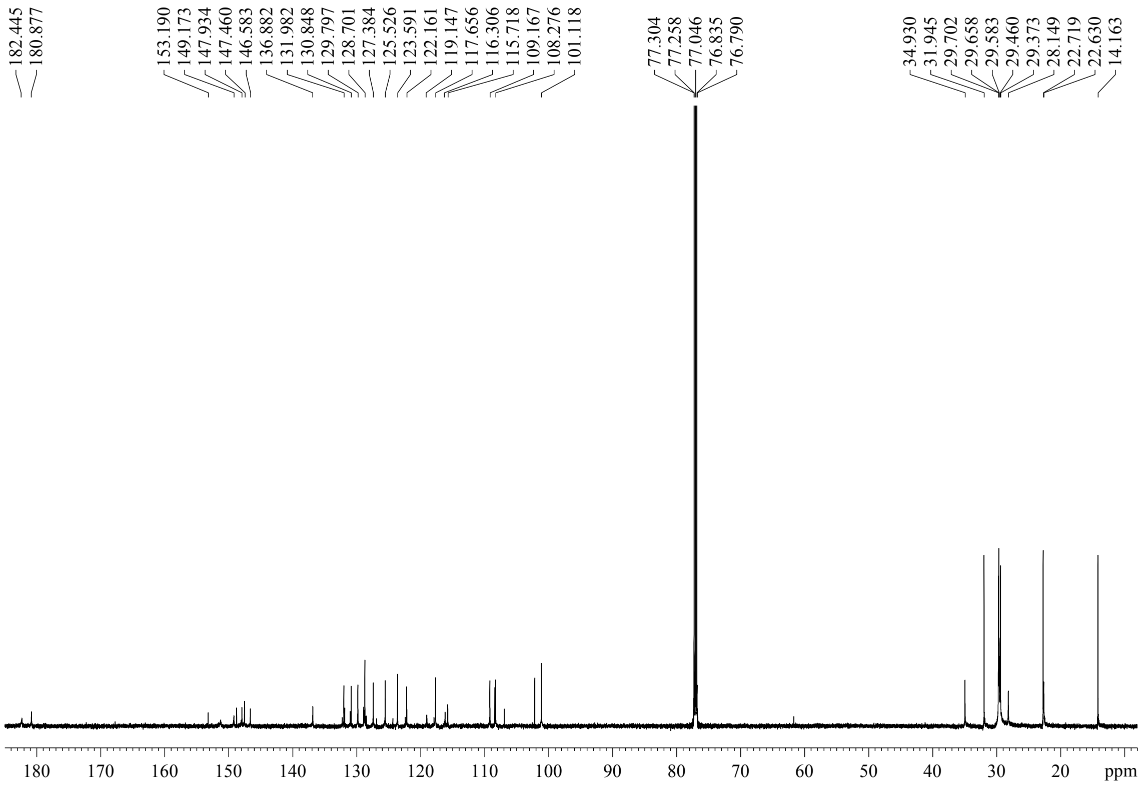

^{1}H NMR (C_6D_6, 400 MHz) of compound 5i

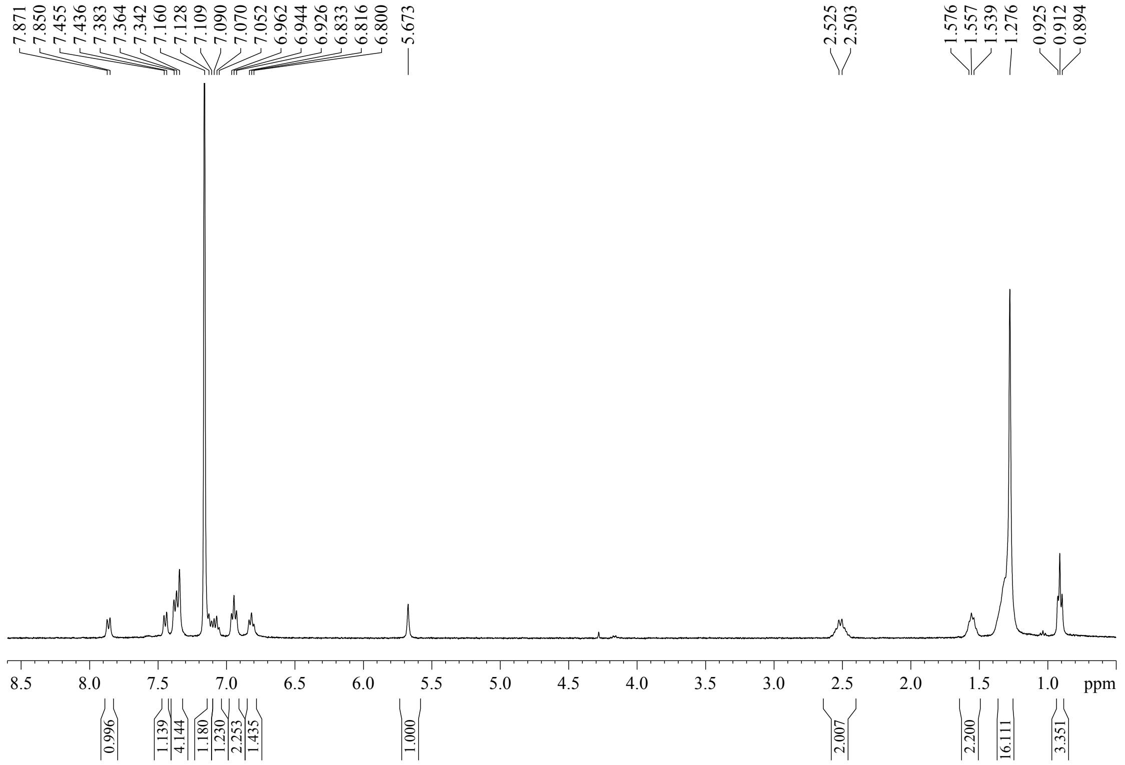

^{1}H NMR ($CDCl_3$, 500 MHz) of compound 5j

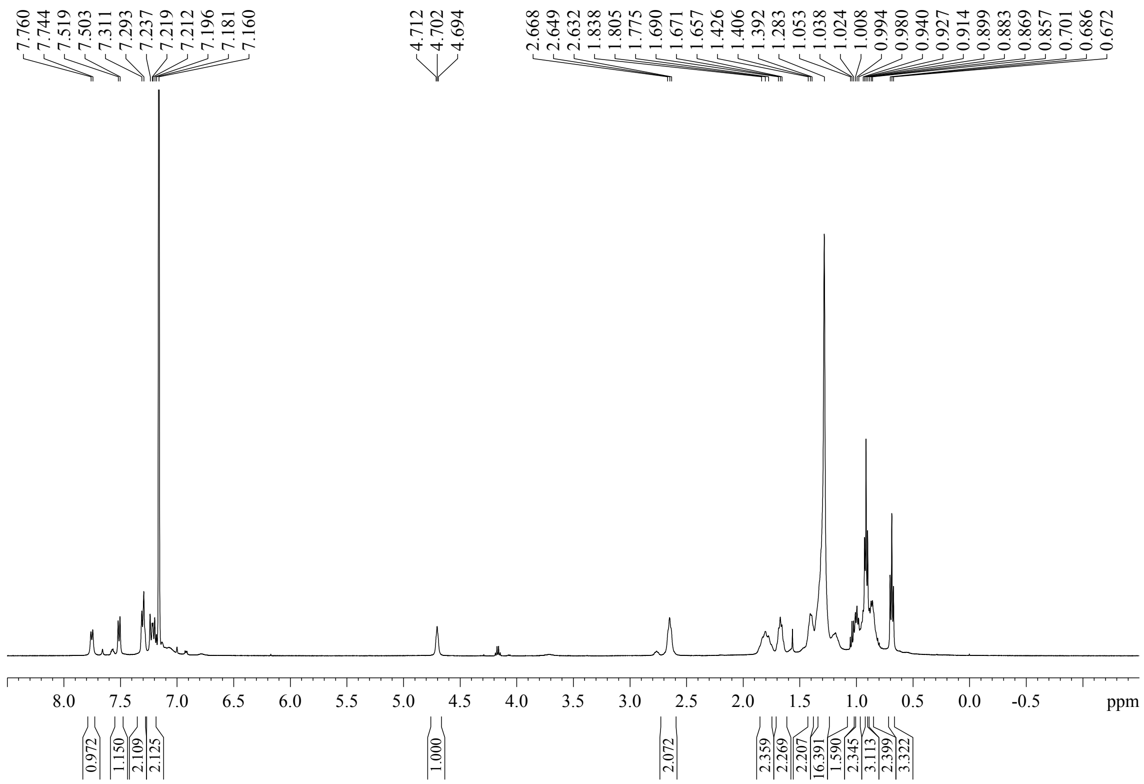

^{13}C NMR ($CDCl_3$, 100 MHz) of compound 5j

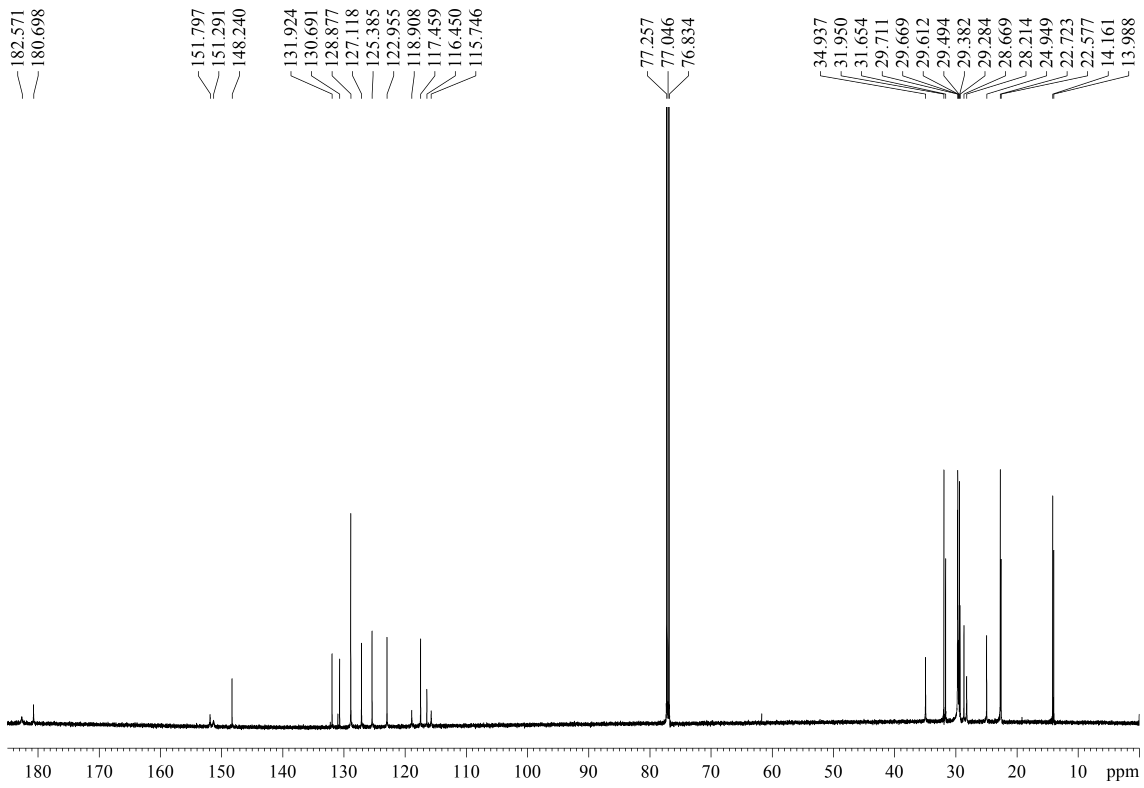

^{1}H NMR ($CDCl_3$, 400 MHz) of compound 5k

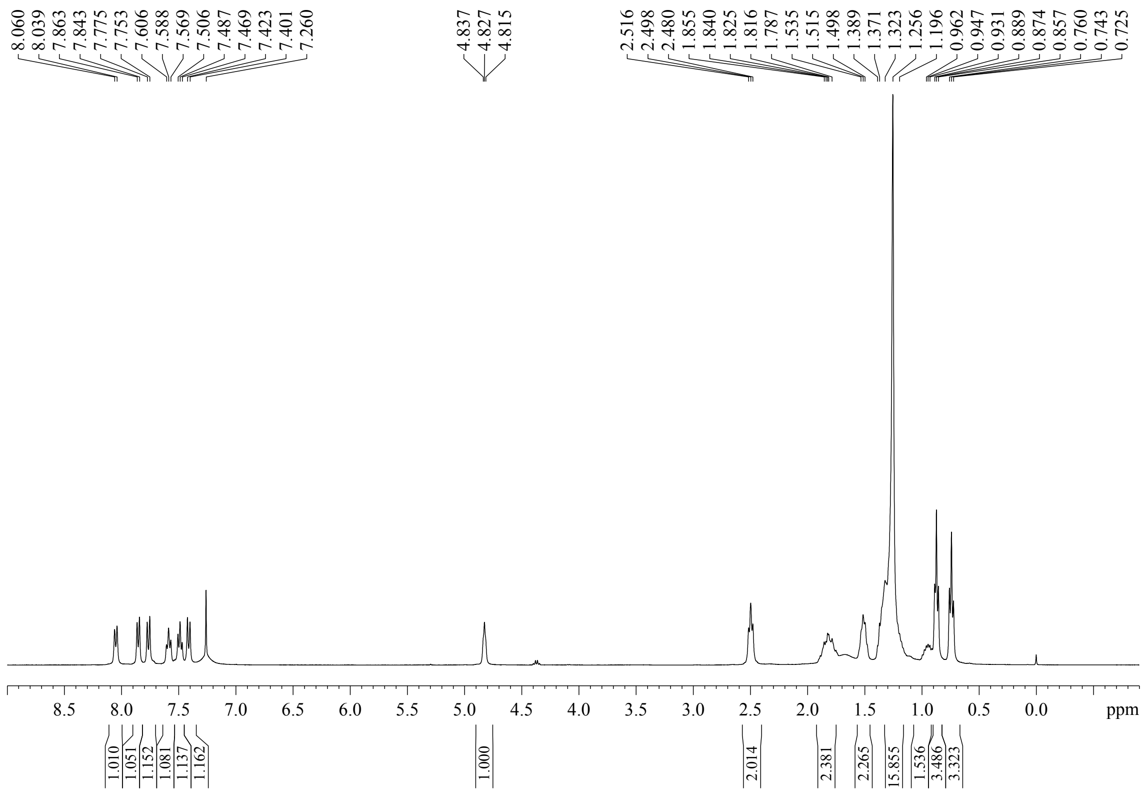

^{13}C NMR ($CDCl_3$, 100 MHz) of compound 5k

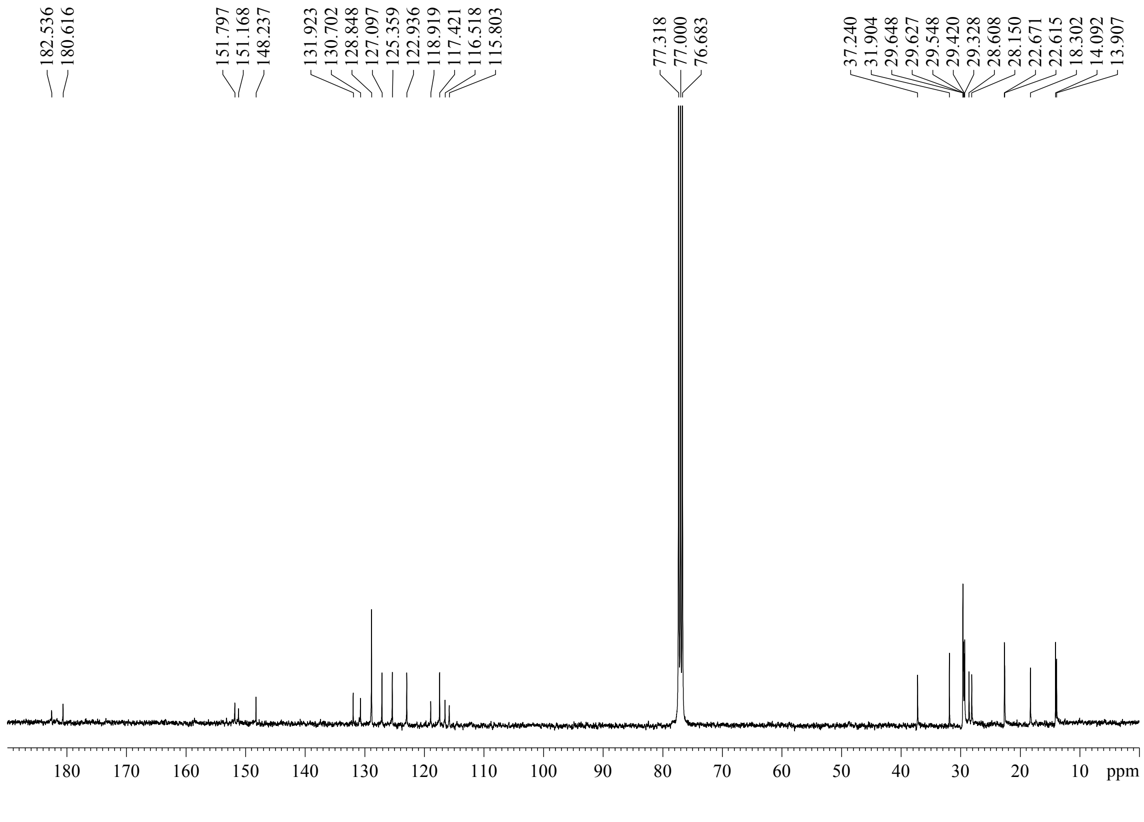

^{1}H NMR ($CDCl_3$, 400 MHz) of compound 5l

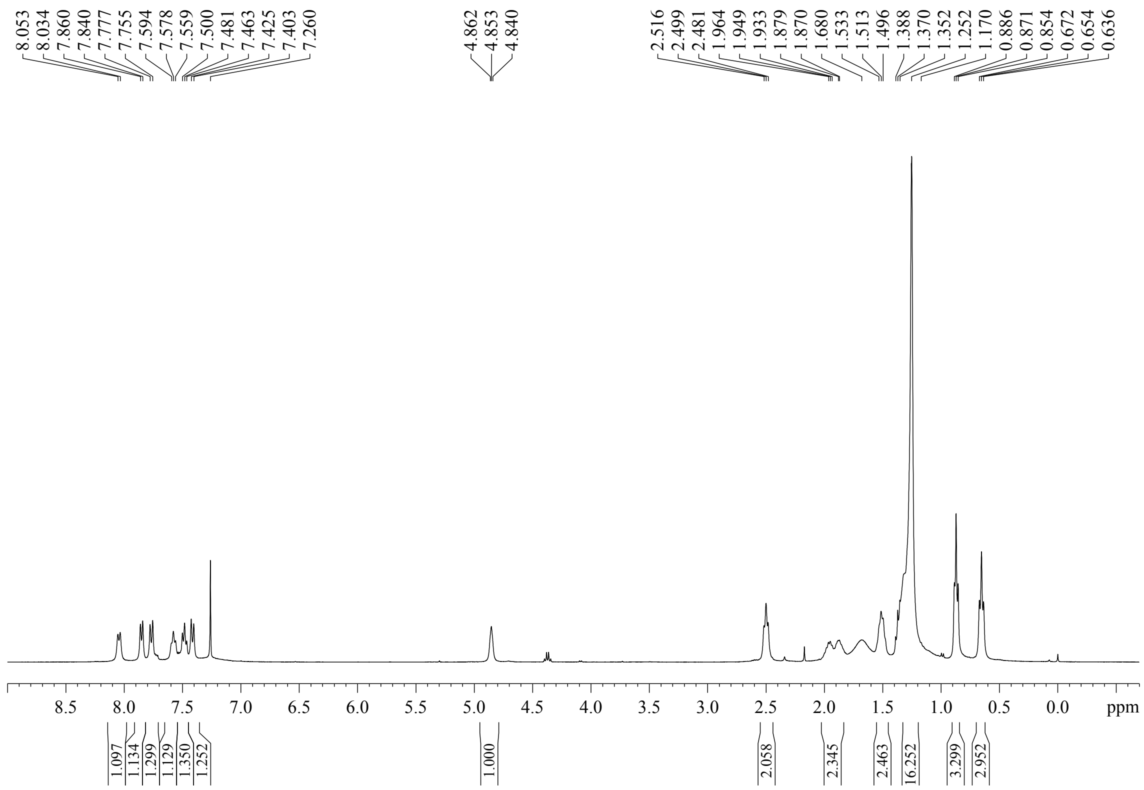

^{13}C NMR ($CDCl_3$, 100 MHz) of compound 5l

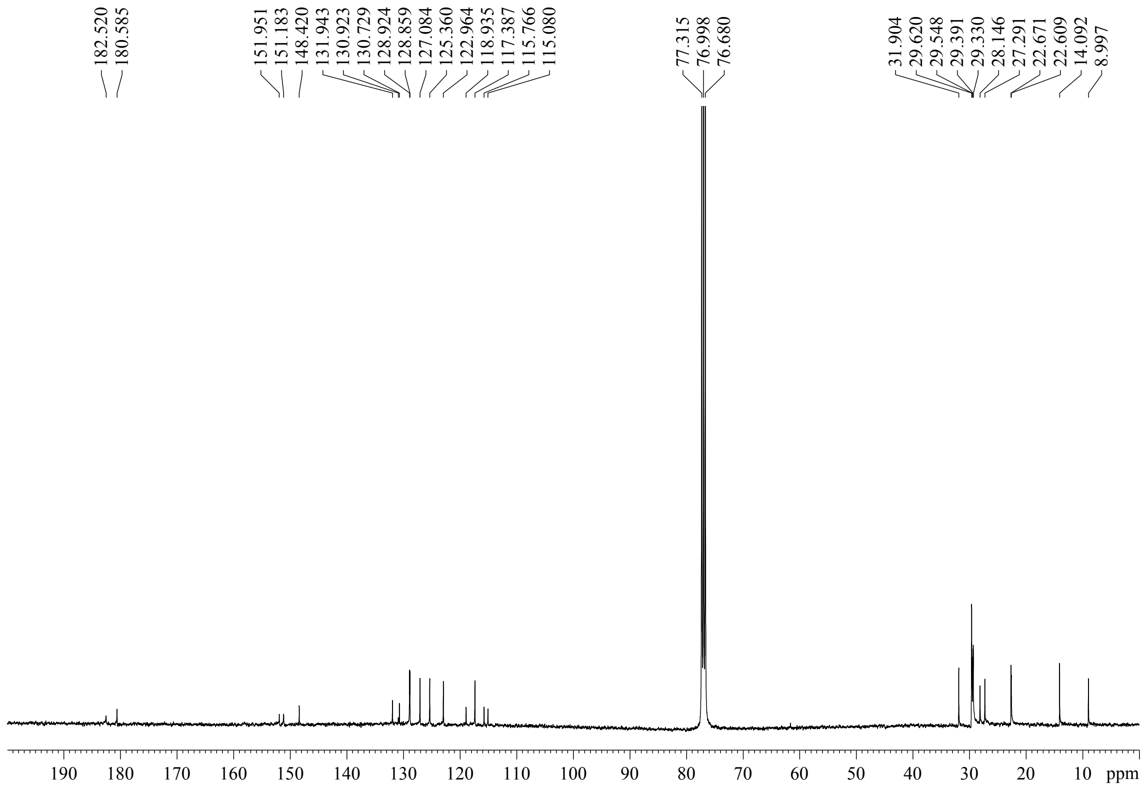

Supplement: Supplementary file 1 [file molecules-25-03290-s001.pdf]
